# Systematic Analysis of 2′-*O*-Alkyl Modified Analogs for Enzymatic Synthesis and Their Oligonucleotide Properties

**DOI:** 10.3390/molecules28237911

**Published:** 2023-12-02

**Authors:** Kenta Ishida, Yuuya Kasahara, Hidekazu Hoshino, Takumi Okuda, Satoshi Obika

**Affiliations:** 1 National Institutes of Biomedical Innovation, Health and Nutrition (NIBIOHN), 7-6-8 Saito-Asagi, Ibaraki 567-0085, Osaka, Japan; kenta-ishida@nibiohn.go.jp (K.I.); h-hoshino@nibiohn.go.jp (H.H.);; 2 Graduate School of Pharmaceutical Sciences, Osaka University, 1-6 Yamadaoka, Suita 565-0871, Osaka, Japan

**Keywords:** enzymatic oligonucleotide synthesis, aptamers, 2′-*O*-alkyl modifications, polymerase mutants

## Abstract

Enzymatic oligonucleotide synthesis is used for the development of functional oligonucleotides selected by in vitro selection. Expanding available sugar modifications for in vitro selection helps the functional oligonucleotides to be used as therapeutics reagents. We previously developed a KOD DNA polymerase mutant, KOD DGLNK, that enzymatically synthesized fully-LNA- or 2′-*O*-methyl-modified oligonucleotides. Here, we report a further expansion of the available 2′-*O*-alkyl-modified nucleotide for enzymatic synthesis by KOD DGLNK. We chemically synthesized five 2′-*O*-alkyl-5-methyluridine triphosphates and incorporated them into the oligonucleotides. We also enzymatically synthesized a 2′-*O*-alkyl-modified oligonucleotide with a random region (oligonucleotide libraries). The 2′-*O*-alkyl-modified oligonucleotide libraries showed high nuclease resistance and a wide range of hydrophobicity. Our synthesized 2′-*O*-alkyl-modified oligonucleotide libraries provide novel possibilities that can promote the development of functional molecules for therapeutic use.

## 1. Introduction

Enzymatic oligonucleotide synthesis is applied for the development of functional oligonucleotides so that their structure can contribute to their activities such as aptamers and nucleic acid enzymes. Aptamers are single-stranded oligonucleotides that specifically bind target molecules [1]. Nucleic acid enzymes are single-stranded oligonucleotides that have catalytic activities such as the site-specific cleavage of RNA [2]. These functional molecules are selected from 10^12–15^ unique oligonucleotide sequences (oligonucleotide library) using in vitro selection [3,4]. Since these functional oligonucleotides mainly consist of DNA or RNA, they are easily digested by nuclease [1,2], which diminishes the potential of using them as therapeutic agents.

The chemical modification of functional oligonucleotides enhances their properties such as their nuclease stability and binding affinity to target molecules. Therefore, expanding the available modified nucleotides for in vitro selection helps to develop functional oligonucleotides. Many nucleobase analogs have been developed and incorporated into aptamers to enhance their binding affinity. For example, incorporating hydrophobic nucleobase analogs like 5-modified uridine analogs into aptamers has been shown to improve their binding affinity [5,6,7,8]. On the other hand, since nucleobase-modified analogs contribute less to improving nuclease stability [9], the expansion of available sugar modifications for in vitro selection is necessary to promote the development of the functional molecules for therapeutic use. However, wild-type polymerases have a low tolerance for sugar modifications.

To expand the available sugar modifications for in vitro selection, we focused on 2′-*O*-alkyl modifications. Since the 2′-*O*-methyl (2′-OMe)-modified oligonucleotide exhibited a higher resistance against nucleases and higher thermal duplex stability for RNA than unmodified DNA, 2′-OMe modification has been used for oligonucleotide therapeutics [10,11]. Moreover, many 2′-*O*-alkyl-modified analogs have also been developed and investigated for their impact on oligonucleotides [12,13,14,15,16,17]. For instance, 2′-*O-*linear-carbon-chain-modified analogs, such as 2′-*O*-ethyl (2′-OEt), 2′-*O*-propyl (2′-OPr), and 2′-*O*-butyl (2′-OBu) modifications, tended to show higher nuclease stability depending on their substituted carbon chain length [13]. On the other hand, the 2′-*O*-substitution of a longer linear carbon chain tended to decrease the duplex stability of RNA [14,15]. Furthermore, the substitution of single carbon to oxygen in their alkyl chain, such as 2′-*O*-methoxyethyl (2′-MOE) and 2′-*O*-hydroxyethyl (2′-HE) modifications, enhance the duplex stability of RNA [14,16]. Moreover, 2′-MOE showed higher nuclease stability than the 2′-OMe modification [17]. The 2′-*O-*branched-carbon-chain-modified analog, 2′-*O*-isopropyl modification (2′-O*^i^*Pr), decreased the duplex stability of RNA [12,14].

Although several wild-type polymerases have the potential to polymerize sugar-modified nucleotides [18,19,20], sugar modification is still challenging to incorporate by wild-type DNA polymerase. A combination of multiple mutations in thermostable DNA polymerases can improve the polymerization of 2′-*O*-substituted nucleotides. Several polymerase mutants have been developed to synthesize 2′-OMe-modified oligonucleotides [21,22,23]. Chen et al. developed Taq DNA polymerase mutants which could synthesize fully-2′-OMe-modified oligonucleotides [21]. Moreover, Freund et al. recently developed Tgo polymerase mutants, which enzymatically synthesized not only fully-2′-OMe-modified oligonucleotides but also 2′-MOE-modified oligonucleotides [22]. We also developed a KOD polymerase mutant, KOD DGLNK, for LNA-modified oligonucleotide synthesis, which can also efficiently synthesize fully-2′-OMe-modified oligonucleotides [23]. KOD DGLNK had multiple mutations (N210D, Y409G, A485L, D614N, and E664K). The N210D substitution reduces exonuclease activity [24]. The Y409 residue is predicted to sterically clash with the 2′-OH of ribonucleotide triphosphates [25]. The Y409G substitution reduces this steric clash. The A485L substitution promotes the sending of triphosphates to the active site [26]. The D614N and E664K substitutions enhance the binding affinity between the polymerase and primer-template duplex [23,26,27]. These multiple mutations significantly promote the synthesis of fully-LNA- and 2′-OMe-modified oligonucleotides. Additionally, we developed a 2′-OMe-and-LNA-mix aptamer [23]. However, there has been no systematic analysis of the tolerance range for the enzymatic synthesis of the 2′-*O*-alkyl modifications. And the properties of synthesized oligonucleotide will also be of interest.

**Figure 1:**
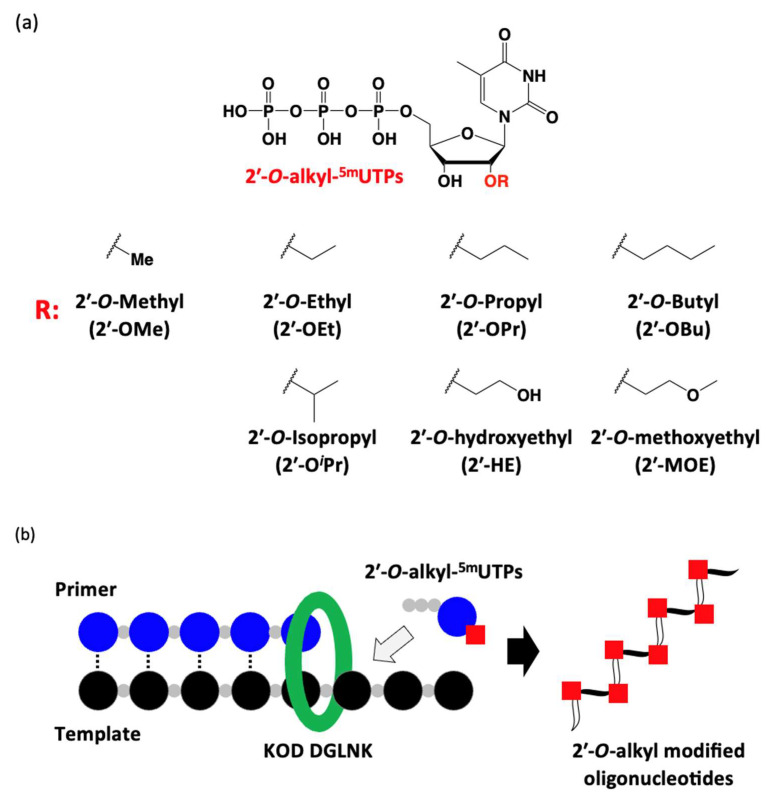
(**a**) Chemical structures of the 2′-*O*-alkyl-5-methyluridine triphosphates (2′-*O*-alkyl-^5m^UTPs) that we used in this study. (**b**) Schematic illustration of the incorporation of 2′-*O*-alkyl-^5m^UTPs into an oligonucleotide.

Here, we synthesized five 2′-*O*-alkyl-5-methyluridine triphosphates (2′-*O*-ethyl 5-methyuridine triphosphate [2′-OEt-^5m^UTP], 2′-*O*-propyl 5-methyuridine triphosphate [2′-OPr-^5m^UTP], 2′-*O*-butyl 5-methyuridine triphosphate [2′-OBu-^5m^UTP], 2′-*O*-isopropyl 5-methyuridine triphosphate [2′-O*^i^*Pr-^5m^UTP], and 2′-*O*-hydroxyethyl 5-methyuridine triphosphate [2′-HE-^5m^UTP]) and incorporated them into oligonucleotides by KOD DGLNK (Figure 1). Moreover, we evaluated the nuclease stability and hydrophobicity of the resulting 2′-*O*-alkyl-modified oligonucleotides.

## 2. Results and Discussion

### 2.1. Synthesis of 2′-O-Alkyl-ribonucleotide Triphosphates

KOD DGLNK has been modified based on DNA polymerase, and it lacks mutations that enhance its tolerance for uridine. Consequently, we opted for 5-methyluridine (thymidine), a component of DNA, and not uridine, a component of RNA, as the substrate for KOD DGLNK. Subsequently, we synthesized 2′-OEt-^5m^UTP, 2′-OPr-^5m^UTP, 2′-OBu-^5m^UTP, 2′-O*^i^*Pr-^5m^UTP, and 2′-HE-^5m^UTP. The 2′-*O*-alkylations were systematically performed with reference to the methods previously used to synthesize other 2′-*O*-alkyl-U or ^5m^Us [16,28]. The synthesis of 2′-OEt-^5m^UTP, 2′-OPr-^5m^UTP, 2′-OBu-^5m^UTP and 2′-O*^i^*Pr-^5m^UTP were started from 3′-,5′-silyl- and *N*^3^-benzoyl-protected 5-methyluridine [29]. The 2′-hydroxy group was alkylated via a nucleophilic attack of the 2′-hydroxy group to the corresponding alkyl iodide. After switching the protection group, the resulting 3′-acetyl- and *N*^3^-benzoyl-protected 2′-*O*-alkyl-5-methyluridines were substituted to triphosphates using the Ludwig and Eckstein method [30] (Scheme S1). The synthesis of 2′-HE-^5m^UTP was started from 2,2′-anhydro-5-methyluridine. After TBS protection of the 5′-hydroxy group, the alkylation of the 2′-hydroxy group was performed by hydroxyethylation using trihydroxyethyl borate esters. This was followed by switching the protection group, resulting in 3′- and 2′-*O*-hydroxyethyl*-*acetyl-protected 5-methyuridine being substituted to triphosphate in the same manner (Scheme S2). All the synthesized triphosphates were purified by the ion pair RP-HPLC.

### 2.2. Polymerase Incorporation of 2′-O-Alkyl 5-methyluridine Triphosphates into Oligonucleotide

**Figure 2:**
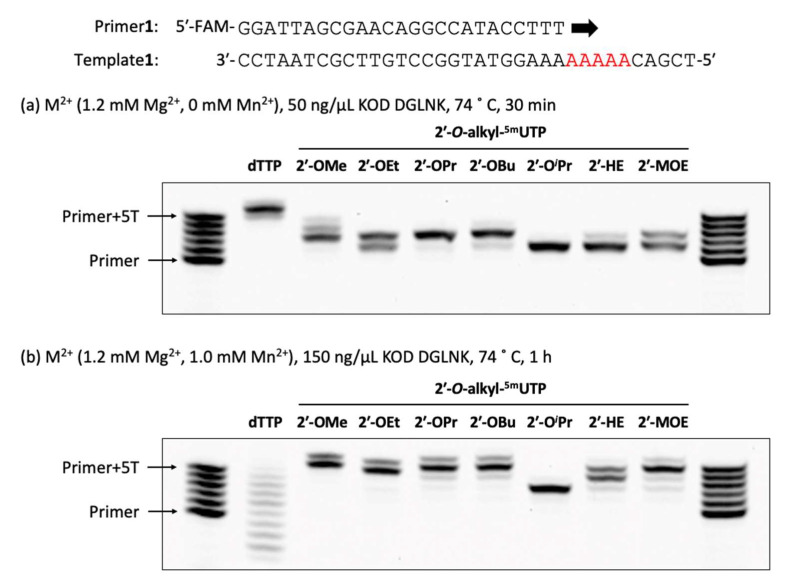
Polymerase incorporation of 2′-*O*-alkyl-^5m^UTPs with a DNA primer. (**a**) Primer extension was performed with a 0.4 µM 5′-FAM-labeled DNA primer, 0.5 µM DNA template, 1× KOD Dash^®^ buffer, and 0.2 mM 2′-*O*-alkyl-^5m^UTP by incubation with 50 ng/µL KOD DGLNK at 74 °C for 30 min. (**b**) Primer extension was performed with a 0.4 µM 5′-FAM-labeled DNA primer, 0.5 µM DNA template, 1 × KOD Dash^®^ buffer, 1.0 mM MnSO_4,_ and 0.2 mM 2′-*O*-alkyl-^5m^UTPs by incubation with 150 ng/µL KOD DGLNK at 74 °C for 1 h.

First, we evaluated the incorporation efficiency of 2′-*O*-alkyl-^5m^UTPs using a primer extension assay against a DNA template (Template**1**) which had a 5nt consecutive adenosine. An extended FAM-labeled DNA primer (Primer**1**) was analyzed by denaturing polyacrylamide gel electrophoresis (PAGE) (Figure 2). Following an incubation period of 30 min at 74 °C with KOD DGLNK (50 ng/µL), we obtained a fully extended product with dTTP, and all of the 2′-*O*-alkyl-^5m^UTPs showed at least single nucleotide incorporation (Figure 2a). This result suggests that 2′-*O*-alkyl-^5m^UTPs potentially works as substrates for oligonucleotide polymerization. During the comparison of the relationship between the length of alkyl chains and incorporation efficiency, 2′-OBu-^5m^UTP, which is the longest alkyl chain, surprisingly showed almost the same incorporation efficiency as 2′-OMe-^5m^UTP. The efficiency of incorporation improved according to the length of the alkyl chain (incorporation efficiency: 2′-OBu-^5m^UTP > 2′-OPr-^5m^UTP > 2′-OEt-^5m^UTP) (Figure S1). On the other hand, the substitution of a carbon atom for an oxygen atom resulted in decreased incorporation efficiency at any carbon length (incorporation efficiency: 2′-OBu-^5m^UTP > 2′-MOE-^5m^UTP, 2′-OPr-^5m^UTP > 2′-HE-^5m^UTP) (Figure S1). We saw that 2′-MOE-^5m^UTP demonstrated higher incorporation efficacy into oligonucleotides than 2′-HE-^5m^UTP (Figure S1), and 2′-O*^i^*Pr-^5m^UTP showed the lowest incorporation efficiency (Figure S1). Although the steric hindrance of the 2′-*O*-alkyl modification is known to reduce incorporation efficiency [22], 2′-OBu-^5m^UTP was more effectively incorporated into DNA than 2′-OEt-^5m^UTP. Our results indicated that factors other than steric hindrance also impact the incorporation efficiency of 2′-*O*-alkyl modifications. Next, to enhance the extension efficiency of 2′-*O*-alkyl-^5m^UTPs, we attempted to optimize reaction conditions. The addition of Mn^2+^ accelerates the incorporation of modified nucleotide triphosphates [21,27,31]. Under stronger conditions such as a higher amount of KOD DGLNK (150 ng/µL), the addition of Mn^2+^ (1.0 mM), and a longer incubation time (1 h), fully extended products were observed, except for 2′-O*^i^*Pr, in the incorporation of 2′-*O*-alkyl-^5m^UTPs (Figure 2b). On the other hand, during the incorporation of dTTP, we observed ladder-like bands. Moreover, we observed bands under the primer bands. These bands were probably caused by competition between the polymerization activity and exonuclease activity of KOD DGLNK. Extended products were also observed when Primer**1** was replaced with Primer**2,** which was modified with 2′-OMe modifications (Figure S3).

### 2.3. Enzymatic Synthesis of 2′-O-alkyl-Modified Oligonucleotide Libraries

**Figure 3:**
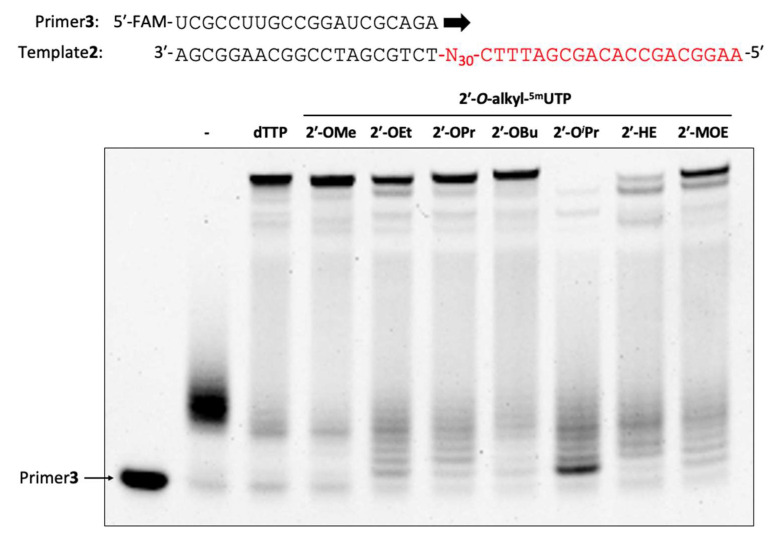
Enzymatic synthesis of 2′-*O*-alkyl-modified oligonucleotide libraries. Primer extension was performed with a 0.4 µM 5′-FAM-labeled 2′-OMe-modified primer, 0.5 µM DNA template, 1× KOD Dash^®^ buffer, 1.0 mM MnSO_4_, 0.2 mM 2′-OMe-ATP, 0.2 mM 2′-OMe-GTP, 0.2 mM 2′-OMe-CTP, and 2′-*O*-alkyl-^5m^UTP by incubation with 300 ng/µL KOD DGLNK at 60 °C for 1 h, 60 °C to 74 °C for 4 h, and 74 °C for 1 h. Following the extension, the reaction mixture was treated with 95 mU/µL DNase1 at 37 °C for 30 min.

After the characterization of 2′-*O*-alkyl-^5m^UTPs for a short-length template, a fully modified 2′-*O*-alkyl-modified oligonucleotide synthesis for a longer template which had a 70 nt DNA sequence with a random region in the center (2′-*O*-alkyl-modified oligonucleotide libraries) was tested. For the incorporation of 50 nucleotide triphosphates, stronger reaction conditions than the reaction conditions for the incorporation of 5 nucleotide triphosphates are assumed to be required. However, such drastic conditions induce digestion on the part of DNA as we observed in Figure 2b. Therefore, primer extension was performed with 2′-OMe-modified primers with 2′-OMe-ATP, 2′-OMe-GTP, 2′-OMe-CTP, and synthesized 2′-*O*-alkyl-^5m^UTPs for giving exonuclease resistance to the extended oligonucleotide. In the random region, averagely, a 25% nucleotide would contain a target alkyl modification at the end. As we expected, extension efficiency was shown with the same tendency as the result of the short-length template (Figure 3). Except for the 2′-O*^i^*Pr modification, fully extended products were observed. Furthermore, by increasing the Mn^2+^ concentration and reaction time, the 2′-O*^i^*Pr and 2′-HE modifications were also nicely incorporated and full-length products were observed under 3.0 mM of Mn^2+^ following 4 h of incubation (Figure S4). We noticed that, when Mg^2+^ and Mn^2+^ coexist, 3.0 mM or more of Mn^2+^ promotes the enzymatic synthesis of 2′-*O*-alkyl-modified oligonucleotides (Figure S4). The enzymatically synthesized 2′-*O*-alkyl-modified oligonucleotide library that include 2′-OEt-5-methyluridine was named ON_Et. Moreover, other 2′-*O*-alkyl-modified oligonucleotide libraries also named as the same manner (ON_Me, ON_Pr, ON_Bu, ON_*^i^*Pr, ON_HE, and ON_MOE).

### 2.4. Properties of the 2′-O-Alkyl-Modified Oligonucleotide Libraries

#### 2.4.1. Hydrophobicity of the 2′-O-Alkyl-Modified Oligonucleotide Libraries

**Figure 4:**
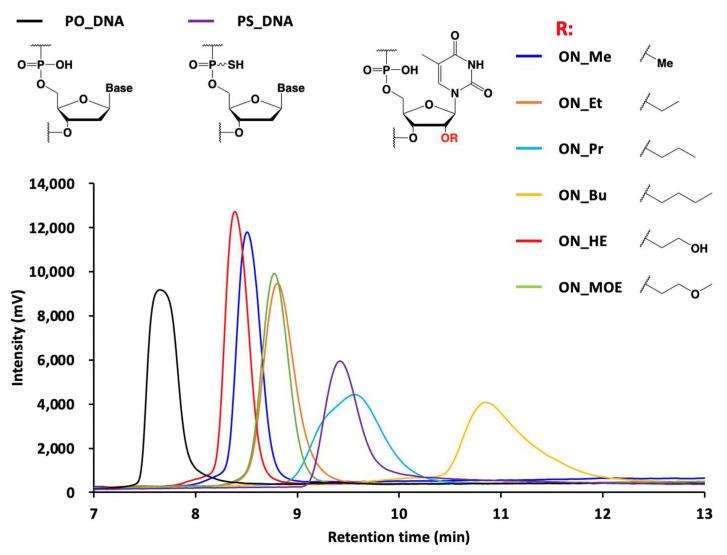
Hydrophobicity analysis of 2′-*O*-alkyl-modified oligonucleotide libraries. Ion pair RP-HPLC was performed by the injection of 5.0 pmol of oligonucleotide libraries onto an XBridge Oligonucleotide BEH C18 column. The column temperature was 50 °C, with a flow rate of 1.0 mL/min, and detection at 260 nm. Mobile phase A was a 100 mM triethylamine acetic acid solution (pH 6.9), and mobile phase B was acetonitrile. The column was initially maintained at 10% mobile phase B, and then at a gradient of 10–40% over 15 min.

The hydrophobicity of the oligonucleotide libraries was evaluated by the retention time on the ion pair RP-HPLC (Figure 4). The longer alkyl chain modification increases the interaction between the oligonucleotide and C18 modification on the reverse phase column, resulting in slow elution. The substitution of carbon to oxygen in the alkyl chain induced fast elution in the column. The hydrophobic tendency of nucleotide triphosphates is reflected in the hydrophobic tendency of the oligonucleotide libraries (Figure S5). ON_Pr was shown to be hydrophobic as the fully-phosphorothioate-modified DNA library (PS_DNA). With only the substitution of 5-methyluridine, ON_Bu was shown to be more hydrophobic than PS_DNA. This property is highly beneficial in terms of expanding the hydrophobic range.

#### 2.4.2. Nuclease Stability of the 2′-O-Alkyl-Modified Oligonucleotide Libraries

**Figure 5:**
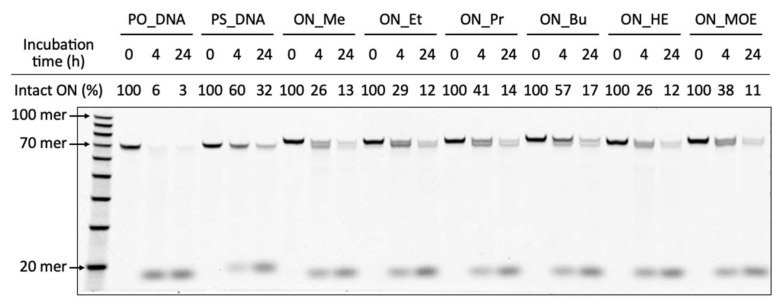
Nuclease stability of the 2′-*O*-alkyl-modified oligonucleotide libraries. FBS digestion of the oligonucleotides was performed with 0.1 µM 5′-6-FAM-labeled oligonucleotides, by incubation with 50% FBS at 37 °C for 0, 4, and 24 h followed by inactivation steps: 95 °C for 5 min. Proteinase K digestion was performed with an FBS digested reaction mixture by incubation with 36 mU/µL Proteinase K at 37 °C for 30 min.

The nuclease stability of the 2′-*O*-alkyl-modified oligonucleotide libraries in 50% FBS was evaluated by denaturing PAGE (Figure 5). Following 4 h of incubation, the intact and digested products were observed. All the 2′-*O*-alkyl-modified oligonucleotides were more stable than PO_DNA. ON_Bu was as stable as PS_DNA. The substitution of the longer 2′-*O*-alkyl 5-methyluridine of the oligonucleotides tended to show a higher stability in 50% FBS (stability: ON_Bu > ON_Pr > ON_Et > ON_Me). The replacement of a carbon atom for an oxygen atom reduced stability in 50% FBS (stability: ON_Bu > ON_MOE, ON_Pr > ON_HE). On the other hand, after 24 h of incubation, the ratio of the intact product was a little different. This was probably caused by the digestion of the 2′-OMe-modified primer region.

Expanding the available chemical modifications to enzymatic oligonucleotide synthesis accelerates the development of aptamers and nucleic acid enzymes. By mixing 2′-OMe-adenosine, guanosine, and cytidine, oligonucleotide libraries containing 2′-OEt, 2′-OPr-, 2′-OBu or 2′-HE-5-methyluridine were successfully synthesized by KOD DGLNK (Figure 3). This indicated that KOD DGLNK is tolerant of 2′-*O*-linear alkyl modifications in polymerization. On the other hand, KOD DGLNK showed a lower tolerance for 2′-*O*-branched alkyl modification, such as 2′-O*^i^*Pr modification, than 2′-*O*-linear alkyl modifications. It would be interesting to explore the kinetics of the incorporation and identify the novel mutations associated with a tolerance for 2′-*O*-branched alkyl modification. Additionally, we investigated the impact of a partial substitution of 2′-*O*-alkyl modification on the nuclease stability and hydrophobicity of the oligonucleotide libraries. Despite only incorporating a 5-methyluridine base into the oligonucleotides, ON_Et, ON_Pr, ON_Bu, ON_HE, and ON_MOE demonstrated a different hydrophobicity (Figure 4). This variety of hydrophobicity difference can be a toolbox which offers the option for aptamer selection for individual targets. Enzymatic stability is also an important factor in the efficacy of aptamers and nucleic acid enzymes in vitro and in vivo. Indeed, approved antisense oligonucleotides and siRNA contain chemical modifications to improve nuclease stability [11]. Moreover, the approved aptamers, Macugen (pegaptanib) [32] and IZERVAY (avacincaptad pegol) [33,34], have sugar modifications (2′-OMe and 2′-fluoro modifications) and 40 kDa PEG modifications by post-modification. However, following post-modification, even partial modification sometimes reduces their binding affinity to the target molecules. On the other hand, the 2′-*O*-alkyl-modified oligonucleotide libraries showed higher nuclease stability than DNA (Figure 5). Moreover, ON_Bu showed the equivalent stability to PS-modified DNA. These results indicate that our study can help develop high nuclease-stable functional oligonucleotides without post-modification. To develop such functional oligonucleotides through in vitro selection, it is important to explore the conversion of 2′-*O*-alkyl-modified oligonucleotide libraries to DNA. Furthermore, it is important to investigate the error rate of incorporation because the addition of Mn^2+^ for the synthesis of 2′-O-alkyl-modified oligonucleotide libraries reduces fidelity [35]. The 2′-*O*-alkyl-modified oligonucleotide libraries exhibited a wide range of hydrophobicity and improvement of nuclease stability by only the replacement of 5-methyluridines. The synthesis of 2′-*O*-alkyl-^5m^UTPs was systematic and easy; hence, our strategy can be applied for the synthesis of other 2′-*O*-alkyl ^5m^UTPs. Moreover, by applying the 2′-*O*-alkyl-modified oligonucleotides that are synthesized in this study to not only 5-methyluridine but also other nucleobases, 2′-*O*-alkyl-modified oligonucleotide libraries can show a further wide range of hydrophobicity and enhancement nuclease stability.

## 3. Materials and Methods

### 3.1. General Information

All reagents and solvents were purchased from commercial suppliers and used without purification unless otherwise specialized. All reactions were carried out under Ar atmosphere. PAGE images were recorded on Chemi-Doc (Bio-Rad, Hercules, CA, USA) and analyzed by Image Lab^TM^ (Bio-Rad). ^1^H, ^13^C and ^31^P NMR spectra were recorded on an AVHD 400 NB (Bruker Daltonics, Billerica, MA, USA) using CDCl_3_, DMSO-*d*_6_, and D_2_O as the solvents. Mass spectra of all new compounds were measured on a JMS-700 instrument (JEOL, Tokyo, Japan) (for fast atom bombardment, FAB). For HPLC, Shimadzu SLC-20A3R, LC-20AD, CTO-20AC, SPD-20A, and FRC-10A were utilized.

### 3.2. Synthesis of N^3^-Benzoyl-2′-O-ethyl-3′,5′-O-(1,1,3,3-tetraisopropyldisiloxane-1,3-diyl)-5-methyluridine (***2a***)

Compound **1** (1.5 g, 2.5 mmol) was dissolved in dry toluene (7.5 mL). Ag_2_O (1460 mg, 6.3 mmol) and iodoethane (5.0 mL, 63 mmol) were added and the reaction mixture was stirred at 60 °C for 13 h. The resulting reaction mixture was filtered by celite pad and the filtrate was diluted with CHCl_3_. The combined organic layers were washed with water and brine, dried over anhydrous Na_2_SO_4_, and concentrated in vacuo. The residue was purified by silica gel column chromatography (Hexane/Ethyl acetate = 85/15) to afford compound **2a** (456 mg, 0.72 mmol, 29%) as a white form. ^1^H NMR (400 MHz, CDCl_3_): δ 7.93 (dd, *J* = 1.2 Hz, 8.4 Hz, 2H), 7.73 (d, *J* = 1.2 Hz, 1H), 7.67–7.63 (m, 1H), 7.49 (t, *J* = 7.5 Hz, 2H), 5.70 (s, 1H), 4.29–4.21 (m, 2 H), 4.12 (dd, *J* = 2.0 Hz, 9.6 Hz, 1H), 3.87–3.77 (d, 3H), 1.95 (d, *J* = 1.1 Hz, 3H), 1.19 (t, *J* = 7.0 Hz, 3H), 1.21–1.05 (m, 28H). ^13^C NMR (100 MHz, CDCl_3_): δ 169.12, 162.95, 149.01, 135.11, 134.85, 131.52, 130.55, 129.17, 110.10, 89.79, 82.00, 81.88, 68.18, 66.97, 59.38, 17.48, 17.43, 17.31, 17.27, 17.21, 17.09, 17.04, 16.92, 15.34, 13.57, 12.91, 12.81, 12.77, 12.60. HRMS (MALDI): calcd for C_31_H_48_N_2_O_8_NaSi_2_ [M + Na]^+^ 655.2841, found 655.2844.

### 3.3. Synthesis of N^3^-Benzoyl-2′-O-ethyl-5-methyluridine (***3a***)

Compound **2a** (426 mg, 0.67 mmol) was dissolved in dry THF (6.7 mL). Then, 1 M TBAF in THF (2.0 mL, 2.0 mmol) was added and the reaction mixture was stirred at room temperature for 30 min. The resulting reaction mixture was concentrated in vacuo. The residue was purified by silica gel column chromatography (CHCl_3_/MeOH = 99/1 to 97/3) to afford compound **3a** (238 mg, 0.61 mmol, 91%) as a white form. ^1^H NMR (400 MHz, CDCl_3_): δ 7.92 (dd, *J* = 1.2 Hz, 8.4 Hz, 2H), 7.75 (s, 1H), 7.68–7.63 (m, 1H), 7.49 (t, *J* = 7.6 Hz, 2H), 5.78 (d, *J* = 2.8 Hz, 1H), 4.32 (d, *J* = 5.3 Hz, 1H), 4.09 (dd, *J* = 3.0 Hz, 5.2 Hz, 1H), 4.03 (dd, *J* = 2.2 Hz, 8.3 Hz, 2H), 3.90–3.83 (m, 2H), 3.67–3.59 (m, 1H), 2.73 (d, *J* = 6.2 Hz, 1H), 2.50 (br, 1H), 1.95 (d, *J* = 1.2 Hz, 3H), 1.23 (t, *J* = 7.0 Hz, 3H). ^13^C NMR (100 MHz, CDCl_3_): δ 168.77, 162.79, 149.31, 136.74, 135.18, 131.43, 130.49, 129.20, 110.88, 89.85, 84.80, 81.23, 68.35, 66.79, 61.10, 15.24, 12.61. HRMS (MALDI): calcd for C_19_H_22_N_2_O_7_Na [M + Na]^+^ 413.1319, found 413.1316.

### 3.4. Synthesis of 3′-O-Acetyl-N^3^-benzoyl-5′-O-dimethoxytrityl-2′-O-ethyl-5-methyluridine (***4a***)

Compound **3a** (220 mg, 0.56 mmol) was dissolved in dry pyridine (5.6 mL). DMTrCl (209 mg, 0.62 mmol) was added and the reaction mixture was stirred at room temperature for 6 h. DMAP (7 mg, 0.06 mmol) and Ac_2_O (262 µL, 2.8 mmol) were added and the reaction mixture was stirred at room temperature for 18 h. The resulting reaction mixture was concentrated in vacuo and diluted with ethyl acetate. The combined organic layers were washed with saturated NaHCO_3_ aq. and brine, dried over anhydrous Na_2_SO_4_, andconcentrated in vacuo. The residue was purified by silica gel column chromatography (Hexane/Ethyl acetate = 3/1 to 2/1) to afford compound **4a** (291 mg, 0.40 mmol, 71%) as a white form. ^1^H NMR (400 MHz, CDCl_3_): δ 7.94 (d, *J* = 7.2 Hz, 2H), 7.72 (d, *J* = 1.0 Hz, 1H), 7.64 (t, *J* = 7.4 Hz, 1H), 7.49 (t, *J* = 7.8 Hz, 2H), 7.41 (d, *J* = 7.2 Hz, 2H), 7.34–7.26 (m, 7H), 6.86 (dd, *J* = 1.9 Hz, 8.9 Hz, 4H), 6.03 (d, *J* = 4.4 Hz, 1H), 5.36 (t, *J* = 5.3 Hz, 1H), 4.32–4.28 (m, 2H), 3.81 (s, 6H), 3.76–3.69 (m, 1H), 3.63–3.56 (m, 2H), 3.39 (dd, *J* = 2.3 Hz, 11.0 Hz, 1H), 2.10 (s, 3H), 1.40 (s, 3H), 1.16 (t, *J* = 7.0 Hz, 3H). ^13^C NMR (100 MHz, CDCl_3_): δ 170.13, 168.85, 162.76, 158.85, 158.83, 149.41, 144.05, 135.12, 135.00, 131.59, 130.52, 130.14, 129.11, 128.18, 128.09, 127.31, 113.36, 113.35, 114.41, 87.59, 87.24, 81.16, 80.18, 70.58, 66.93, 62.09, 55.29, 20.68, 15.16, 11.82. HRMS (MALDI): calcd for C_42_H_42_N_2_O_10_Na [M + Na]^+^ 757.2732, found 757.2738.

### 3.5. Synthesis of 3′-O-Acetyl-N^3^-benzoyl-2′-O-ethyl-5-methyluridine (***5a***)

Compound **4a** (276 mg, 0.38 mmol) was dissolved in CH_2_Cl_2_ (3.8 mL). TFA (1.0 mL) was added and the reaction mixture was stirred at room temperature for 30 min. The resulting reaction mixture was concentrated in vacuo and diluted with CHCl_3_. The combined organic layers were washed with saturated NaHCO_3_ aq. and brine, dried over anhydrous Na_2_SO_4_, and concentrated in vacuo. The residue was purified by silica gel column chromatography (Hexane/Ethyl acetate = 1/1 to 1/2) to afford compound **5a** (125 mg, 0.29 mmol, 76%) as a white form. ^1^H NMR (400 MHz, CDCl_3_): δ 7.92 (dd, *J* = 1.1 Hz, 8.3 Hz, 2H), 7.66 (t, *J* = 7.4 Hz, 1H), 7.62 (br, 1H), 7.50 (t, *J* = 7.9 Hz, 2H), 5.69 (d, *J* = 4.8 Hz, 1H), 5.27 (t, *J* = 5.0 Hz, 1H), 4.37 (t, *J* = 5.0 Hz, 1H), 4.23–4.21 (m, 1H), 3.97 (dd, *J* = 1.8 Hz, 12.6 Hz, 1H), 3.76 (dd, *J* = 2.0 Hz, 12.6 Hz, 1H), 3.70–3.53 (m, 2H), 2.15 (s, 3H), 1.96 (d, *J* = 1.0 Hz, 3H), 1.15 (t, *J* = 7.0 Hz, 3H). ^13^C NMR (100 MHz, CDCl_3_): δ 170.50, 168.66, 162.72, 149.43, 137.33, 135.20, 131.41, 130.53, 129.21, 111.19, 91.09, 83.01, 79.14, 70.43, 66.94, 61.55, 20.83, 15.22, 12.66. HRMS (MALDI): calcd for C_21_H_24_N_2_O_8_Na [M + Na]^+^ 455.1425, found 455.1427.

### 3.6. Synthesis of 2′-O-Ethyl-5-methyluridine-5′-triphospahte (***6a***)

Compound **5a** was dissolved in dry pyridine and the solution was evaporated to dryness in vacuo. Compound **5a** (23 mg, 0.065 mmol) was dissolved in dry pyridine (0.25 mL) and 1,4-dioxane (0.25 mL). Then, 2-Chloro-4*H*-1,3,2-benzodioxaphosphorin-4-one (15 mg, 0.072 mmol) was added and the reaction mixture was stirred for 30 min at room temperature. Tris(tetrabutylammonium) hydrogen pyrophosphate (43 mg, 0.078 mmol) in DMF (0.5 mL) and tributylamine (52 µL, 0.22 mmol) were added and the reaction mixture was stirred for 1 h at room temperature. Next, 0.1 M I_2_ in pyridine/H_2_O (98:2, *v*/*v*) was added and the reaction mixture was stirred for 30 min at 0 °C. Then, 10% Na_2_S_2_O_3_ aq. was added and the reaction mixture was stirred for 10 min at room temperature. The reaction mixture was purified by ethanol precipitation. The precipitate was dissolved in 7% NH_3_ aq. and the reaction mixture was stirred for 2 h at room temperature. The solvent was removed in vacuo and the residue was purified by HPLC to give compound **6a** (14 µmol, 22%). ^1^H NMR (400 MHz, D_2_O): δ7.78 (s, 1H), 6.02 (d, *J* = 5.0 Hz, 1H), 4.53 (t, *J* = 4.5 Hz, 1H), 4.25 (d, *J* = 4.9 Hz, 3H), 4.19 (t, *J* = 5.2 Hz, 1H), 3.71 (dd, *J* = 7.0 Hz, 14.1 Hz, 2H), 1.92 (s, 3H), 1.18 (t, *J* = 7.0 Hz, 3H). ^31^P NMR (120 MHz, D_2_O): δ −9.60 (d, *J* = 14.5 Hz), −12.41 (d, *J* = 14.5 Hz), −23.38 (t, *J* = 14.7 Hz). HRMS (MALDI): calcd for C_12_H_20_N_2_O_15_P_3_ [M – H]^−^ 525.0082, found 525.0089.

### 3.7. Synthesis of N^3^-Benzoyl-2′-O-propyl-3′,5′-O-(1,1,3,3-tetraisopropyldisiloxane-1,3-diyl)-5-methyluridine (***2b***)

Compound **1** (1.5 g, 2.5 mmol) was dissolved in dry toluene (7.5 mL). Ag_2_O (1460 mg 6.3 mmol) and 1-iodopropane (6.0 mL, 63 mmol) were added and the reaction mixture was stirred at 80 °C for 15 h. The resulting reaction mixture was filtered by celite pad and the filtrate was diluted with CHCl_3_. The combined organic layers were washed with water and brine, dried over anhydrous Na_2_SO_4_, and concentrated in vacuo. The residue was purified by silica gel column chromatography (Hexane/Ethyl acetate = 85/15) to afford compound **2b** (369 mg, 0.57 mmol, 23%) as a white form. ^1^H NMR (400 MHz, CDCl_3_): δ 7.93 (dd, *J* = 1.2 Hz, 8.4 Hz, 2H), 7.73 (d, *J* = 1.2 Hz, 1H), 7.67–7.63 (m, 1H), 7.49 (t, *J* = 8.0 Hz, 2H), 5.71 (s, 1H), 4.29–4.21 (m, 2 H), 4.16 (dd, *J* = 2.1 Hz, 9.6 Hz, 1H), 3.84 (d, *J* = 4.3 Hz, 1H), 3.71 (t, *J* = 6.4 Hz, 2H), 1.95 (d, *J* = 1.1 Hz, 3H), 1.62–1.53 (m, 2H), 1.13–1.04 (m, 28H), 0.90 (t, *J* = 7.4 Hz, 3H). ^13^C NMR (100 MHz, CDCl_3_): δ 169.14, 162.98, 148.98, 135.11, 134.87, 131.53, 130.56, 129.17, 110.06, 89.65, 82.16, 81.90, 73.08, 68.24, 59.39, 23.01, 17.49, 17.43, 17.31, 17.28, 17.21, 17.09, 17.03, 16.93, 13.58, 12.91, 12.81, 12.77, 12.65. HRMS (MALDI): calcd for C_32_H_50_N_2_O_8_NaSi_2_ [M + Na]^+^ 699.2998, found 699.2986.

### 3.8. Synthesis of N^3^-Benzoyl-2′-O-propyl-5-methyluridine (***3b***)

Compound **2b** (185 mg, 0.29 mmol) was dissolved in dry THF (3.0 mL). 1 M TBAF in THF (0.86 mL, 0.86 mmol) was added and the reaction mixture was stirred at room temperature for 30 min. The resulting reaction mixture was concentrated in vacuo. The residue was purified by silica gel column chromatography (CHCl_3_/MeOH = 100/0 to 97/3) to afford compound **3b** (90 mg, 0.22 mmol, 77%) as a white form. ^1^H NMR (400 MHz, CDCl_3_): δ 7.92 (dd, *J* = 1.2 Hz, 8.4 Hz, 2H), 7.73 (s, 1H), 7.68–7.63 (m, 1H), 7.50 (t, *J* = 8.0 Hz, 2H), 5.78 (d, *J* = 2.9 Hz, 1H), 4.33 (dd, *J* = 5.8 Hz, 12.9 Hz, 1H), 4.10 (dd, *J* = 3.0 Hz, 5.2 Hz, 1H), 4.04 (dd, *J* = 2.3 Hz, 8.6 Hz, 2H), 3.90–3.84 (m, 1H), 3.79–3.73 (m, 1H), 3.57–3.51 (m, 1H), 2.70 (d, *J* = 7.3 Hz, 1H), 2.40 (br, 1H), 1.95 (d, *J* = 1.1 Hz, 3H), 1.66–1.57 (m, 2H), 0.92 (t, *J* = 7.4 Hz, 3H). ^13^C NMR (100 MHz, CDCl_3_): δ 168.75, 162.77, 149.30, 136.74, 135.16, 131.45, 130.50, 129.19, 110.91, 89.91, 84.82, 81.27, 72.82, 68.46, 61.18, 22.91, 12.62, 10.42. HRMS (MALDI): calcd for C_20_H_24_N_2_O_7_Na [M + Na]^+^ 427.1476, found 427.1473.

### 3.9. Synthesis of 3′-O-Acetyl-N^3^-benzoyl-5′-O-dimethoxytrityl-2′-O-propyl-5-methyluridine (***4b***)

Compound **3b** (80 mg, 0.20 mmol) was dissolved in dry pyridine (2.0 mL). DMTrCl (74 mg, 0.22 mmol) was added and the reaction mixture was stirred at room temperature for 4.5 h. DMAP (2 mg, 0.02 mmol) and Ac_2_O (93 µL, 1.0 mmol) were added and the reaction mixture was stirred at room temperature for 13 h. The resulting reaction mixture was concentrated in vacuo and diluted with ethyl acetate. The combined organic layers were washed with saturated NaHCO_3_ aq. and brine, dried over anhydrous Na_2_SO_4_, and concentrated in vacuo. The residue was purified by silica gel column chromatography (Hexane/Ethyl acetate = 3/1 to 2/1) to afford compound **4b** (104 mg, 0.14 mmol, 69%) as a white form. ^1^H NMR (400 MHz, CDCl_3_): δ 7.94 (dd, *J* = 1.1 Hz, 8.4 Hz, 2H), 7.72 (d, *J* = 1.2 Hz, 1H), 7.66–7.62 (m, 1H), 7.49 (t, *J* = 7.8 Hz, 2H), 7.41 (d, *J* = 7.1 Hz, 2H), 7.34–7.26 (m, 7H), 6.86 (dd, *J* = 2.0 Hz, 8.9 Hz, 4H), 6.04 (d, *J* = 4.4 Hz, 1H), 5.36 (t, *J* = 5.3 Hz, 1H), 4.31–4.28 (m, 2H), 3.81 (s, 6H), 3.65–3.59 (m, 2H), 3.51–3.46 (m, 1H), 3.39 (dd, *J* = 2.4 Hz, 11.0 Hz, 1H), 2.10 (s, 3H), 1.59–1.50 (m, 2H), 1.40 (d, *J* = 0.9 Hz, 3H), 0.89 (t, *J* = 7.4 Hz, 3H). ^13^C NMR (100 MHz, CDCl_3_): δ 170.13, 168.85, 162.78, 158.86, 158.84, 149.40, 144.07, 135.13, 135.00, 131.60, 130.54, 130.15, 129.11, 128.20, 128.10, 127.32, 113.37, 113.36, 111.40, 87.51, 87.24, 81.21, 80.37, 73.01, 70.62, 62.14, 55.29, 22.86, 20.70, 11.82, 10.41. HRMS (MALDI): calcd for C_43_H_44_N_2_O_10_Na [M + Na]^+^ 771.2888, found 771.2887.

### 3.10. Synthesis of 3′-O-Acetyl-N^3^-benzoyl-2′-O-propyl-5-methyluridine (***5b***)

Compound **4b** (80 mg, 0.11 mmol) was dissolved in CH_2_Cl_2_ (1.1 mL). TFA (0.3 mL) was added and the reaction mixture was stirred at room temperature for 30 min. The resulting reaction mixture was concentrated in vacuo and diluted with CHCl_3_. The combined organic layers were washed with saturated NaHCO_3_ aq. and brine, dried over anhydrous Na_2_SO_4_, and concentrated in vacuo. The residue was purified by silica gel column chromatography (Hexane/Ethyl acetate = 1/1 to 1/2) to afford compound **5b** (35 mg, 0.08 mmol, 72%) as a white form. ^1^H NMR (400 MHz, CDCl_3_): δ 7.92 (dd, *J* = 1.1 Hz, 8.4 Hz, 2H), 7.68–7.63 (m, 2H), 7.49 (t, *J* = 7.8 Hz, 2H), 5.71 (d, *J* = 4.8 Hz, 1H), 5.27 (t, *J* = 5.0 Hz, 1H), 4.35 (t, *J* = 5.0 Hz, 1H), 4.24–4.21 (m, 1H), 3.97 (dd, *J* = 1.8 Hz, 12.5 Hz, 1H), 3.76 (dd, *J* = 2.0 Hz, 12.5 Hz, 1H), 3.58–3.53 (m, 1H), 3.49–3.43 (m, 1H), 2.14 (s, 3H), 1.96 (d, *J* = 1.0 Hz, 3H), 1.58–1.49 (m, 2H), 0.88 (t, *J* = 7.4 Hz, 3H). ^13^C NMR (100 MHz, CDCl_3_): δ 170.49, 168.67, 162.74, 149.40, 137.24, 135.19, 131.42, 130.53, 129.20, 111.16, 90.89, 83.01, 79.36, 73.01, 70.43, 61.53, 22.88, 20.83, 12.66, 10.40. HRMS (MALDI): calcd for C_22_H_26_N_2_O_8_Na [M + Na]^+^ 469.1581, found 469.1580.

### 3.11. Synthesis of 2′-O-Propyl-5-methyluridine-5′-triphospahte (***6b***)

Compound **5b** was dissolved in dry pyridine and the solution was evaporated to dryness in vacuo. Compound **5b** (47 mg, 0.10 mmol) was dissolved in dry pyridine (0.5 mL) and 1,4-dioxane (0.5 mL). Then, 2-Chloro-4*H*-1,3,2-benzodioxaphosphorin-4-one (24 mg, 0.12 mmol) was added and the reaction mixture was stirred for 30 min at room temperature. Tris(tetrabutylammonium) hydrogen pyrophosphate (66 mg, 0.12 mmol) in DMF (1.0 mL) and tributylamine (86 µL, 0.36 mmol) were added and the reaction mixture was stirred for 1 h at room temperature. Next, 0.1 M I_2_ in pyridine/H_2_O (98:2, *v*/*v*) was added and the reaction mixture was stirred for 30 min at room temperature. Then, 10% Na_2_S_2_O_3_ aq. was added and the reaction mixture was stirred for 10 min at room temperature. The reaction mixture was purified by ethanol precipitation. The precipitate was dissolved in 7% NH_3_ aq. and the reaction mixture was stirred for 2 h at room temperature. The solvent was removed in vacuo and the residue was purified by HPLC to give compound **6b** (46 µmol, 46%). ^1^H NMR (400 MHz, D_2_O): δ7.77 (s, 1H), 6.01 (d, *J* = 5.2 Hz, 1H), 4.53–4.50 (m, 1H), 4.24 (d, *J* = 4.2 Hz, 3H), 4.17 (t, *J* = 5.3 Hz, 1H), 3.66–3.55 (m, 2H), 1.91 (s, 3H), 1.59–1.50 (m, 2H), 0.84 (t, *J* = 7.4 Hz, 3H). ^31^P NMR (120 MHz, D_2_O): δ −9.57 (d, *J* = 13.4 Hz), −12.66 (d, *J* = 14.6 Hz), −23.64 (t, *J* = 14.7 Hz). HRMS (MALDI): calcd for C_13_H_22_N_2_O_15_P_3_ [M – H]^−^ 539.0239, found 539.0243.

### 3.12. Synthesis of N^3^-Benzoyl-2′-O-butyl-3′,5′-O-(1,1,3,3-tetraisopropyldisiloxane-1,3-diyl)-5-methyluridine (***2c***)

Compound **1** (1.5 g, 2.5 mmol) was dissolved in dry toluene (7.5 mL). Ag_2_O (1460 mg, 6.3 mmol) and 1-iodobutane (7.1 mL, 63 mmol) were added and the reaction mixture was stirred at 80 °C for 15 h. The resulting reaction mixture was filtered by celite pad and the filtrate was diluted with CHCl_3_. The combined organic layers were washed with water and brine, dried over anhydrous Na_2_SO_4_, and concentrated in vacuo. The residue was purified by silica gel column chromatography (Hexane/Ethyl acetate = 85/15) to afford compound **2c** (303 mg, 0.46 mmol, 18%) as a white form. ^1^H NMR (400 MHz, CDCl_3_): δ 7.93 (dd, *J* = 1.2 Hz, 8.4 Hz, 2H), 7.73 (d, *J* = 1.2 Hz, 1H), 7.67–7.62 (m, 1H), 7.49 (t, *J* = 8.0 Hz, 2H), 5.70 (s, 1H), 4.29–4.21 (m, 2 H), 4.15 (dd, *J* = 2.1 Hz, 9.6 Hz, 1H), 4.00 (dd, *J* = 2.5 Hz, 13.6 Hz, 1H), 3.84 (d, *J* = 4.3 Hz, 1H), 3.75 (t, *J* = 6.3 Hz, 2H), 1.95 (d, *J* = 1.1 Hz, 3H), 1.58–1.50 (m, 2H), 1.42–1.33 (m, 2H), 1.13–1.03 (m, 28H), 0.87 (t, *J* = 7.4 Hz, 3H). ^13^C NMR (100 MHz, CDCl_3_): δ 169.14, 162.97, 148.96, 135.09, 134.87, 131.53, 130.55, 129.16, 110.05, 89.64, 82.19, 81.88, 71.24, 68.25, 59.38, 31.84, 19.15, 17.49, 17.42, 17.30, 17.28, 17.21, 17.09, 17.01, 16.92, 13.83, 13.58, 12.91, 12.81, 12.76, 12.64. HRMS (MALDI): calcd for C_33_H_52_N_2_O_8_NaSi_2_ [M + Na]^+^ 683.3154, found 683.3158.

### 3.13. Synthesis of N^3^-Benzoyl-2′-O-butyl-5-methyluridine (***3c***)

Compound **2c** (192 mg, 0.29 mmol) was dissolved in dry THF (3.0 mL). 1 M TBAF in THF (0.87 mL, 0.87 mmol) was added and the reaction mixture was stirred at room temperature for 30 min. The resulting reaction mixture was concentrated in vacuo. The residue was purified by silica gel column chromatography (CHCl_3_/MeOH = 100/0 to 97/3) to afford compound **3c** (95 mg, 0.23 mmol, 78%) as a white form. ^1^H NMR (400 MHz, CDCl_3_): δ 7.92 (dd, *J* = 1.2 Hz, 8.4 Hz, 2H), 7.73 (s, 1H), 7.68–7.63 (m, 1H), 7.50 (t, *J* = 7.8 Hz, 2H), 5.78 (d, *J* = 2.8 Hz, 1H), 4.33 (dd, *J* = 5.8 Hz, 12.8 Hz, 1H), 4.09 (dd, *J* = 3.0 Hz, 5.1 Hz, 1H), 4.04–4.02 (m, 2H), 3.89–3.84 (m, 1H), 3.83–3.77 (m, 1H), 3.60–3.55 (m, 1H), 2.69 (d, *J* = 7.3 Hz, 1H), 2.43 (br, 1H), 1.95 (d, *J* = 1.1 Hz, 3H), 1.61–1.54 (m, 2H), 1.41–1.32 (m, 2H), 0.91 (t, *J* = 7.4 Hz, 3H). ^13^C NMR (100 MHz, CDCl_3_): δ 168.74, 162.77, 149.27, 136.73, 135.15, 131.43, 130.49, 129.17, 110.87, 89.81, 84.80, 81.31, 71.02, 68.43, 61.14, 31.69, 19.16, 13.81, 12.60. HRMS (MALDI): calcd for C_21_H_26_N_2_O_7_Na [M + Na]^+^ 441.1632, found 441.1633.

### 3.14. Synthesis of 3′-O-Acetyl-N^3^-benzoyl-2′-O-butyl-5′-O-dimethoxytrityl-5-methyluridine (***4c***)

Compound **3c** (85 mg, 0.20 mmol) was dissolved in dry pyridine (2.0 mL). DMTrCl (74 mg, 0.22 mmol) was added and the reaction mixture was stirred at room temperature for 4.5 h. DMAP (2 mg, 0.02 mmol) and Ac_2_O (93 µL, 1.0 mmol) were added and the reaction mixture was stirred at room temperature for 13 h. The resulting reaction mixture was concentrated in vacuo and diluted with ethyl acetate. The combined organic layers were washed with saturated NaHCO_3_ aq. and brine, dried over anhydrous Na_2_SO_4_, and concentrated in vacuo. The residue was purified by silica gel column chromatography (Hexane/Ethyl acetate = 3/1 to 2/1) to afford compound **4c** (113 mg, 0.15 mmol, 75%) as a white form. ^1^H NMR (400 MHz, CDCl_3_): δ 7.94 (d, *J* = 7.2 Hz, 2H), 7.71 (d, *J* = 1.0 Hz, 1H), 7.64 (t, *J* = 7.4 Hz, 1H), 7.49 (t, *J* = 7.8 Hz, 2H), 7.41 (d, *J* = 7.2 Hz, 2H), 7.32–7.26 (m, 7H), 6.86 (dd, *J* = 2.0 Hz, 8.8 Hz, 4H), 6.04 (d, *J* = 4.5 Hz, 1H), 5.36 (t, *J* = 5.2 Hz, 1H), 4.30–4.27 (m, 2H), 3.80 (s, 6H), 3.69–3.60 (m, 2H), 3.56–3.47 (m, 1H), 3.39 (dd, *J* = 2.2 Hz, 11.0 Hz, 1H), 2.09 (s, 3H), 1.57–1.49 (m, 2H), 1.40 (s, 3H), 1.38–1.31 (m, 2H), 0.89 (t, *J* = 7.4 Hz, 3H). ^13^C NMR (100 MHz, CDCl_3_): δ 170.13, 168.86, 162.79, 158.86, 158.85, 149.40, 144.08, 135.14, 135.03, 135.01, 131.63, 130.54, 130.16, 129.12, 128.20, 128.10, 127.33, 113.38, 113.37, 114.40, 87.46, 87.25, 81.22, 80.35, 73.02, 71.15, 70.63, 62.16, 55.30, 31.65, 22.87, 20.70, 19.10, 13.83, 11.82, 10.42. HRMS (MALDI): calcd for C_44_H_46_N_2_O_10_Na [M + Na]^+^ 785.3045, found 785.3035.

### 3.15. Synthesis of 3′-O-Acetyl-N^3^-benzoyl-2′-O-butyl-5-methyluridine (***5c***)

Compound **4c** (120 mg, 0.16 mmol) was dissolved in CH_2_Cl_2_ (1.6 mL). TFA (0.4 mL) was added and the reaction mixture was stirred at room temperature for 30 min. The resulting reaction mixture was concentrated in vacuo and diluted with CHCl_3_. The combined organic layers were washed with saturated NaHCO_3_ aq. and brine, dried over anhydrous Na_2_SO_4_, and concentrated in vacuo. The residue was purified by silica gel column chromatography (Hexane/Ethyl acetate = 1/1 to 1/2) to afford compound **5c** (57 mg, 0.12 mmol, 75%) as a white form. ^1^H NMR (400 MHz, CDCl_3_): δ 7.92 (dd, *J* = 1.2 Hz, 8.4 Hz, 2H), 7.68–7.63 (m, 2H), 7.49 (t, *J* = 7.8 Hz, 2H), 5.71 (d, *J* = 4.8 Hz, 1H), 5.26 (t, *J* = 5.0 Hz, 1H), 4.33 (t, *J* = 5.0 Hz, 1H), 4.23–4.21 (m, 1H), 3.96 (dd, *J* = 1.8 Hz, 12.5 Hz, 1H), 3.76 (dd, *J* = 2.0 Hz, 12.5 Hz, 1H), 3.62–3.56 (m, 1H), 3.53–3.47 (m, 1H), 2.14 (s, 3H), 1.96 (d, *J* = 1.1 Hz, 3H), 1.53–1.46 (m, 2H), 1.37–1.29 (m, 2H), 0.88 (t, *J* = 7.4 Hz, 3H). ^13^C NMR (100 MHz, CDCl_3_): δ 170.47, 168.67, 162.74, 149.39, 137.19, 135.17, 131.43, 130.51, 129.19, 111.14, 90.79, 82.99, 79.44, 71.20, 70.42, 61.50, 31.68, 20.81, 19.10, 13.84, 12.64. HRMS (MALDI): calcd for C_23_H_28_N_2_O_8_Na [M + Na]^+^ 483.1738, found 483.0741.

### 3.16. Synthesis of 2′-O-Butyl-5-methyluridine-5′-triphospahte (***6c***)

Compound **5c** was dissolved in dry pyridine and the solution was evaporated to dryness in vacuo. Compound **5c** (30 mg, 0.065 mmol) was dissolved in dry pyridine (0.25 mL) and 1,4-dioxane (0.25 mL). Then, 2-Chloro-4*H*-1,3,2-benzodioxaphosphorin-4-one (15 mg, 0.072 mmol) was added and the reaction mixture was stirred for 30 min at room temperature. Tris(tetrabutylammonium) hydrogen pyrophosphate (43 mg, 0.078 mmol) in DMF (0.5 mL) and tributylamine (52 µL, 0.22 mmol) were added and the reaction mixture was stirred for 1 h at room temperature. Next, 0.1 M I_2_ in pyridine/H_2_O (98:2, *v*/*v*) was added and the reaction mixture was stirred for 30 min at 0 °C. Then, 10% Na_2_S_2_O_3_ aq. was added and the reaction mixture was stirred for 10 min at room temperature. The reaction mixture was purified by ethanol precipitation. The precipitate was dissolved in 7% NH_3_ aq. and the reaction mixture was stirred for 2 h at room temperature. The solvent was removed in vacuo and the residue was purified by HPLC to give compound **6c** (6 µmol, 10%). ^1^H NMR (400 MHz, D_2_O): δ7.79 (s, 1H), 6.03 (d, *J* = 5.4 Hz, 1H), 4.52 (t, *J* = 4.5 Hz, 1H), 4.27–4.24 (m, 3H), 4.18 (t, *J* = 5.4 Hz, 1H), 3.73–3.61 (m, 2H), 1.93 (s, 3H), 1.56–1.49 (m, 2H), 1.34–1.25 (m, 2H), 0.84 (t, *J* = 7.4 Hz, 3H). ^31^P NMR (120 MHz, D_2_O): δ −9.80 (d, *J* = 12.7 Hz), −12.65 (d, *J* = 14.5 Hz), −23.69 (t, *J* = 14.7 Hz). HRMS (MALDI): calcd for C_14_H_24_N_2_O_15_P_3_ [M – H]^−^ 553.0395, found 553.0424.

### 3.17. Synthesis of N^3^-Benzoyl-2′-O-isopropyl-3′,5′-O-(1,1,3,3-tetraisopropyldisiloxane-1,3-diyl)-5-methyluridine (***2d***)

Compound **1** (1.5 g, 2.5 mmol) was dissolved in dry toluene (7.5 mL). Ag_2_O (1460 mg, 6.3 mmol) and 2-iodopropane (6.2 mL, 63 mmol) were added and the reaction mixture was stirred at 80 °C for 13 h. The resulting reaction mixture was filtered by celite pad and the filtrate was diluted with CHCl_3_. The combined organic layers were washed with water and brine, dried over anhydrous Na_2_SO_4_, and concentrated in vacuo. The residue was purified by silica gel column chromatography (Hexane/Ethyl acetate = 85/15) to afford compound **2d** (372 mg, 0.58 mmol, 23%) as a white form. ^1^H NMR (400 MHz, CDCl_3_): δ 7.93 (dd, *J* = 1.2 Hz, 8.4 Hz, 2H), 7.76 (d, *J* = 1.2 Hz, 1H), 7.67–7.62 (m, 1H), 7.49 (t, *J* = 7.8 Hz, 2H), 5.64 (s, 1H), 4.27 (d, *J* = 13.7 Hz, 1H), 4.21–4.14 (m, 2 H), 4.01–3.95 (m, 2 H), 3.93 (d, *J* = 4.1 Hz, 1H), 1.95 (d, *J* = 1.2 Hz, 3H), 1.19–1.03 (m, 34H). ^13^C NMR (100 MHz, CDCl_3_): δ 169.19, 163.03, 149.01, 135.11, 134.93, 131.51, 130.59, 129.16, 109.91, 90.44, 82.01, 80.35, 72.83, 67.88, 59.41, 22.59, 22.35, 17.50, 17.42, 17.29, 17.21, 17.12, 17.05, 16.95, 13.58, 12.91, 12.83, 12.77, 12.73. HRMS (MALDI): calcd for C_32_H_50_N_2_O_8_NaSi_2_ [M + Na]^+^ 669.2998, found 669.2994.

### 3.18. Synthesis of N^3^-Benzoyl-2′-O-isopropyl-5-methyluridine (***3d***)

Compound **2d** (277 mg, 0.43 mmol) was dissolved in dry THF (4.3 mL). 1 M TBAF in THF (1.3 mL, 1.3 mmol) was added and the reaction mixture was stirred at room temperature for 30 min. The resulting reaction mixture was concentrated in vacuo. The residue was purified by silica gel column chromatography (CHCl_3_/MeOH = 99/1 to 97/3) to afford compound **3d** (150 mg, 0.37 mmol, 86%) as a white form. ^1^H NMR (400 MHz, CDCl_3_): δ 7.91 (dd, *J* = 1.1 Hz, 8.4 Hz, 2H), 7.67–7.64 (m, 2H), 7.49 (t, *J* = 7.8 Hz, 2H), 5.68 (d, *J* = 2.9 Hz, 1H), 4.29–4.24 (m, 2H), 4.06–3.91 (m, 3H), 3.85–3.81 (m, 1H), 2.72 (d, *J* = 6.0 Hz, 1H), 2.59 (br, 1H), 1.95 (d, *J* = 1.1 Hz, 3H), 1.19 (dd, *J* = 3.9 Hz, 6.1 Hz, 6H). ^13^C NMR (100 MHz, CDCl_3_): δ 168.67, 162.75, 149.38, 137.23, 135.16, 131.42, 130.47, 129.17, 110.91, 91.17, 85.14, 78.75, 72.80, 68.66, 61.43, 22.89, 22.07, 12.58. HRMS (MALDI): calcd for C_20_H_24_N_2_O_7_Na [M + Na]^+^ 427.1476, found 427.1472.

### 3.19. Synthesis of 3′-O-Acetyl-N^3^-benzoyl-5′-O-dimethoxytrityl-2′-O-isopropyl-5-methyluridine (***4d***)

Compound **3d** (150 mg. 0.37 mmol) was dissolved in dry pyridine (4.0 mL). DMTrCl (139 mg, 0.41 mmol) was added and the reaction mixture was stirred at room temperature for 6 h. DMAP (5 mg, 0.04 mmol) and Ac_2_O (173 µL, 1.9 mmol) were added and the reaction mixture was stirred at room temperature for 18 h. The resulting reaction mixture was concentrated in vacuo and diluted with ethyl acetate. The combined organic layers were washed with saturated NaHCO_3_ aq. and brine, dried over anhydrous Na_2_SO_4_, and concentrated in vacuo. The residue was purified by silica gel column chromatography (Hexane/Ethyl acetate = 3/1 to 2/1) to afford compound **4d** (204 mg, 0.27 mmol, 74%) as a white form. ^1^H NMR (400 MHz, CDCl_3_): δ 7.93 (dd, *J* = 1.2 Hz, 8.4 Hz, 2H), 7.74 (d, *J* = 1.2 Hz, 1H), 7.66–7.62 (m, 1H), 7.49 (t, *J* = 7.8 Hz, 2H), 7.41 (dd, *J* = 1.3 Hz, 8.3 Hz, 2H), 7.34–7.26 (m, 7H), 6.86 (dd, *J* = 2.1 Hz, 8.9 Hz, 4H), 6.00 (d, *J* = 5.0 Hz, 1H), 5.37 (t, *J* = 5.1 Hz, 1H), 4.38 (t, *J* = 5.1 Hz, 1H), 4.29–4.27 (m, 1H), 3.80 (d, *J* = 0.8 Hz, 6H), 3.60 (dd, *J* = 2.3 Hz, 11.0 Hz, 1H), 3.38 (dd, *J* = 2.3 Hz, 10.9 Hz, 1H), 2.10 (s, 3H), 1.38 (d, *J* = 1.0 Hz, 3H), 1.15 (d, *J* = 6.0 Hz, 3H), 1.09 (d, *J* = 6.0 Hz, 3H). ^13^C NMR (100 MHz, CDCl_3_): δ 170.12, 168.80, 162.77, 158.87, 158.85, 149.46, 144.07, 135.10, 135.08, 134.97, 131.61, 130.50, 130.15, 129.10, 128.18, 128.10, 127.34, 113.37, 113.36, 111.41, 87.74, 87.27, 81.27, 78.07, 72.44, 70.87, 62.42, 55.29, 22.33, 22.06, 20.73, 11.73. HRMS (MALDI): calcd for C_43_H_44_N_2_O_10_Na [M + Na]^+^ 771.2888, found 771.2894.

### 3.20. Synthesis of 3′-O-Acetyl-N^3^-benzoyl-2′-O-isopropyl-5-methyluridine (***5d***)

Compound **4d** (167 mg, 0.22 mmol) was dissolved in CH_2_Cl_2_ (2.2 mL). TFA (0.6 mL) was added and the reaction mixture was stirred at room temperature for 30 min. The resulting reaction mixture was concentrated in vacuo and diluted with CHCl_3_. The combined organic layers were washed with saturated NaHCO_3_ aq. and brine, dried over anhydrous Na_2_SO_4_, and concentrated in vacuo. The residue was purified by silica gel column chromatography (Hexane/Ethyl acetate = 1/1 to 1/2) to afford compound **5d** (67 mg, 0.15 mmol, 68%) as a white form. ^1^H NMR (400 MHz, CDCl_3_): δ 7.91 (dd, *J* = 1.1 Hz, 8.4 Hz, 2H), 7.67–7.63 (m, 1H), 7.56 (d, *J* = 1.0 Hz, 1H), 7.49 (t, *J* = 7.8 Hz, 2H), 5.57 (d, *J* = 5.4 Hz, 1H), 5.27 (t, *J* = 4.8 Hz, 1H), 4.49 (t, *J* = 5.3 Hz, 1H), 4.23–4.21 (m, 1H), 3.94 (dd, *J* = 1.9 Hz, 12.5 Hz, 1H), 3.79–3.70 (m, 2H), 2.14 (s, 3H), 1.96 (d, *J* = 1.0 Hz, 3H), 1.10 (dd, *J* = 6.0 Hz, 11.0 Hz, 6H). ^13^C NMR (100 MHz, CDCl_3_): δ 170.36, 168.55, 162.71, 149.45, 137.83, 135.19, 131.38, 130.50, 129.18, 111.11, 92.20, 83.21, 72.60, 70.70, 61.75, 22.24, 20.84, 12.60. HRMS (MALDI): calcd for C_22_H2_6_N_2_O_8_Na [M + Na]^+^ 469.1581, found 469.1583.

### 3.21. Synthesis of 2′-O-Isopropyl-5-methyluridine-5′-triphospahte (***6d***)

Compound **5d** was dissolved in dry pyridine and the solution was evaporated to dryness in vacuo. Compound **5d** (47 mg, 0.10 mmol) was dissolved in dry pyridine (0.5 mL) and 1,4-dioxane (0.5 mL). Then, 2-Chloro-4*H*-1,3,2-benzodioxaphosphorin-4-one (22 mg, 0.11 mmol) was added and the reaction mixture was stirred for 30 min at room temperature. Tris(tetrabutylammonium) hydrogen pyrophosphate (66 mg, 0.12 mmol) in DMF (1.0 mL) and tributylamine (79 µL, 0.33 mmol) were added and the reaction mixture was stirred for 1 h at room temperature. Next, 0.1 M I_2_ in pyridine/H_2_O (98:2, *v*/*v*) was added and the reaction mixture was stirred for 30 min at 0 °C. Then, 10% Na_2_S_2_O_3_ aq. was added and the reaction mixture was stirred for 10 min at room temperature. The reaction mixture was purified by ethanol precipitation. The precipitate was dissolved in 7% NH_3_ aq. and the reaction mixture was stirred for 2 h at room temperature. The solvent was removed in vacuo and the residue was purified by HPLC to give compound **6d** (16 µmol, 16%). ^1^H NMR (400 MHz, D_2_O): δ7.79 (s, 1H), 6.01 (d, *J* = 5.4 Hz, 1H), 4.57 (t, *J* = 4.5 Hz, 1H), 4.30–4.27 (m, 4H), 3.96–3.90 (m, 1H), 1.96 (s, 3H), 1.20 (d, *J* = 6.2 Hz, 3H), 1.15 (d, *J* = 6.2 Hz, 3H). ^31^P NMR (120 MHz, D_2_O): δ − 8.71 (d, *J* = 14.4 Hz), −12.22 (d, *J* = 13.8 Hz), −22.79 (t, *J* = 14.1 Hz). HRMS (MALDI): calcd for C_13_H_22_N_2_O_15_P_3_ [M − H]^−^ 539.0239, found 539.0236.

### 3.22. Synthesis of 2,2′-Anhydro-5′-O-(tert-butyldimethylsilyl)-5-methyluridine (***8***)

Compound **7** (500 mg, 2.1 mmol) was dissolved in dry DMF (2.0 mL). Imidazole (357 mg, 5.3 mmol) and TBSCl (362 mg, 2.4 mmol) were added and the reaction mixture was stirred at room temperature for 5 h. The resulting reaction mixture was concentrated in vacuo and diluted with ethyl acetate. The combined organic layers were washed with saturated NaHCO_3_ aq. and brine, dried over anhydrous Na_2_SO_4_, and concentrated in vacuo. The residue was purified by silica gel column chromatography (CHCl_3_/MeOH = 99/1 to 95/5) to afford compound **8** (556 mg, 1.6 mmol, 76%) as a white form. ^1^H NMR (400 MHz, DMSO-*d*_6_): δ7.77 (d, *J* = 1.2 Hz, 1H), 6.28 (d, *J* = 5.7 Hz, 1H), 5.94 (d, *J* = 4.4 Hz, 1H), 5.19 (d, *J* = 7.7Hz, 1H), 4.32 (br, 1H), 4.09–4.05 (m, 1H), 3.47–3.37 (m, 2H), 1.78 (d, *J* = 1.1Hz, 3H), 0.78 (s, 9H), −0.06 (s, 3H), −0.08 (s, 3H). ^13^C NMR (100 MHz, DMSO-*d*_6_): δ 171.52, 159.14, 132.25, 116.90, 89.97, 88.37, 88.05, 74.22, 62.29, 25.69, 18.00, 13.50, −5.52. HRMS (MALDI): calcd for C_16_H_26_N_2_O_5_NaSi [M + Na]^+^ 377.1503, found 377.1506.

### 3.23. Synthesis of 2′-O-Acetoxylethyl-3′-O-acetyl-5′-O-(tert-butyldimethylsilyl)-5-methyluridine (***9***)

Ethylene glycol (5.0 mL) was added to 0.96 M Boran in THF (2.6 mL, 2.5 mmol). Compound **8** (400 mg. 1.0 mmol) and NaHCO_3_ (1 mg) were added and the reaction mixture was stirred at 150 °C for 24 h. The resulting reaction mixture was diluted with CHCl_3_. The combined organic layers were washed with water and brine, dried over anhydrous Na_2_SO_4_, and concentrated in vacuo. The residue was dissolved in dry pyridine (10 mL). DMAP (12 mg, 0.1 mmol) and Ac_2_O (930 µL, 10 mmol) were added and the reaction mixture was stirred at room temperature for 16 h. The resulting reaction mixture was concentrated in vacuo and diluted with ethyl acetate. The combined organic layers were washed with saturated NaHCO_3_ aq. and brine, dried over anhydrous Na_2_SO_4_, and concentrated in vacuo. The residue was purified by silica gel column chromatography (Hexane/Ethyl acetate = 1/1) to afford compound **9** (250 mg, 0.50 mmol, 50%) as a white form. ^1^H NMR (400 MHz, CDCl_3_): δ 8.85 (s, 1H), 7.48 (d, *J* = 1.0 Hz, 1H), 6.06 (d, *J* = 5.2 Hz, 1H), 5.13 (t, *J* = 4.8 Hz, 1H), 4.22–4.21 (m, 1H), 4.14 (t, *J* = 4.3 Hz, 2H), 4.08 (t, *J* = 5.4 Hz, 1H), 3.98 (dd, *J* = 1.7 Hz, 11.8 Hz, 1H), 3.82–3.70 (m, 3H), 2.14 (s, 3H), 2.03 (s, 3H),1.92 (d, *J* = 0.9 Hz, 3H), 0.94 (s, 9H), 0.13 (s, 6H). ^13^C NMR (100 MHz, CDCl_3_): δ 170.88, 170.25, 163.59, 150.37, 134.84, 111.30, 86.64, 82.40, 80.96, 70.22, 69.15, 63.15, 62.34, 25.98, 20.85, 20.77, 18.47, 12.60, −5.32, −5.40. HRMS (MALDI): calcd for C_22_H_36_N_2_O_9_NaSi [M + Na]^+^ 523.2082, found 523.2082.

### 3.24. Synthesis of 2′-O-Acetoxylethyl-3′-O-acetyl-5-methyluridine (***10***)

Compound **9** (250 mg, 0.50 mmol) was dissolved in dry THF (5.0 mL). 1M TBAF in THF (1.5 mL, 1.5 mmol) was added and the reaction mixture was stirred at room temperature for 30 min. The resulting reaction mixture was concentrated in vacuo. The residue was purified by silica gel column chromatography (Hexane/Ethyl acetate = 1/9) to afford compound **10** (170 mg, 0.44 mmol, 88%) as a white form. ^1^H NMR (400 MHz, CDCl_3_): δ 9.12 (s, 1H), 7.41 (d, *J* = 1.2 Hz, 1H), 5.62 (d, *J* = 5.5 Hz, 1H), 5.32 (dd, *J* = 4.1 Hz, 5.2 Hz, 1H), 4.49 (t, *J* = 5.4 Hz, 1H), 4.24–4.22 (m, 1H), 4.20–4.11 (m, 2H), 3.95 (dd, *J* = 1.9 Hz, 12.5 Hz, 1H), 3.81–3.73 (m, 3H), 3.25 (br, 1H), 2.15 (s, 3H), 2.03 (s, 3H), 1.92 (d, *J* = 1.1 Hz, 3H). ^13^C NMR (100 MHz, CDCl_3_): δ 170.90, 170.35, 163.74, 150.45, 137.94, 111.27, 91.21, 83.23, 79.28, 70.67, 69.19, 63.24, 61.78, 20.86, 20.82, 12.51. HRMS (MALDI): calcd for C_16_H_22_N_2_O_9_Na [M + Na]^+^ 409.1218, found 409.1219.

### 3.25. Synthesis of 2′-O-Hydroxylethyl-5-methyluridine-5′-triphospahte (***11***)

Compound **10** was dissolved in dry pyridine and the solution was evaporated to dryness in vacuo. Compound **10** (39 mg, 0.10 mmol) was dissolved in dry pyridine (0.5 mL) and 1,4-dioxane (0.5 mL). Then, 2-Chloro-4*H*-1,3,2-benzodioxaphosphorin-4-one (22 mg, 0.11 mmol) was added and the reaction mixture was stirred for 30 min at room temperature. Tris(tetrabutylammonium) hydrogen pyrophosphate (66 mg, 0.12 mmol) in DMF (1.0 mL) and tributylamine (133 µL, 0.56 mmol) were added and the reaction mixture was stirred for 1 h at room temperature. Next, 0.1 M I_2_ in pyridine/H_2_O (98:2, *v*/*v*) was added and the reaction mixture was stirred for 30 min at 0 °C. Then, 10% Na_2_S_2_O_3_ aq. was added and the reaction mixture was stirred for 10 min at room temperature. The reaction mixture was purified by ethanol precipitation. The precipitate was dissolved in 7% NH_3_ aq. And the reaction mixture was stirred for 2 h at room temperature. The solvent was removed in vacuo and the residue was purified by HPLC to give compound **11** (16 µmol, 16%). ^1^H NMR (400 MHz, D_2_O): δ7.80 (s, 1H), 6.08 (d, *J* = 4.7 Hz, 1H), 4.56 (d, *J* = 4.9 Hz, 1H), 4.31–4.24 (m, 4H), 3.85–3.75 (m, 4H), 1.96 (s, 3H). ^31^P NMR (120 MHz, D_2_O): δ −9.49 (d, *J* = 11.7 Hz), −11.84 (d, *J* = 14.2 Hz), −22.72 (s). HRMS (MALDI): calcd for C_12_H_20_N_2_O_16_P_3_ [M − H]^−^ 541.0031, found 541.0044.

### 3.26. Synthesis of 2′-O-Methoxyethyl-5-methyluridine-5′-triphospahte (***13***)

Compound **12** [36] was dissolved in dry pyridine and the solution was evaporated to dryness in vacuo. Compound **12** (100 mg, 0.28 mmol) was dissolved in dry pyridine (1 mL) and 1,4-dioxane (2.0 mL). Then 2-Chloro-4*H*-1,3,2-benzodioxaphosphorin-4-one (69 mg, 0.34 mmol) was added and the reaction mixture was stirred for 1 h at room temperature. Tris(tetrabutylammonium) hydrogen pyrophosphate (307 mg, 0.56 mmol) in DMF (1.0 mL) and tributylamine (133 µL, 0.56 mmol) were added and the reaction mixture was stirred for 1.5 h at room temperature. Next, 0.1 M I_2_ in pyridine/H_2_O (98:2, *v*/*v*) was added and the reaction mixture was stirred for 1 h at room temperature. Then, 10% Na_2_S_2_O_3_ aq. was added and the reaction mixture was stirred for 10 min at room temperature. The reaction mixture was purified by ethanol precipitation. The precipitate was dissolved in 7% NH_3_ aq. and the reaction mixture was stirred for 1 h at room temperature. The solvent was removed in vacuo and the residue was purified by HPLC to give compound **13** (44 µmol, 16%). ^1^H NMR (400 MHz, D_2_O): δ7.79 (s, 1H), 6.06 (d, *J* = 5.0 Hz, 1H), 4.55 (t, *J* = 4.8 Hz, 1H), 4.32–4.23 (m, 4H), 3.87–3.85 (m, 2H), 3.66–3.64 (m, 2H), 3.37 (s, 3H), 1.96 (s, 3H). ^31^P NMR (120 MHz, D_2_O): δ −9.12(d, *J* = 13.7 Hz), −11.96 (d, *J* = 14.5 Hz), −22.69 (t, *J* = 14.0 Hz). HRMS (MALDI): calcd for C_13_H_22_N_2_O_15_P_3_ [M + Na]^+^ 579.0153, found 579.0144.

### 3.27. Expression and Purification of KOD DGLNK

KOD DGLNK was expressed and purified according to a previous report [23].

### 3.28. Polymerase Incorporation of 2′-O-Alkyl-5-methyluridine Triphosphates into Oligonucleotide

The reaction mixture [1 × KOD Dash^®^ buffer (TOYOBO, Osaka, Japan), DNA or 2′-OMe primer (Primer**1** or Primer**2**: 0.4 µM), DNA template (Template**1**: 0.5 µM), dTTP or 2′-*O*-alkyl-^5m^UTPs (0.2 mM), and MnSO_4_ (0 or 0.1 or 0.5 or 1.0 mM)] was prepared. Then, the reaction mixture was denatured at 94 °C for 1 min and annealed at 25 °C for 1 h (1.2 °C/min), KOD DGLNK (50 or 150 ng/µL) was added and incubated at 74 °C for 30 or 60 min. The reaction was stopped by stop buffer (aqueous 3 mM EDTA containing 0.1% bromophenol blue and aqueous 7 M Urea) and heated for 5 min at 95 °C. The reaction was analyzed by using a 20% denaturing urea-PAGE (70 min, 55 °C).

### 3.29. Enzymatic Synthesis of 2′-O-Alkyl-modified Oligonucleotide Libraries

The reaction mixture [1× KOD Dash^®^ buffer (TOYOBO, Osaka, Japan), 2′-OMe primer (Primer**3**: 0.4 µM), DNA template (Template**2**: 0.5 µM), dTTP or 2′-*O*-alkyl-^5m^UTPs (0.2 mM), 2′-OMe-ATP (0.2 mM), 2′-OMe-GTP (0.2 mM), 2′-OMe-CTP (0.2 mM), MnSO_4_ (1.0 or 3.0 or 5.0 mM) and KOD DGLNK (300 ng/µL)] was prepared. Then, the reaction mixture was denatured at 94 °C for 1 min and annealed at 25 °C for 1 h (1.2 °C/min), KOD DGLNK was added and incubated from 60 °C for 1 h, 60 °C to 74 °C for 4 or 8 h, and 74 °C for 1 h. After polymerization, DNase1 (95 mU/µL) was added and incubated at 37 °C for 30 min. The reaction was stopped by stop buffer (aqueous 3 mM EDTA containing 0.1% bromophenol blue and aqueous 7 M Urea) and heated for 5 min at 95 °C. The reaction was analyzed by using a 10% denaturing urea-PAGE (32 min, 55 °C).

### 3.30. Preparation of 2′-O-ALKYL-modified Oligonucleotide Libraries for Analysis of Properties

The reaction mixture [1× KOD Dash^®^ buffer (TOYOBO, Osaka, Japan), 2′-OMe primer (Primer**3**: 0.4 µM), DNA template (Template**2**: 0.5 µM), 2′-*O*-alkyl-^5m^UTPs (0.2 mM), 2′-OMe-ATP (0.2 mM), 2′-OMe-GTP (0.2 mM), 2′-OMe-CTP (0.2 mM), MnSO_4_ (1.0 or 3.0 mM) and KOD DGLNK (300 ng/µL)] was prepared. Then, the reaction mixture was denatured at 94 °C for 1 min and annealed at 25 °C for 1 h (1.2 °C/min), KOD DGLNK (300 ng/µL) was added and incubated from 60 °C for 1 h, 60 °C to 74 °C for 4 or 8 h, and 74 °C for 1 h. After polymerization, DNase1 (95 mU/µL) was added and incubated at 37 °C for 30 min. Then, Proteinase K (36 mU/µL) was added and incubated at 37 °C for 30 min. The reaction was stopped by stop buffer (0.1% bromophenol blue and aqueous 7 M Urea) and heated for 5 min at 95 °C. The oligonucleotides were purified by using a 10% denaturing urea-PAGE (120 min, 55 °C).

### 3.31. Enzymatic Stability of Oligonucleotide Libraries

The reaction mixture [oligonucleotide libraries (0.1 µM) and 50% FBS] was prepared. Then, the reaction mixture was incubated at 37 °C. After incubation, the reaction mixture was heated at 94 °C for 5 min. Then, Proteinase K (36 mU/µL) was added and incubated at 37 °C for 30 min. The reaction was stopped by stop buffer (aqueous 3 mM EDTA containing 0.1% bromophenol blue and aqueous 7 M Urea). After adding Cy5-labeled DNA (83 nM) as an internal standard to the reaction mixtures, they were heated for 5 min at 95 °C. The reaction was analyzed by using a 10% denaturing urea-PAGE (30 min, 55 °C).

### 3.32. Ion Pair RP-HPLC Analysis of Oligonucleotide Libraries

Oligonucleotide libraries (5.0 pmol) were injected onto a XBridge Oligonucleotide BEH C18 column. The column temperature was 50 °C, with a flow rate of 1.0 mL/min, and detection at 260 nm. Mobile phase A consisted of 100 mM triethylamine acetic acid solution, pH 6.9, and mobile phase B was acetonitrile. The column was initially maintained at 10% mobile phase B, and then at a gradient of 10% to 40% over 15 min.

### 3.33. Ion Pair RP-HPLC Analysis of Nucleotide Triphosphates

Nucleotide triphosphates (700 pmol) were injected onto a XBridge Oligonucleotide BEH C18 column. The column temperature was 50 °C, with a flow rate of 1.0 mL/min, and detection at 260 nm. Mobile phase A consisted of 100 mM triethylamine acetic acid solution (pH 6.9), and mobile phase B was acetonitrile. The column was initially maintained at 5% mobile phase B, and then at a gradient of 5% to 20% over 15 min.

## 4. Conclusions

In conclusion, 2′-OEt, 2′-OPr, 2′-OBu, 2′-O*^i^*Pr, and 2′-HE-^5m^UTPs were newly synthesized and incorporated them into oligonucleotides by KOD DGLNK. By mixing the 2′-OMe modification, oligonucleotide libraries were enzymatically synthesized. These 2′-*O*-alkyl-modified oligonucleotides showed various hydrophobicities. Furthermore, these 2′-*O*-alkyl-modified oligonucleotide libraries were more stable than unmodified DNA. Our results can accelerate the development of aptamers or nucleic acid enzymes for therapeutic use.

## Data Availability

Data are contained within the article and supplementary materials.

## Supplementary Materials

The following supporting information can be downloaded at: https://www.mdpi.com/article/10.3390/molecules28237911/s1, Table S1: Sequences of the primers and templates that were used in this study; Scheme S1: Synthesis of 2′-OEt-^5m^UTP (**6a**), 2′-OPr-^5m^UTP (**6b**), 2′-OBu-^5m^UTP (**6c**), and 2′-O*^i^*Pr-^5m^UTP (**6d**); Scheme S2: Synthesis of 2′-HE-^5m^UTP (**11**); Scheme S3: Synthesis of 2′-MOE-^5m^UTP (**13**); Figure S1: Polymerase incorporation of 2′-*O*-alkyl-^5m^UTPs with a DNA primer; Figure S2: Polymerase incorporation of 2′-*O*-alkyl-^5m^UTPs with DNA primer at different Mn^2+^ concentrations; Figure S3: Polymerase incorporation of 2′-*O*-alkyl-^5m^UTPs with a 2′-OMe-modified primer at different Mn^2+^ concentrations; Figure S4: Enzymatic synthesis of ON_*^i^*Pr, ON_HE, and ON_MOE at different Mn^2+^ concentrations and incubation times; Figure S5: Ion pair RP-HPLC analysis of dTTP and 2′-*O*-alkyl-^5m^UTPs; Figures S5–S29: NMR spectra of the all prepared compounds.
