# Supplementary material for: Systematic Analysis of 2′-O-Alkyl Modified Analogs for Enzymatic Synthesis and Their Oligonucleotide Properties
Source: Molecules. 2023 Dec 2;28(23):7911. doi: 10.3390/molecules28237911 (PMC10708256; doi:10.3390/molecules28237911)
Supplement: Supplementary file 1 [file molecules-28-07911-s001.zip › molecules-2718188-supplementary.pdf]

Supporting Information

Systematic Analysis of 2′-*O*-Alkyl Modified Analogs for Enzymatic Synthesis and Their Oligonucleotide Properties

Kenta Ishida [1,2], Yuuya Kasahara [1,2,*], Hidekazu Hoshino [1], Takumi Okuda [1,2] and Satoshi Obika [1,2,*]

[1] National Institutes of Biomedical Innovation, Health and Nutrition (NIBIOHN), 7-6-8 Saito-Asagi, Ibaraki, Osaka 567-0085, Japan.
[2] Graduate School of Pharmaceutical Sciences, Osaka University, 1-6 Yamadaoka, Suita, Osaka 565-0871, Japan
* Correspondence: y-kasahara@nibiohn.go.jp (Y.K.); obika@phs.osaka-u.ac.jp (S.O.); Tel.: +81-72-641-9882 (Y.K.); +81-6-6879-8200 (S.O.)

Contents:

・Table S1. Sequences of primers and templates that were used in this study.

・Scheme S1. Synthesis of 2′-OEt-^{5m}UTP (**6a**), 2′-OPr-^{5m}UTP (**6b**), 2′-OBu-^{5m}UTP (**6c**), and 2′-O^{i}Pr-^{5m}UTP (**6d**).

・Scheme S2. Synthesis of 2′-HE-^{5m}UTP (**11**).

・Scheme S3. Synthesis of 2′-MOE-^{5m}UTP (**13**).

・Figure S1. Polymerase incorporation of 2′-*O*-alkyl-^{5m}UTPs with a DNA primer.

・Figure S2. Polymerase incorporation of 2′-*O*-alkyl-^{5m}UTPs with DNA primer at different Mn^{2+} concentrations.

・Figure S3. Polymerase incorporation of 2′-*O*-alkyl-^{5m}UTPs with 2′-OMe modified primer at different Mn^{2+} concentrations.

・Figure S4. Enzymatic synthesis of ON_^{i}Pr, ON_HE and ON_MOE at different Mn^{2+} concentrations and incubation time.

・Figure S5. Ion pair RP-HPLC analysis of dTTP and 2′-O-alkyl ^{5m}UTPs.

・Figure S6–S30. NMR spectra of the prepared compounds.

Oligonucleotide sequences

Table S1. Sequences of primers and templates that were used in this study.

Name	Sequence	Notes
Primer**1**	5′-FAM-GGATTAGCGAACAGGCCATACCTTT-3'	
Primer**2**	5′-FAM-GGAUUAGCGAACAGGCCAUACCUUU-3'	1
Template**1**	5′-TCGACAAAAAAAAGGTATGGCCTGTTCGCTAATCC-3'	
Primer**3**	5′-FAM-UCGCCUUGCCGGAUCGCAGA-3'	1
Template**2**	5′-AAGGCAGCCACAGCGATTTC-$(N)_{30}$-TCTGCGATCCGGCAAGGCGA-3'	2

1: 2′-OMe is shown in red.

2: N means mix of A, G, C and T

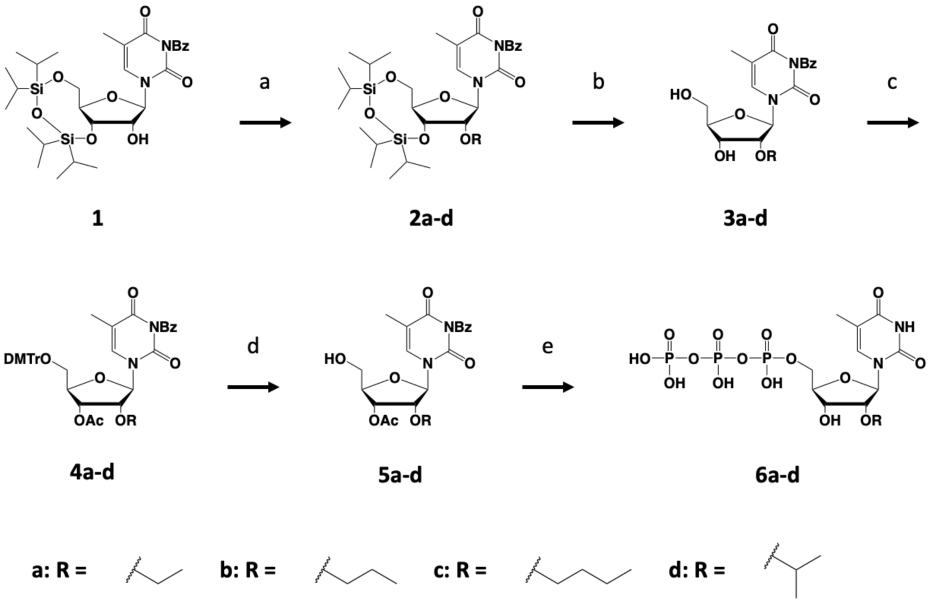

Scheme S1. Synthesis of 2′-OEt-^{5m}UTP (**6a**), 2′-OPr-^{5m}UTP (**6b**), 2′-OBu-^{5m}UTP (**6c**), and 2′-O^{i}Pr-^{5m}UTP (**6d**). Reagents and conditions: (a) alkyl iodite (iodoethane for 2a, 1-iodopropane for 2b, 1-iodobutane for 2c, 2-iodopropane for 2d), Ag_2O, toluene, 60–80 ℃, 13–15 h. Yields 29% for 2a; 23% for 2b; 18% for 2c; 23% for 2d. (b) TBAF, THF, rt, 30 min. Yields 91% for 3a; 77% for 3b; 78% for 3c; 86% for 3d. (c) DMTrCl, pyridine, rt, 4.5–6 h then DMAP, Ac_2O, rt, 13–18 h. Yields 71% for 4a; 69% for 4b; 75% for 4c; 74% for 4d. (d) TFA, CH_2Cl_2, rt, 30 min, Yields 76% for 5a; 72% for 5b; 75% for 5c; 68% for 5d. (e) (i) 2-chloro-4*H*-1,3,2-benzodioxaphosphorin-4-one, pyridine, dioxane, rt, 30 min. (ii) $(nBu_3NH)_2H_2P_2O_7$, DMF, Bu_3N, rt, 1 h. (iii) I_2, pyridine/H_2O, 0 ℃–rt, 30 min. (iv) aq. NH_3, rt, 2 h, Yields 22% for 6a; 46% for 6b; 10% for 6c; 16% for 6d. over four steps.

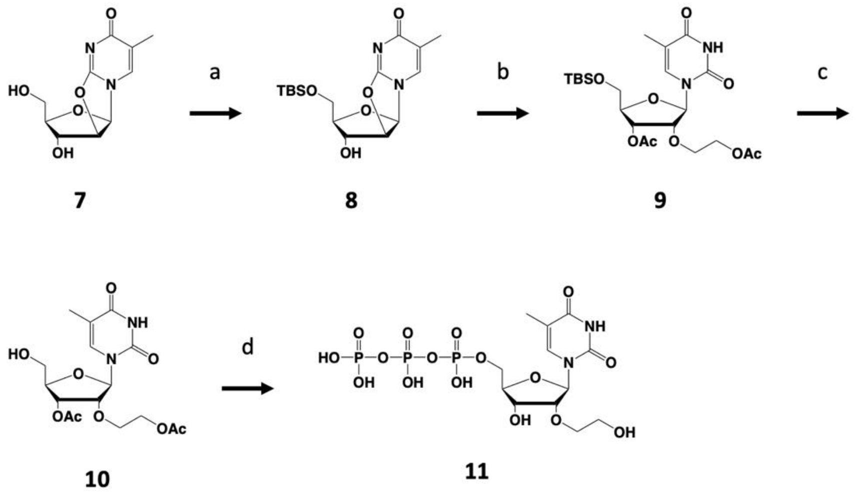

Scheme S2. Synthesis of 2′-HE-^{5m}UTP (**11**).

Reagents and conditions: (a) TBSCl, imidazole, DMF, rt, 5 h, 76%. (b) (i) BH_3·THF, ethylene glycol, $NaHCO_3$, 150 ℃, 24 h. (ii) DMAP, Ac_2O, pyridine, rt, 16 h, 50% over 2 steps. (c) TBAF, THF, rt, 30 min, 88%. (d) (i) 2-chloro-4*H*-1,3,2-benzodioxaphosphorin-4-one, pyridine, dioxane, rt, 30 min. (ii) $(nBu_3NH)_2H_2P_2O_7$, DMF, Bu_3N, rt, 1 h. (iii) I_2, pyridine/H_2O, 0 ℃, 30 min. (iv) aq. NH_3, rt, 2 h, 16%.

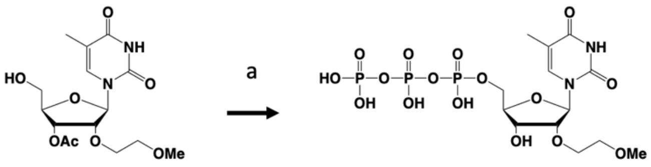

12

13

Scheme S3. Synthesis of 2′-MOE-^{5m}UTP (**13**).

Reagents and conditions: (a) (i) 2-chloro-4*H*-1,3,2-benzodioxaphosphorin-4-one, pyridine, dioxane, rt, 1 h. (ii) $(nBu_3NH)_2H_2P_2O_7$, DMF, Bu_3N, rt, 1.5 h. (iii) I_2, pyridine/H_2O, rt, 1 h. (iv) aq. NH_3, rt, 1 h, 16% over four steps.

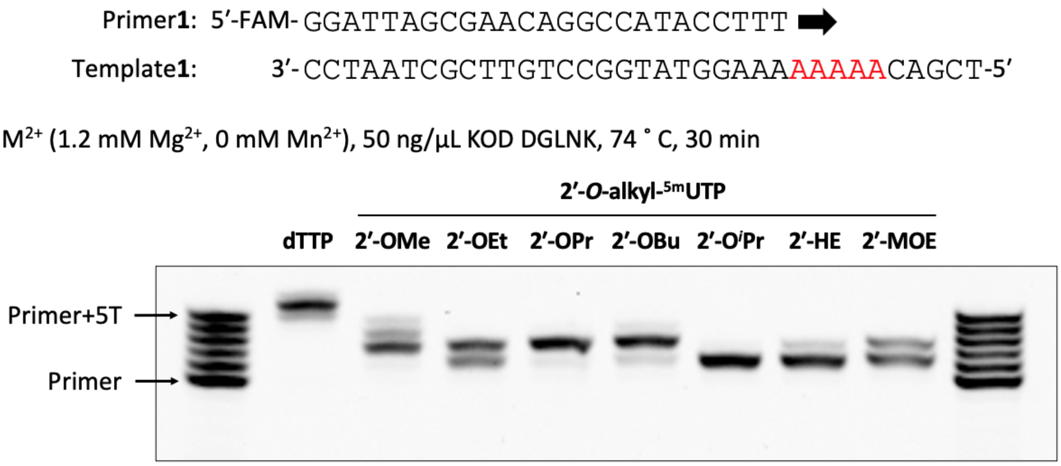

	Primer	Primer +1	Primer +2	Primer +3	Primer +4	Primer +5	Other
2′-OMe	0	3.1	50.2	20.9	9.1	0	16.7
2′-OEt	0	37.6	52.8	0	0	0	9.6
2′-OPr	0	4.8	85.0	0	0	0	10.2
2′-OBu	0	5.5	72.9	11.2	0	0	10.4
2′-O^{i}Pr	0	87.7	2.8	0	0	0	9.5
2′-HE	0	75.5	14.4	0	0	0	10.1
2′-MOE	0	53.3	36.6	0	0	0	10.1

Figure S1. Polymerase incorporation of 2′-*O*-alkyl-^{5m}UTPs with a DNA primer. Primer extension was performed with a 0.4 µM 5′-FAM-labeled DNA primer, 0.5 µM DNA template, 1 × KOD Dash® buffer, and 0.2 mM 2′-*O*-alkyl-^{5m}UTPs by incubation with 50 ng/µL KOD DGLNK at 74 ℃ for 30 min. The table shows the ratio of each band to the total band in each lane. These were calculated by using image lab.

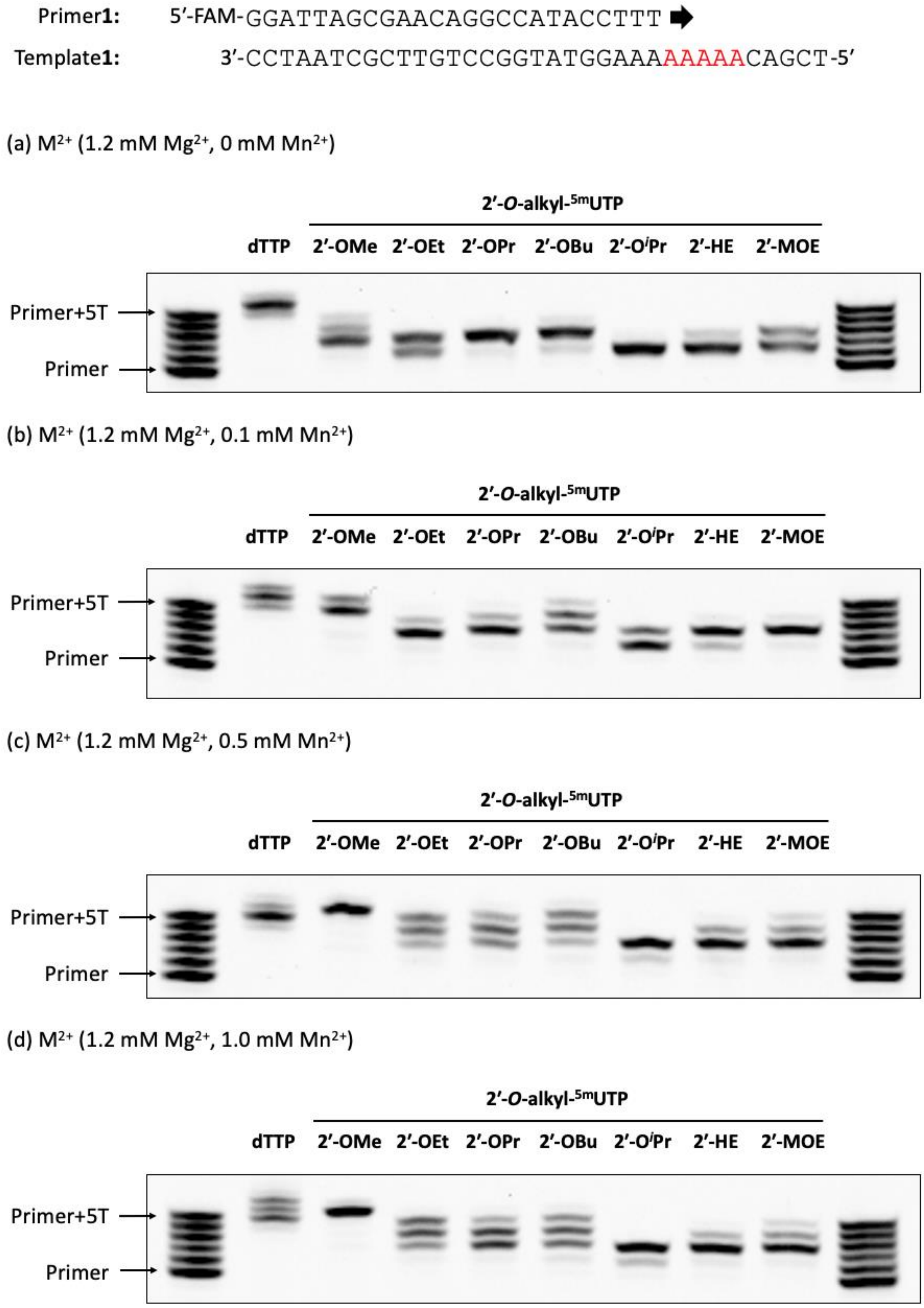

Figure S2. Polymerase incorporation of 2′-*O*-alkyl-^{5m}UTPs with DNA primer at different Mn^{2+} concentrations. Primer extension was performed with 0.4 μM 5′-FAM-labeled DNA primer, 0.5 μM DNA template, 1×KOD Dash® buffer, 0.2 mM 2′-*O*-alkyl-^{5m}UTPs and 0 mM (a), 0.1 mM (b), 0.5 mM (c), 1.0 mM (d) $MnSO_4$ by incubation with 50 ng/μL KOD DGLNK at 74 ℃ for 30 min.

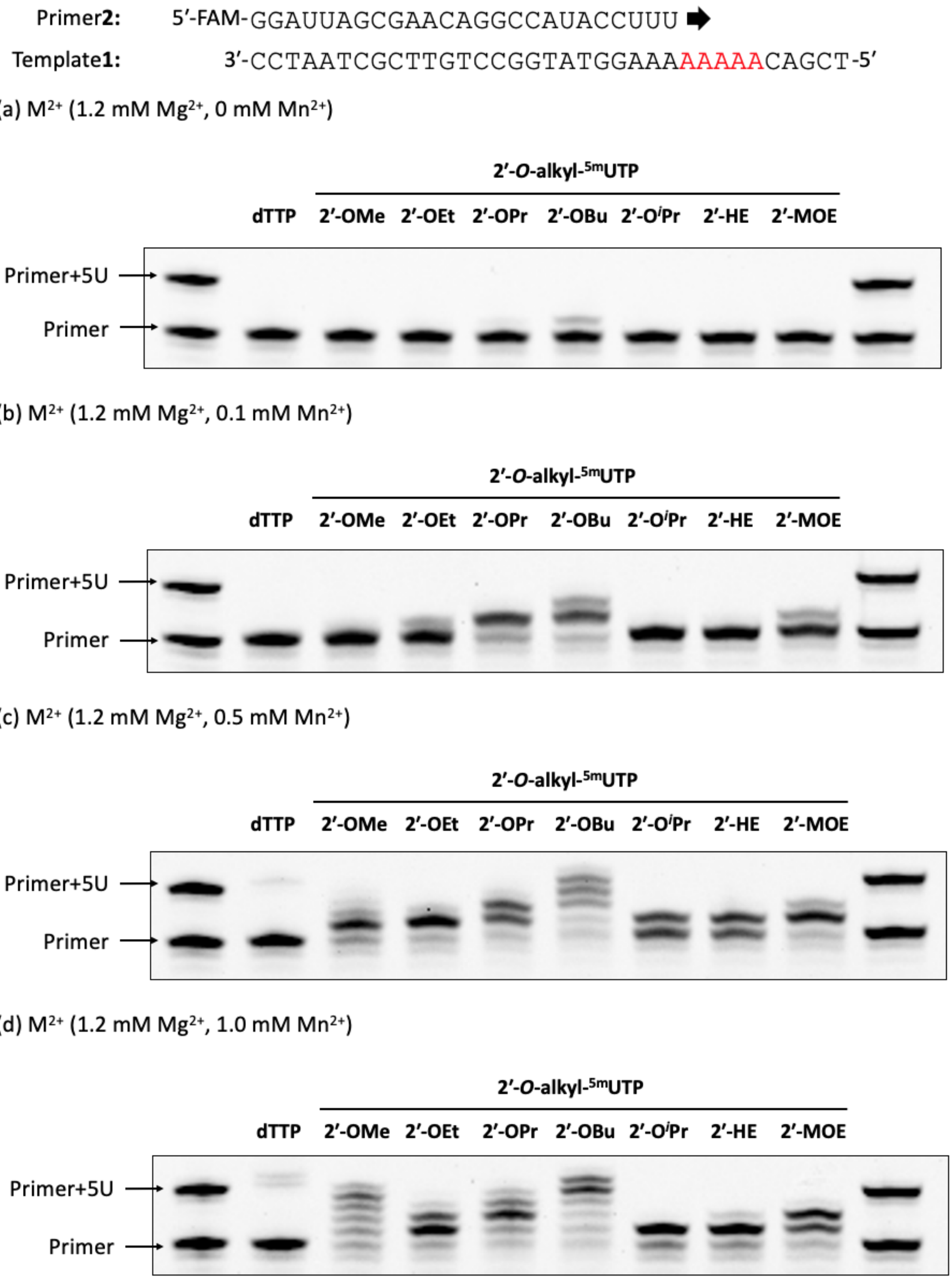

Figure S3. Polymerase incorporation of 2′-*O*-alkyl-^{5m}UTPs with 2′-OMe modified primer at different Mn^{2+} concentrations.

Primer extension was performed with 0.4 μM 5′-FAM-labeled 2′-OMe modified primer, 0.5 μM DNA template, 1× KOD Dash® buffer, 0.2 mM 2′-*O*-alkyl-^{5m}UTPs and 0 mM (a), 0.1 mM (b), 0.5 mM (c), 1.0 mM (d) MnSO$_4$ by incubation with 50 ng/μL KOD DGLNK at 74 ℃ for 30 min.

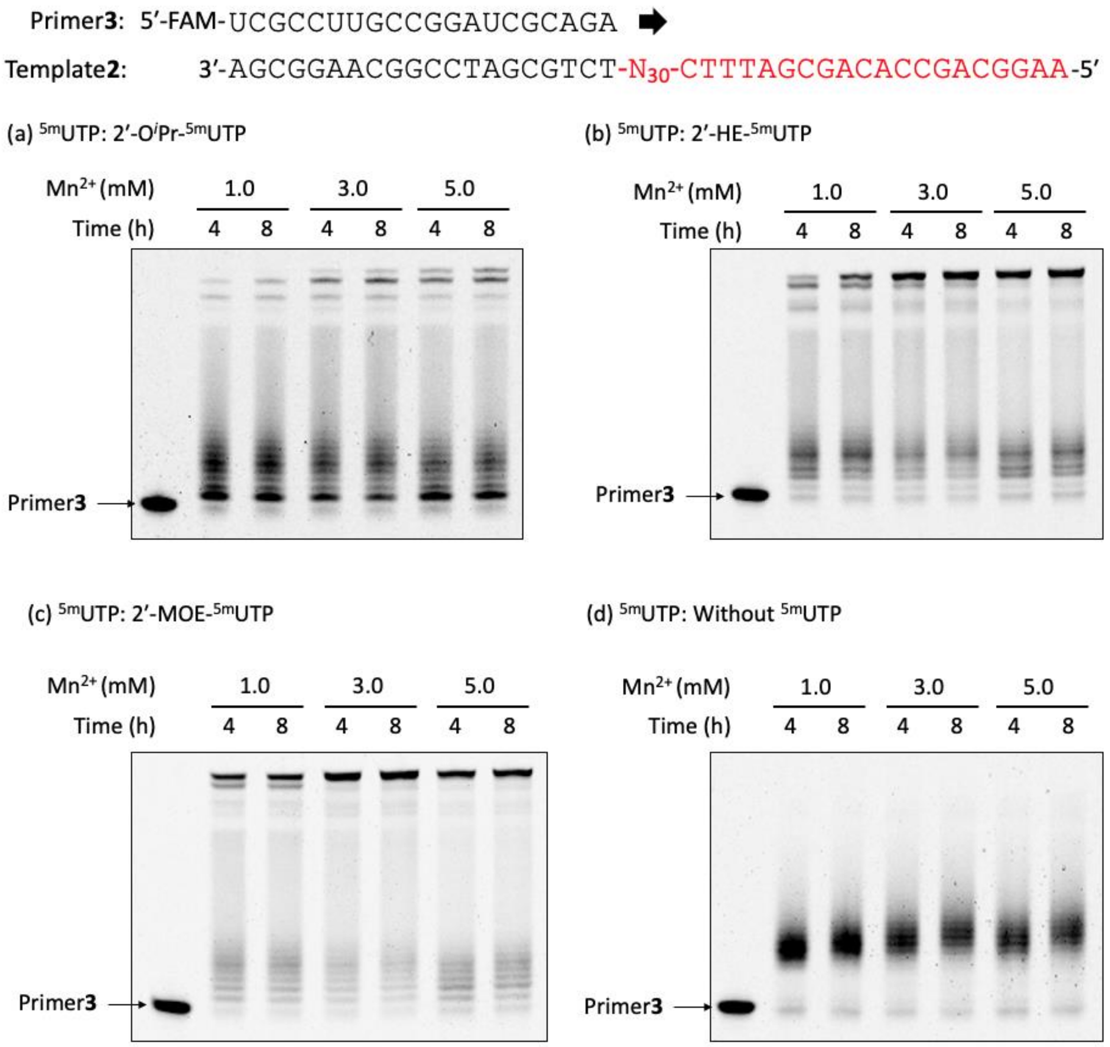

Figure S4. Enzymatic synthesis of ON_^{i}Pr, ON_HE and ON_MOE at different Mn^{2+} concentrations and incubation time.

Primer extension was performed with 0.4 µM 5′-FAM-labeled 2′-OMe modified primer, 0.5 µM DNA template, 1× KOD Dash® buffer, 1.0, 3.0, or 5.0 mM $MnSO_4$, 0.2 mM 2′-OMe-ATP, 0.2 mM 2′-OMe-GTP, 0.2 mM 2′-OMe-CTP and 2′-O^{i}Pr-^{5m}UTP (a), 2′-HE-^{5m}UTP (b) 2′-MOE-^{5m}UTP (c), without ^{5m}UTP (d) by incubation with 300 ng/µL KOD DGLNK at 60 ℃ for 1 h, 60 ℃ to 74 ℃ for 4 or 8 h, 74 ℃ for 1 h. After extension, reaction mixture was treated with 95 mU/µL DNase1 at 37 ℃ for 30 min.

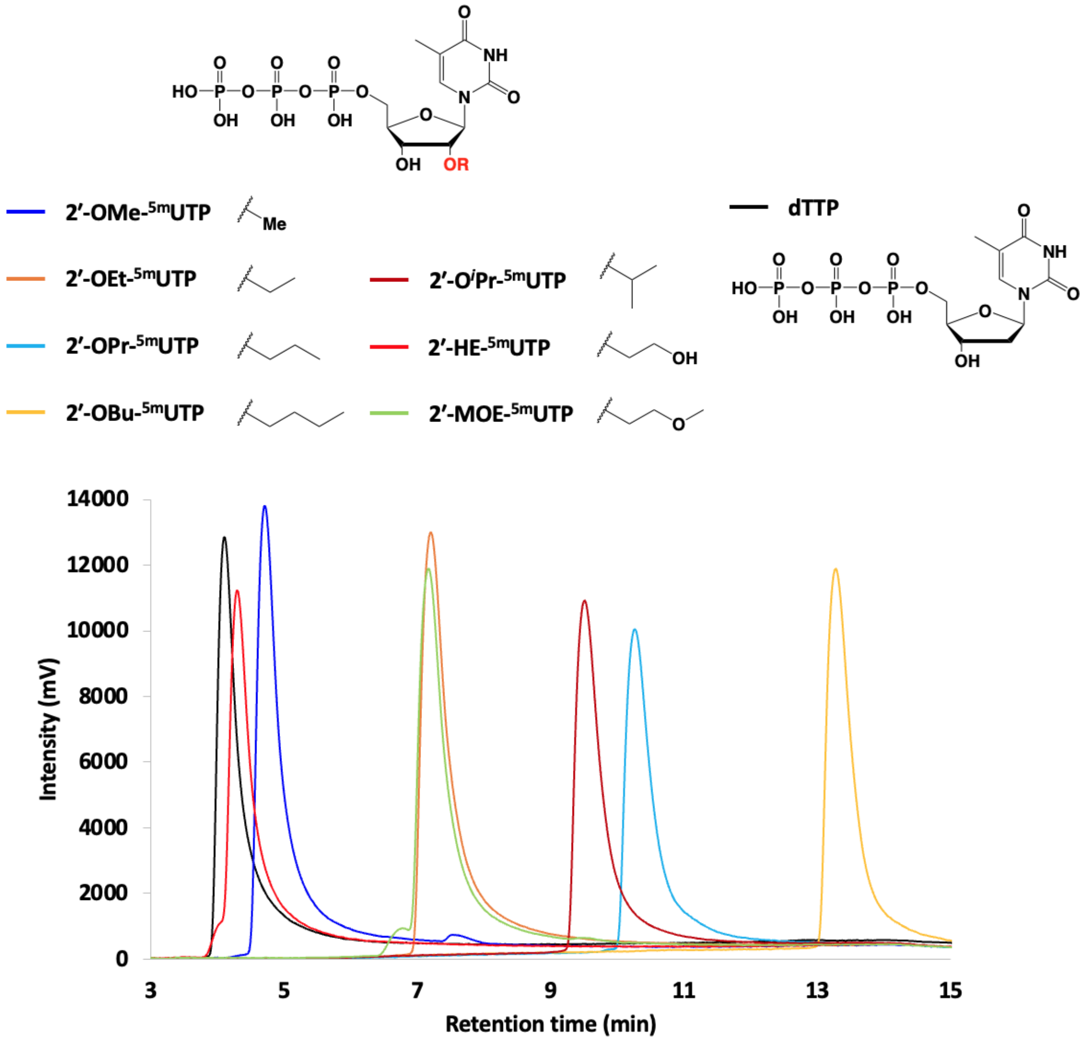

Figure S5. Ion pair RP-HPLC analysis of dTTP and 2′-O-alkyl-^{5m}UTPs.

Ion pair RP-HPLC was performed by the injection 700 pmol nucleotide triphosphates onto a XBridge Oligonucleotide BEH C18 column. The column temperature was 50 ℃, with a flow rate of 1.0 mL/min, and detection at 260 nm. Mobile phase A consisted of 100 mM triethylamine acetic acid solution (pH 6.9) and mobile phase B was acetonitrile. The column was initially maintained at 5% mobile phase B and then at a gradient of 5% to 20% over 15 min.

^{1}H NMR, $CDCl_3$, 400 MHz

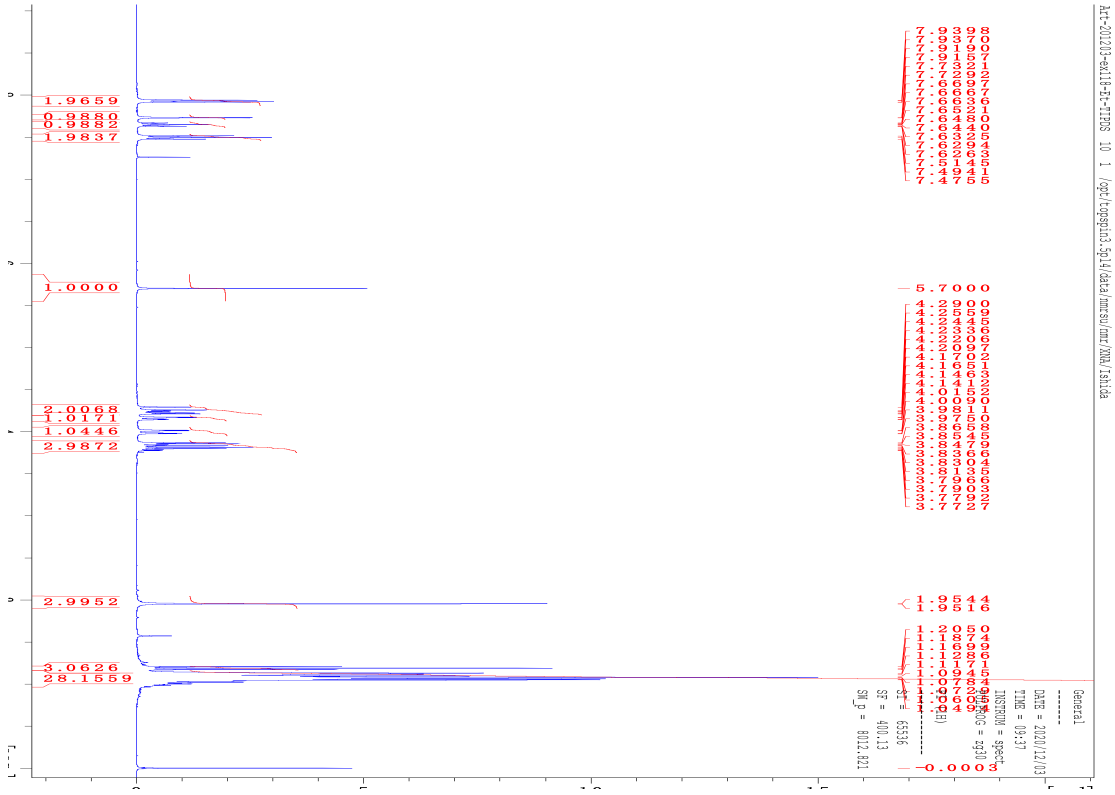

^{13}C NMR, $CDCl_3$, 100 MHz

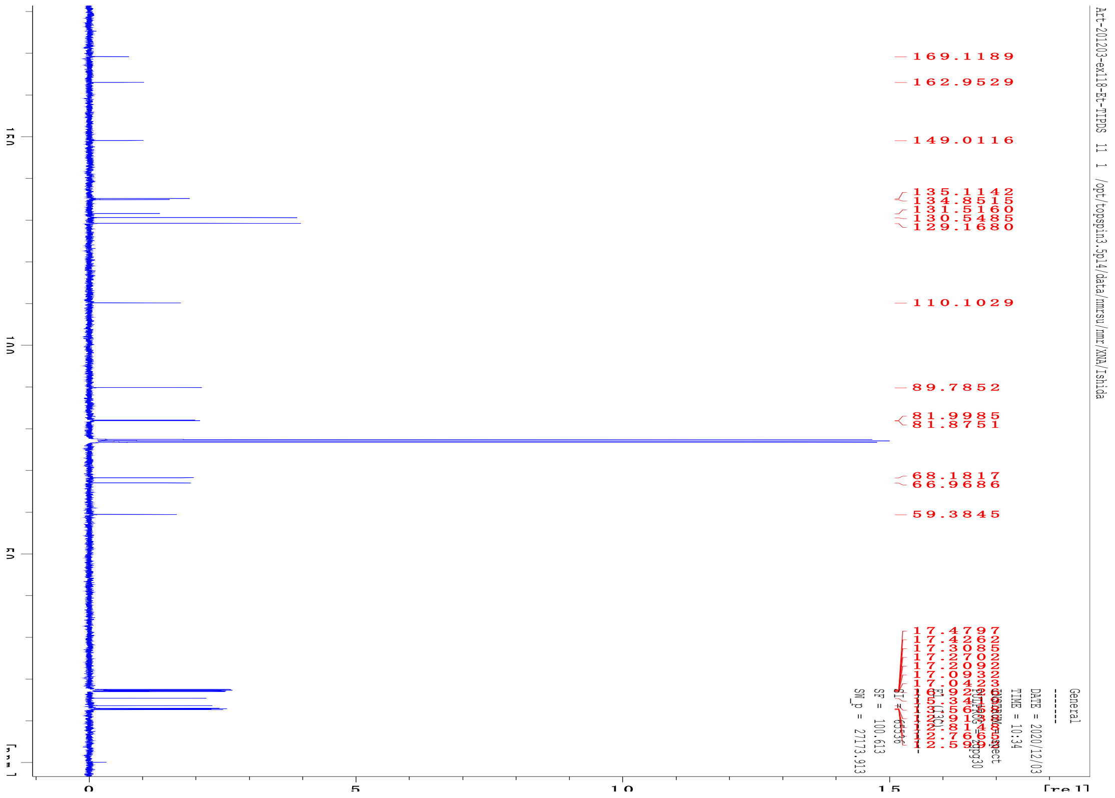

Figure S6. ^{1}H and ^{13}C NMR spectra of compound **2a**.

^1H NMR, $CDCl_3$, 400 MHz

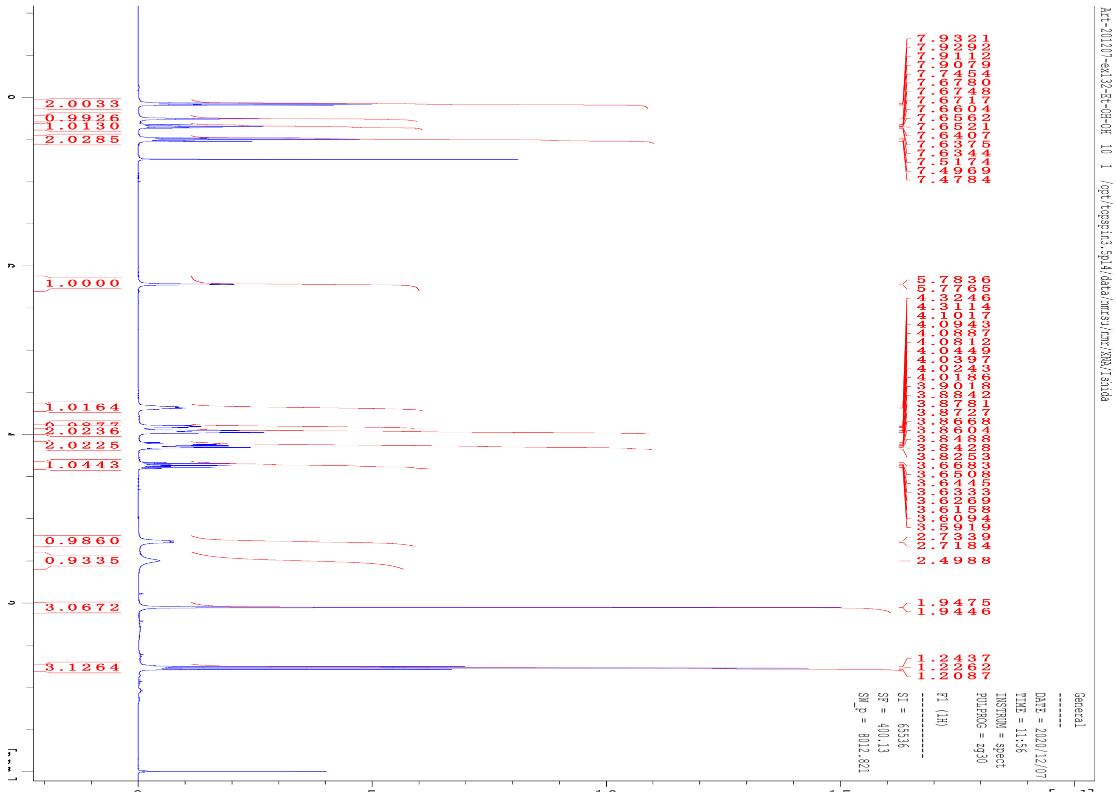

^{13}C NMR, $CDCl_3$, 100 MHz

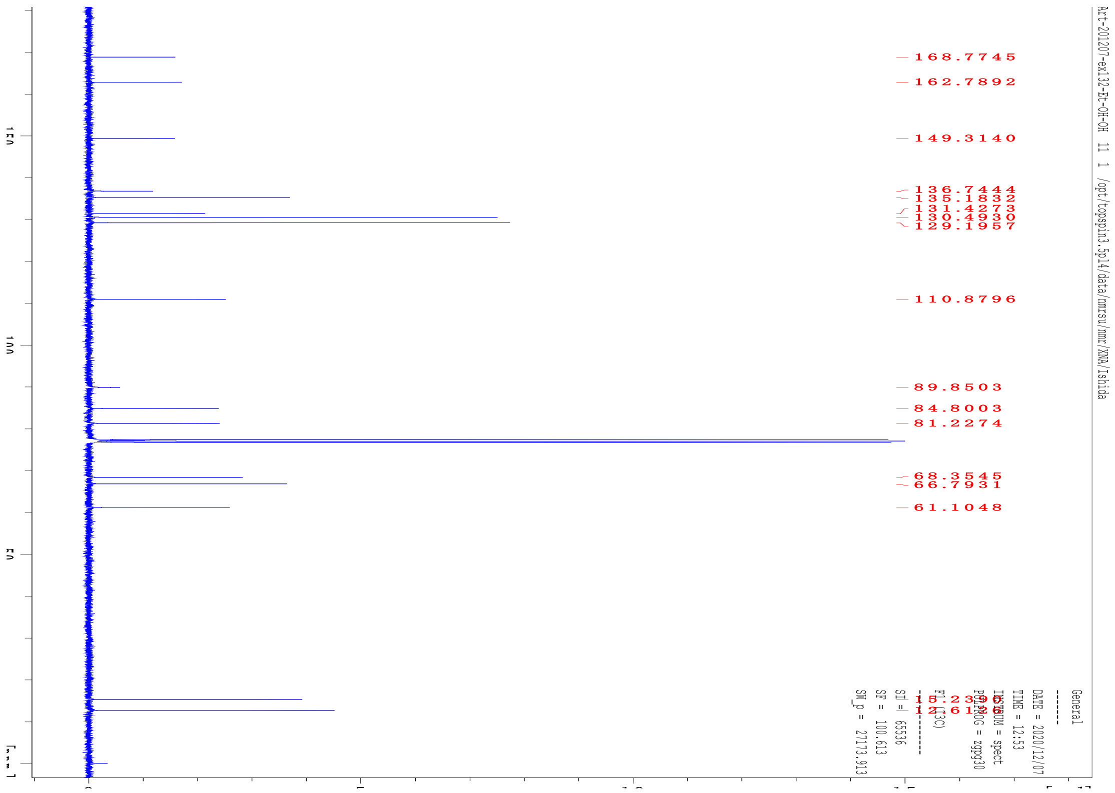

Figure S7. ^1H and ^{13}C NMR spectra of compound **3a**.

^{1}H NMR, $CDCl_3$, 400 MHz

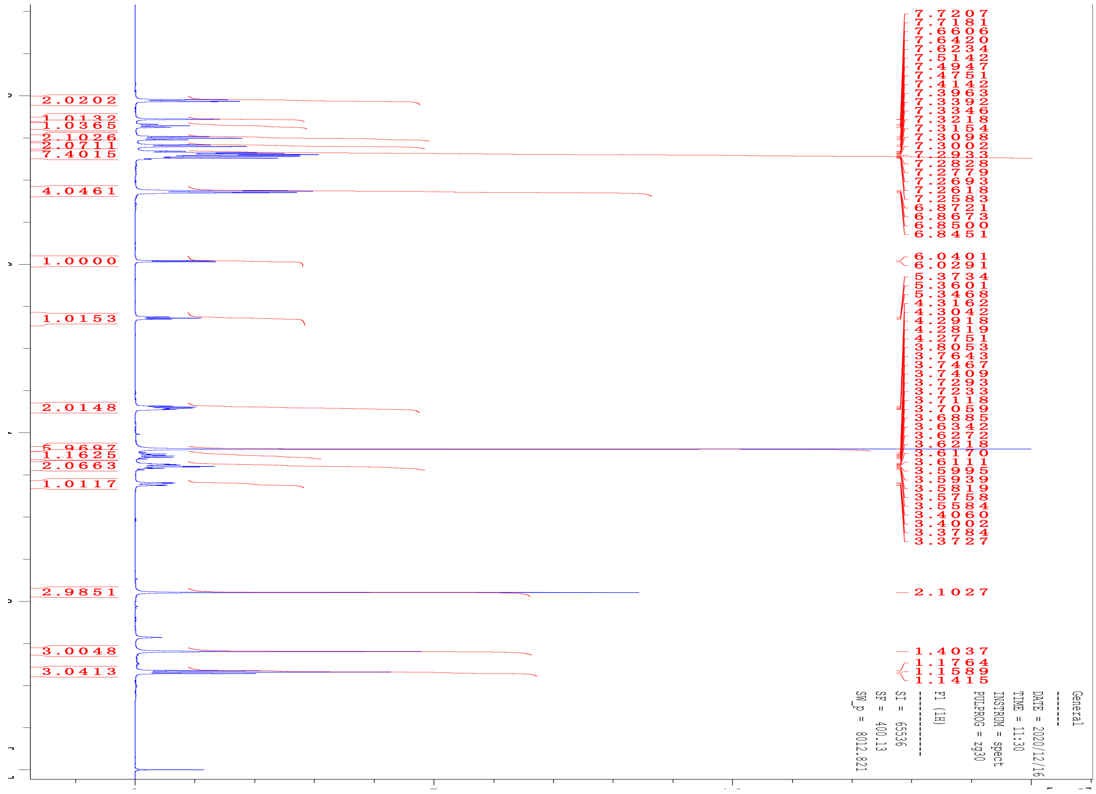

^{13}C NMR, $CDCl_3$, 100 MHz

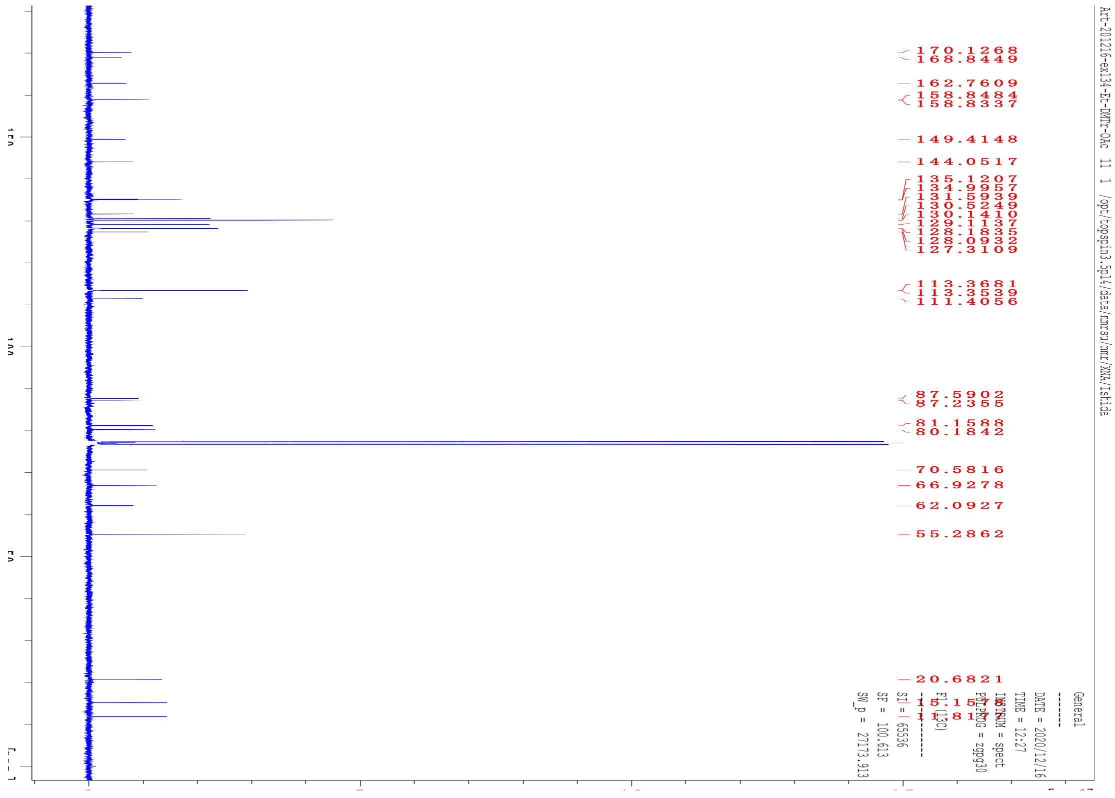

Figure S8. ^{1}H and ^{13}C NMR spectra of compound **4a**.

^{1}H NMR, $CDCl_3$, 400 MHz

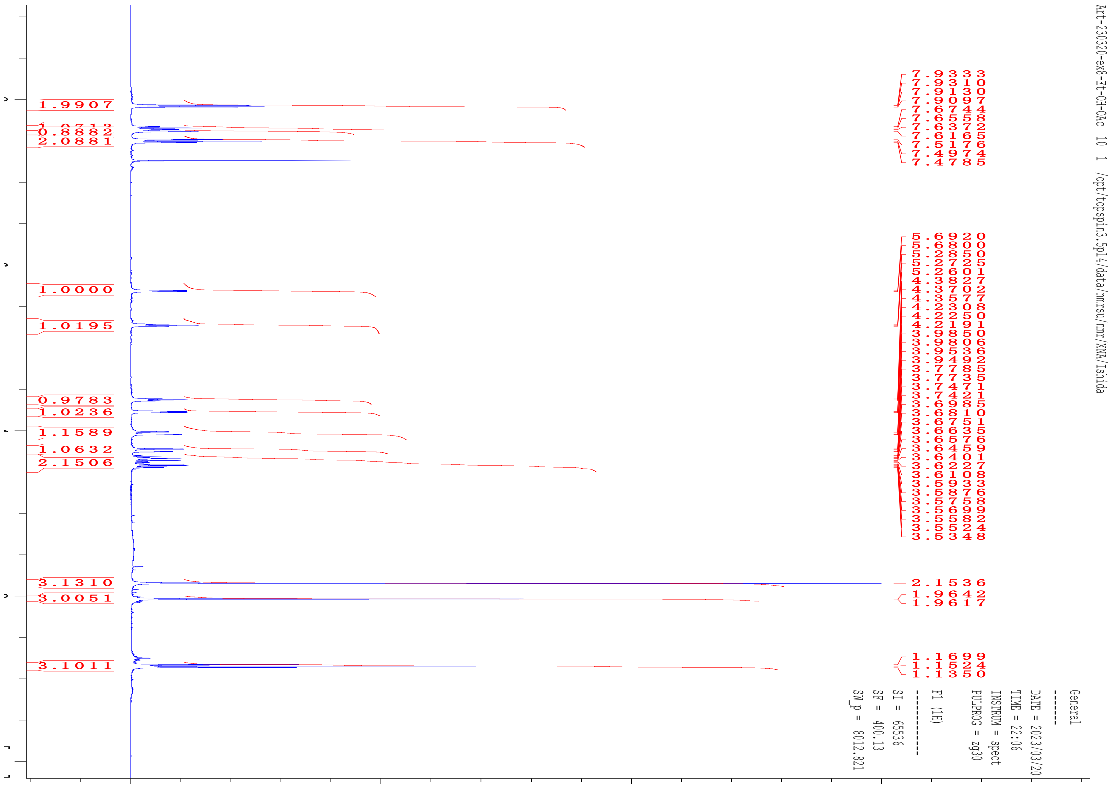

^{13}C NMR, $CDCl_3$, 100 MHz

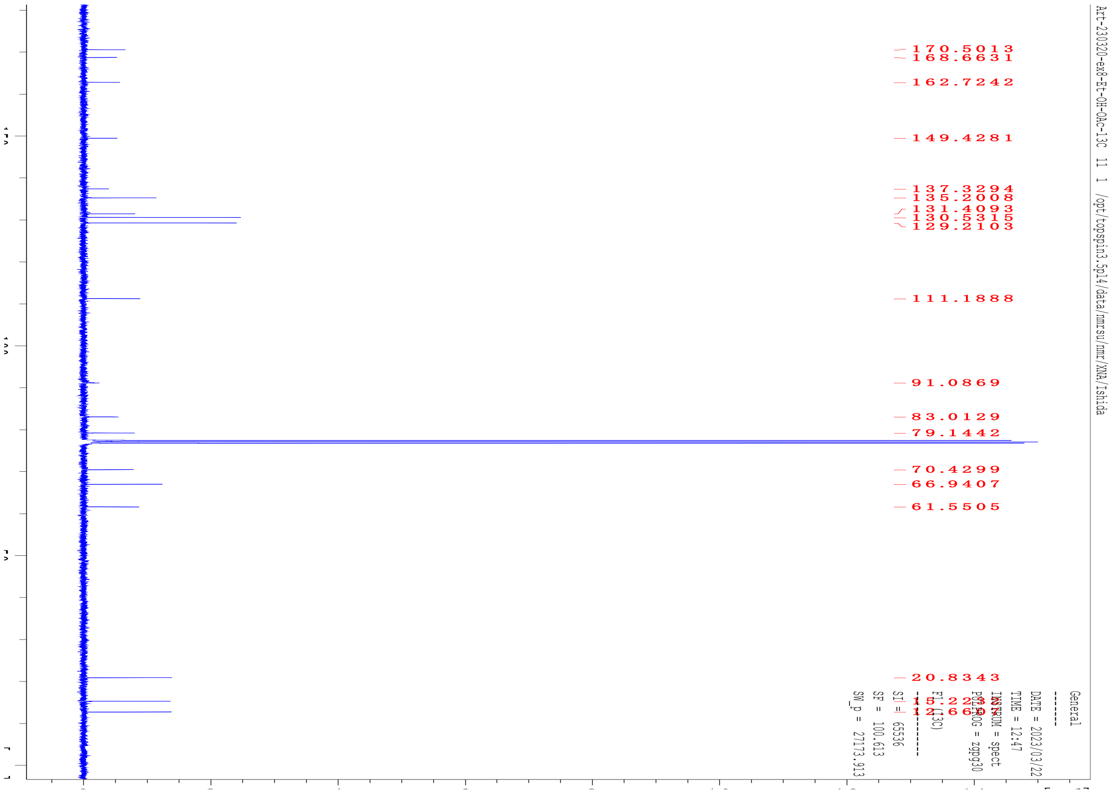

Figure S9. ^{1}H and ^{13}C NMR spectra of compound **5a**.

^{1}H NMR, D_2O, 400 MHz

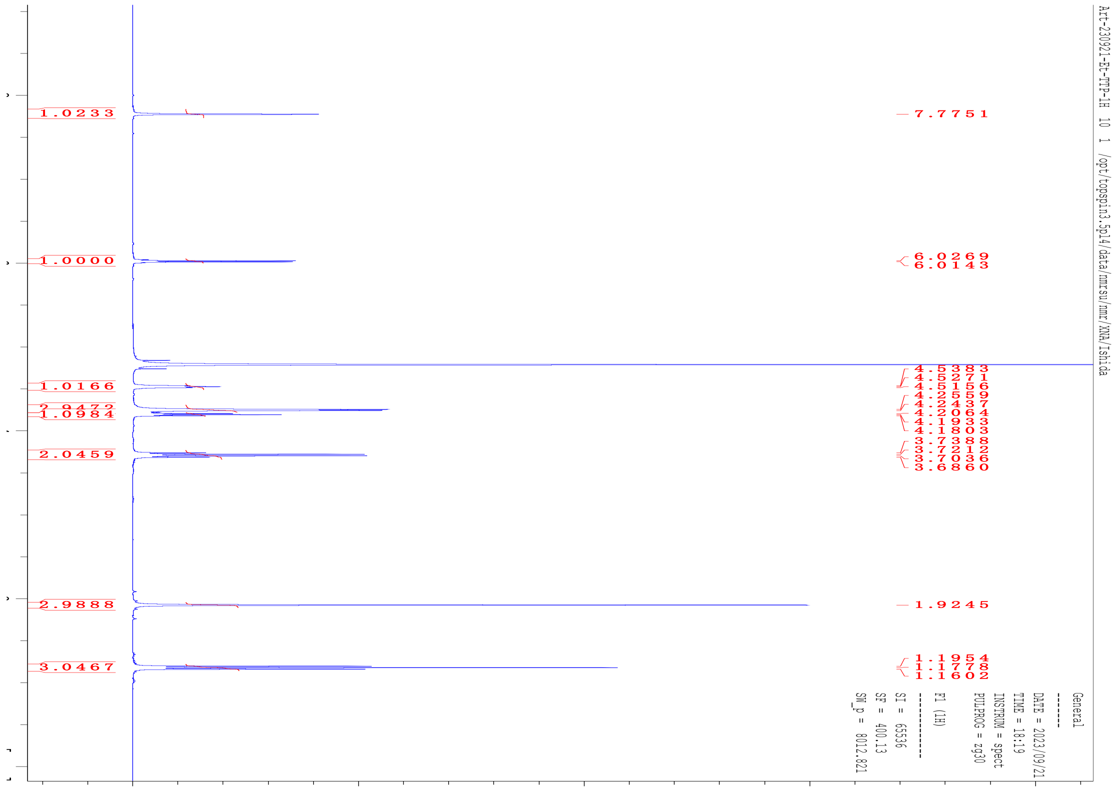

^{31}P NMR, D_2O, 120 MHz

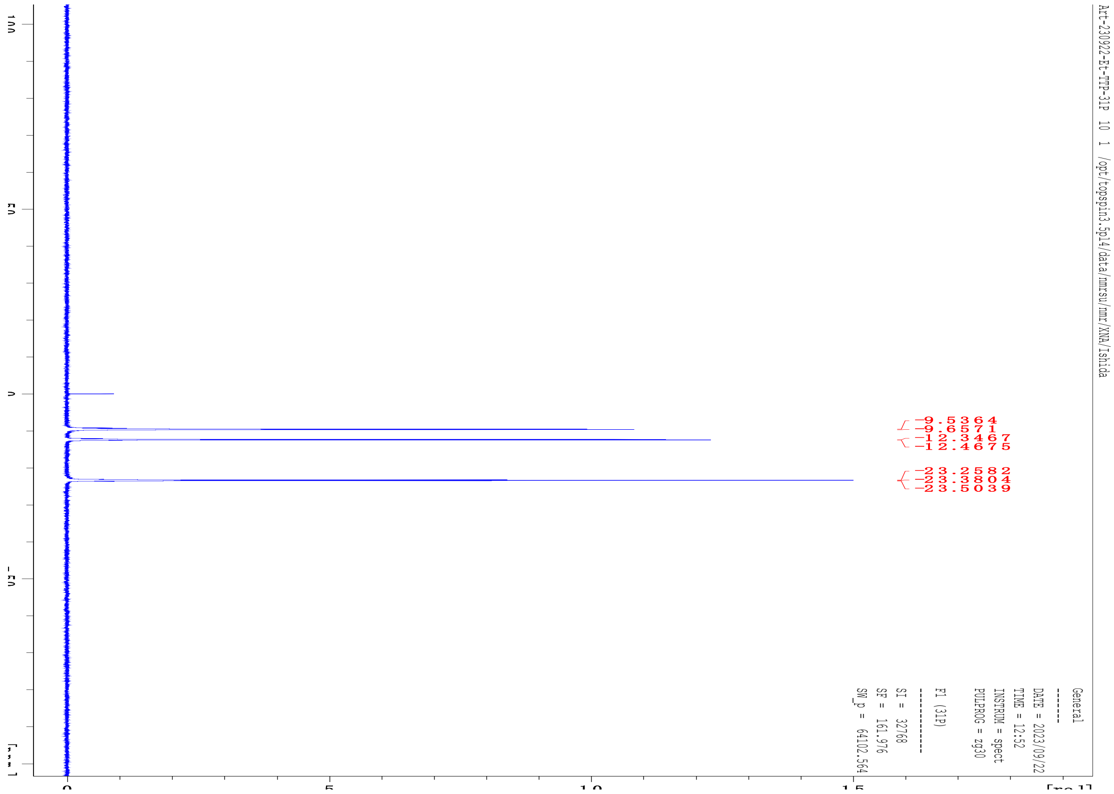

Figure S10. ^{1}H and ^{31}P NMR spectra of compound **6a**.

^{1}H NMR, $CDCl_3$, 400 MHz

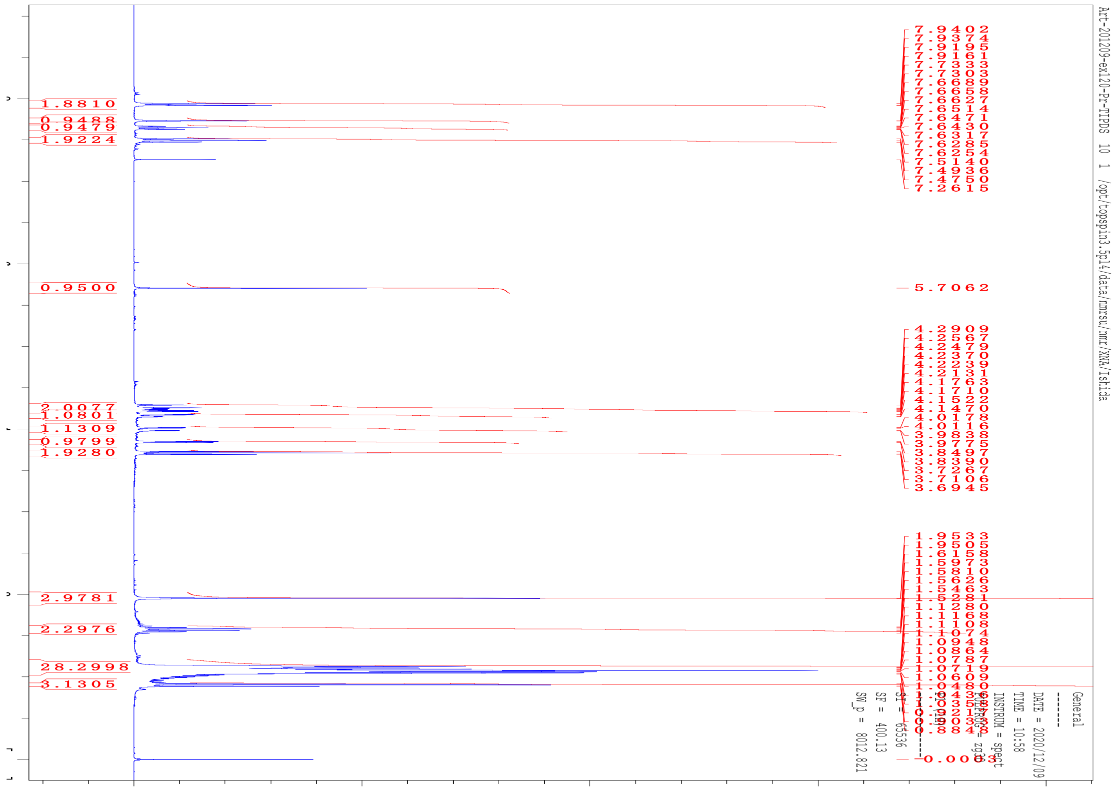

^{13}C NMR, $CDCl_3$, 100 MHz

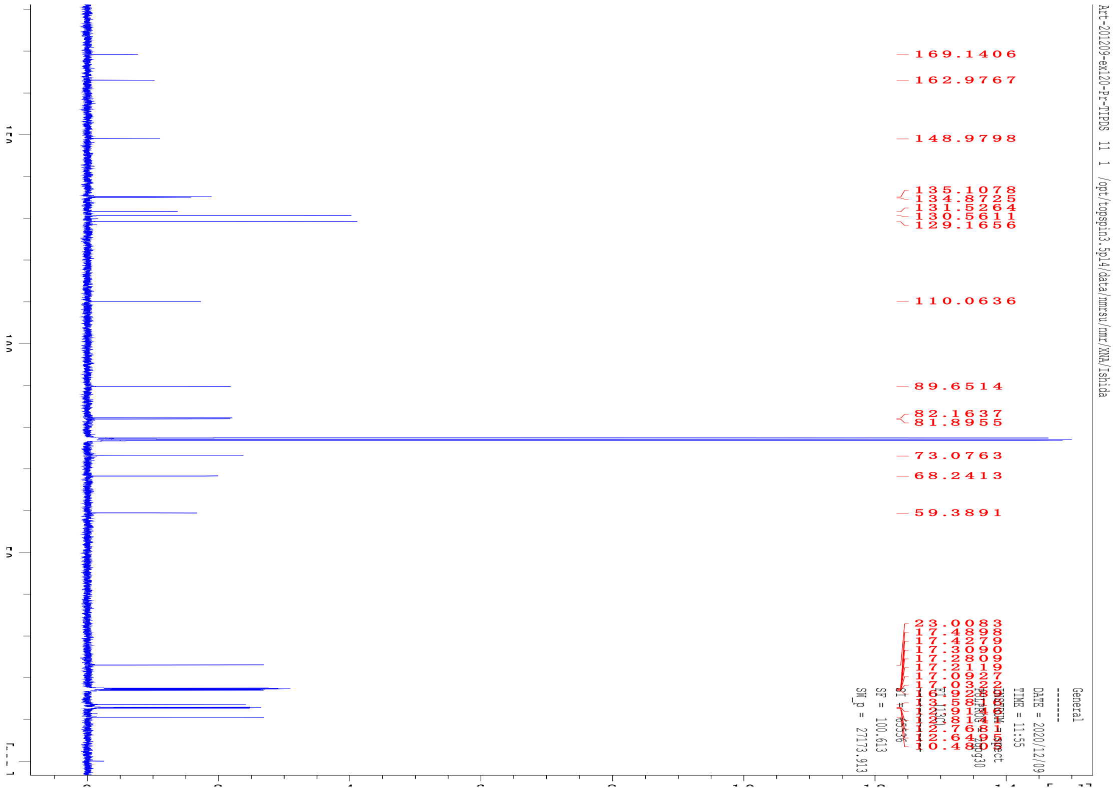

Figure S11. ^{1}H and ^{13}C NMR spectra of compound **2b**.

^{1}H NMR, $CDCl_3$, 400 MHz

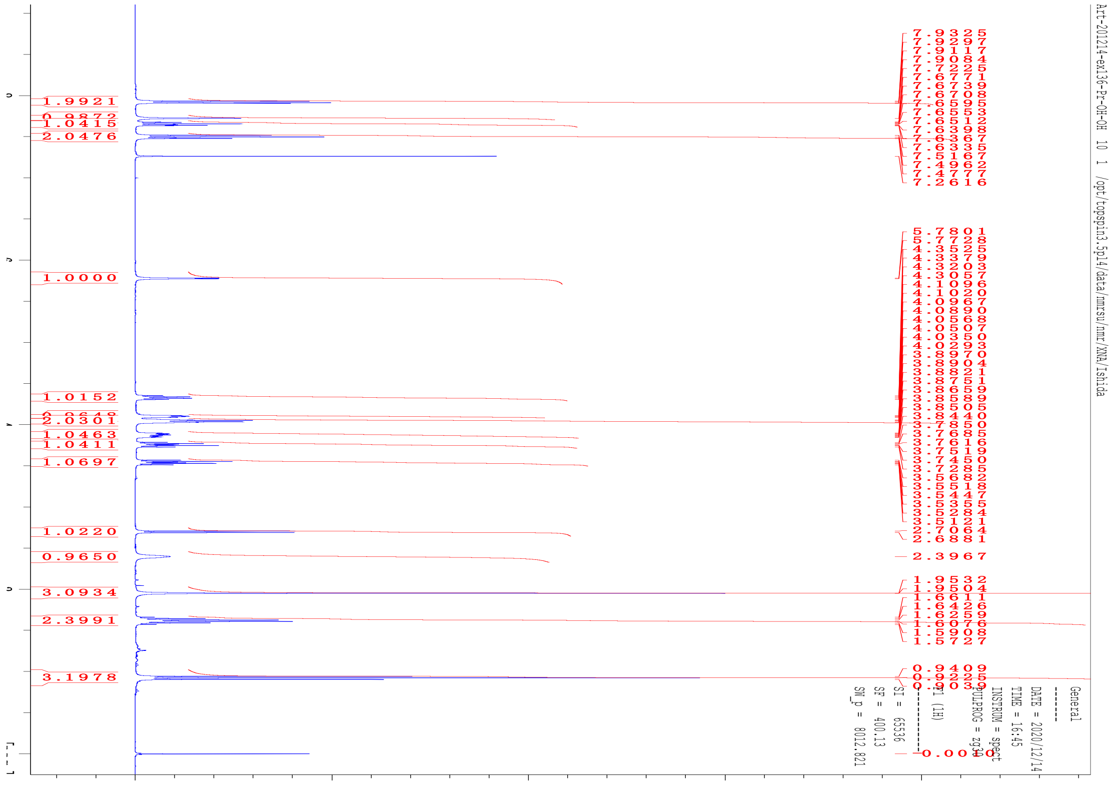

^{13}C NMR, $CDCl_3$, 100 MHz

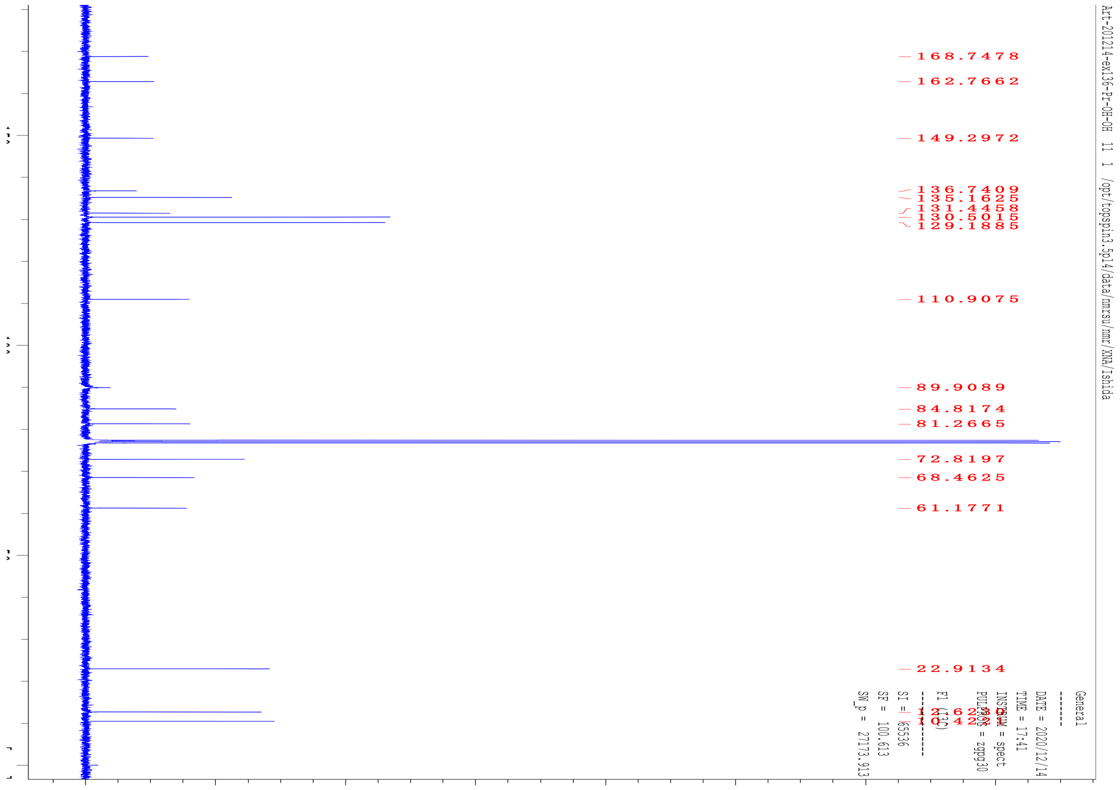

Figure S12. ^{1}H and ^{13}C NMR spectra of compound **3b**.

^1H NMR, $CDCl_3$, 400 MHz

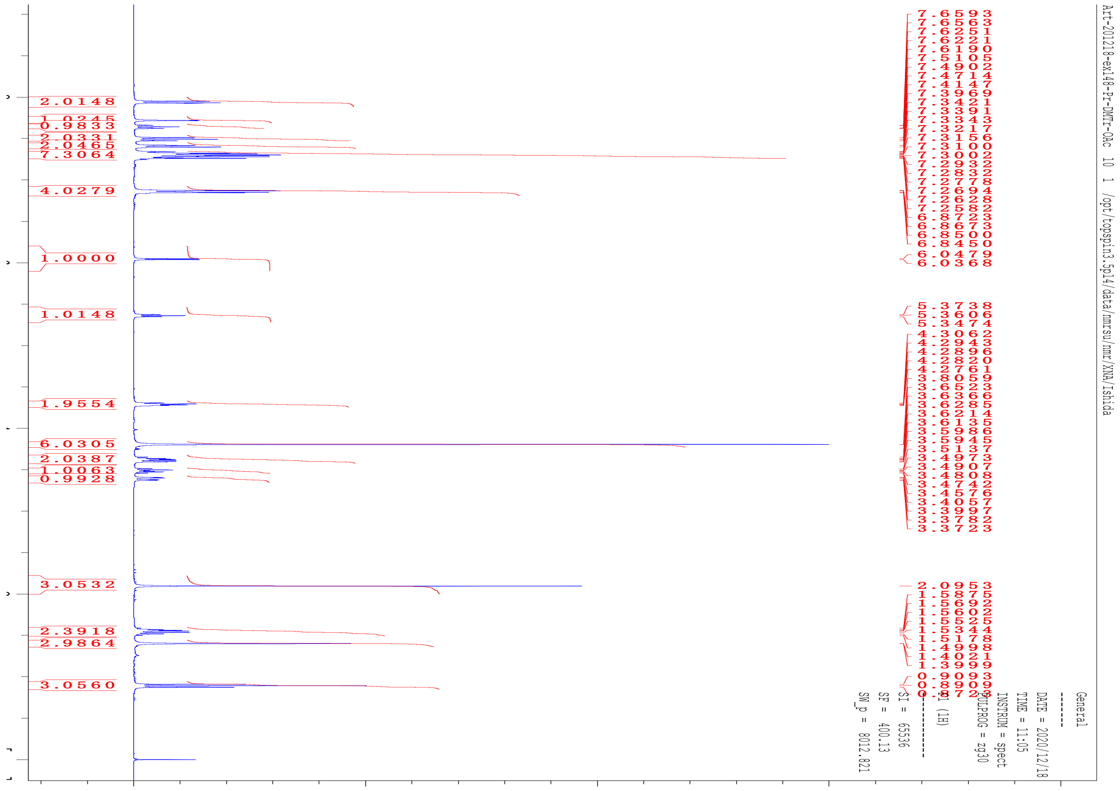

^{13}C NMR, $CDCl_3$, 100 MHz

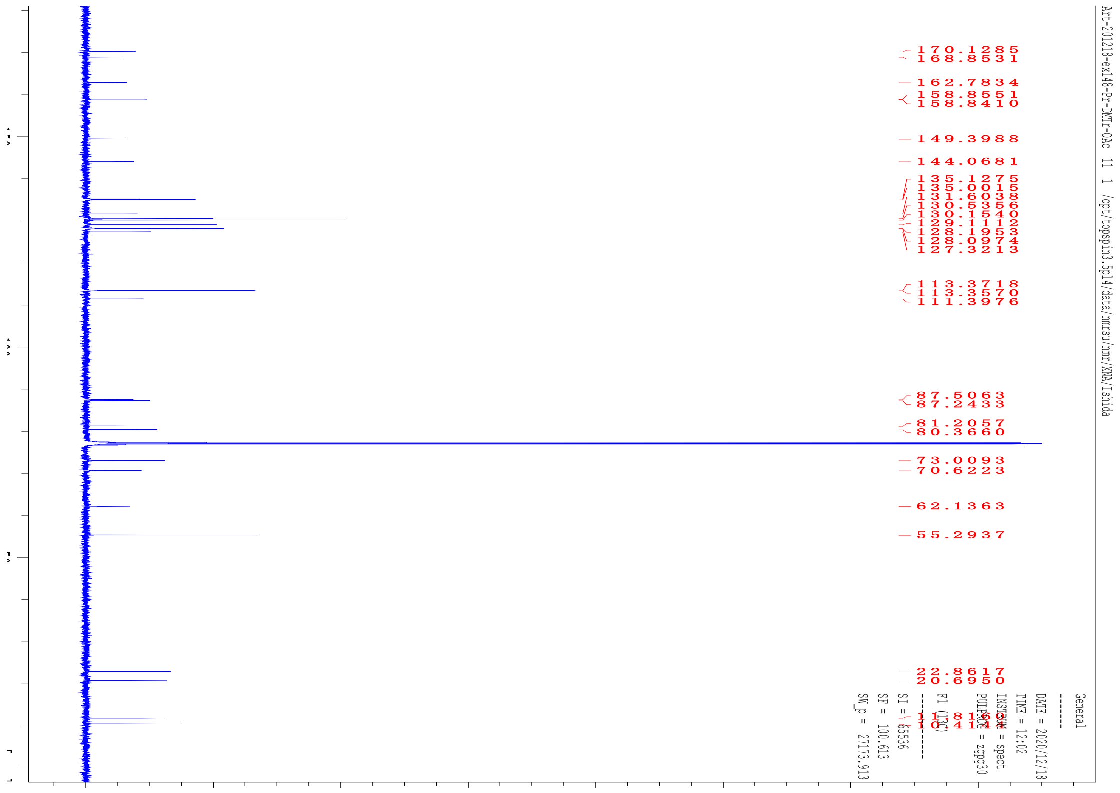

Figure S13. ^1H and ^{13}C NMR spectra of compound **4b**.

^{1}H NMR, $CDCl_3$, 400 MHz

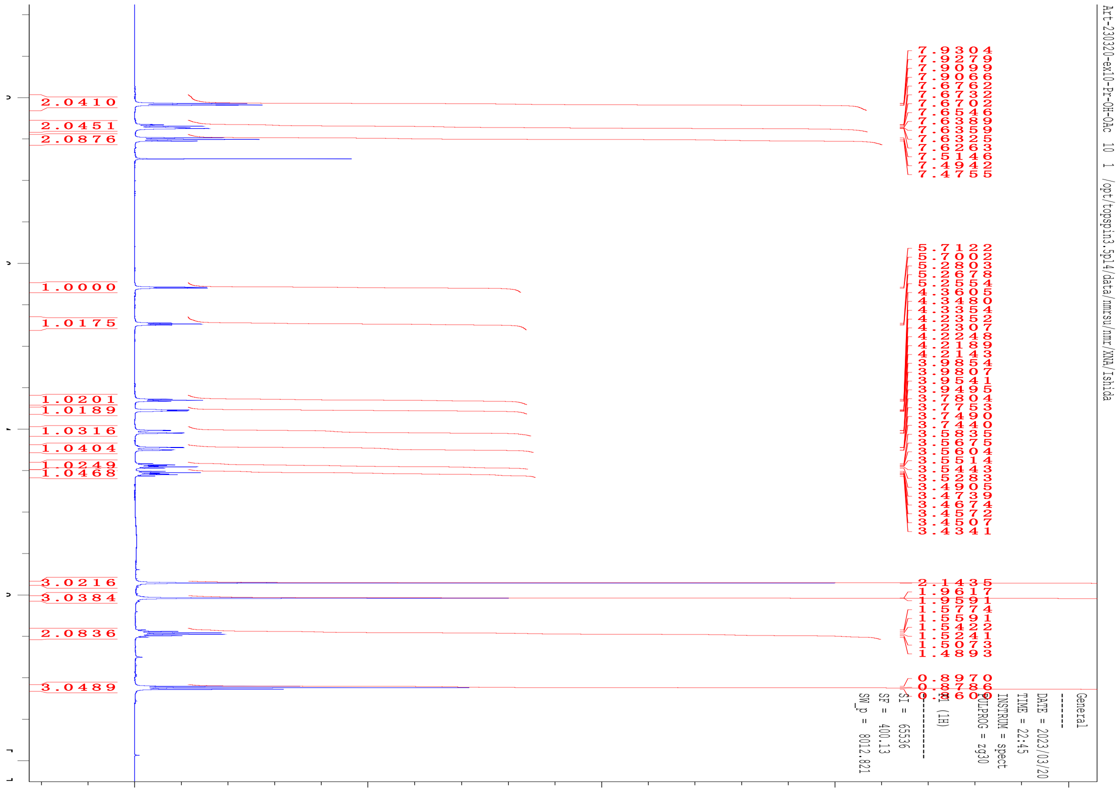

^{13}C NMR, $CDCl_3$, 100 MHz

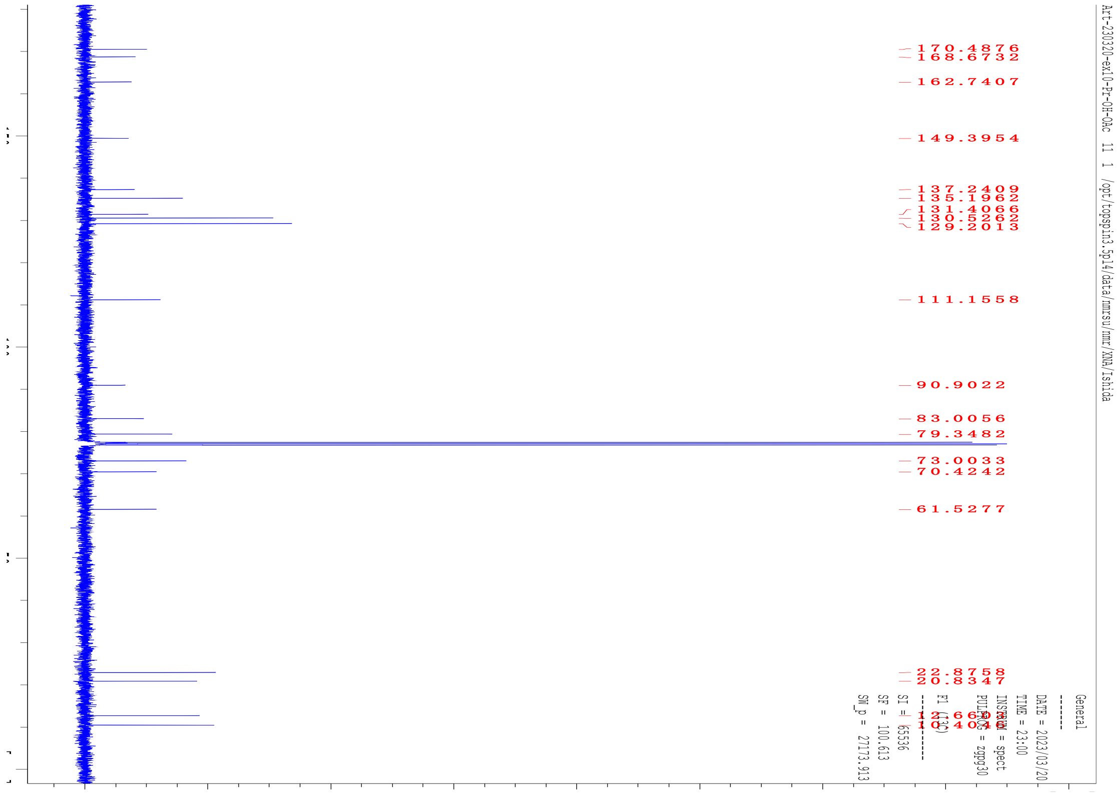

Figure S14. ^{1}H and ^{13}C NMR spectra of compound **5b**.

^{1}H NMR, D_2O, 400 MHz

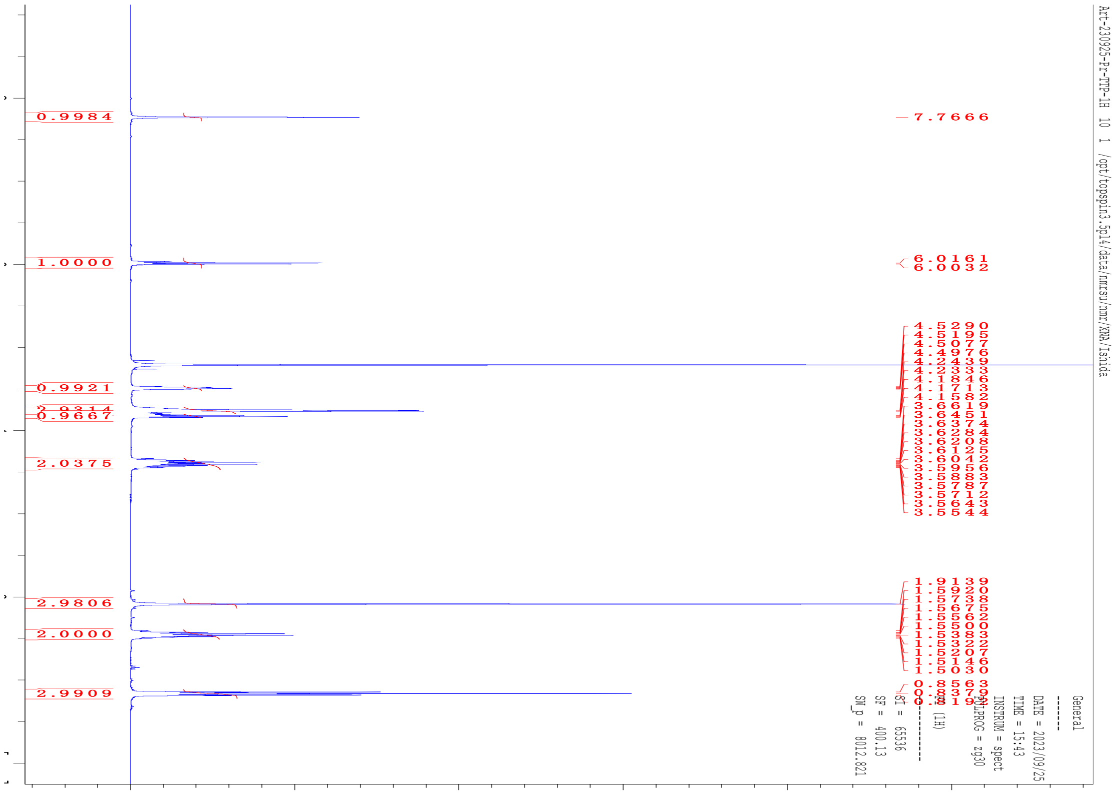

^{31}P NMR, D_2O, 120 MHz

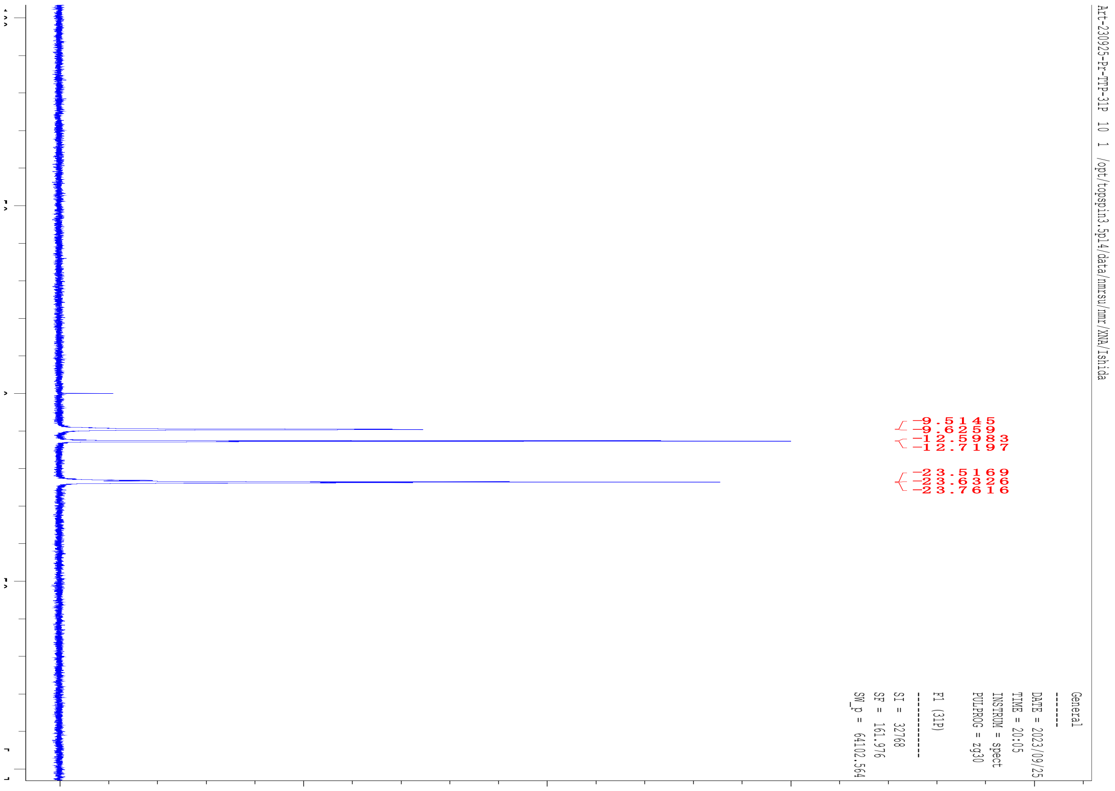

Figure S15. ^{1}H and ^{31}P NMR spectra of compound **6b**.

^{1}H NMR, $CDCl_3$, 400 MHz

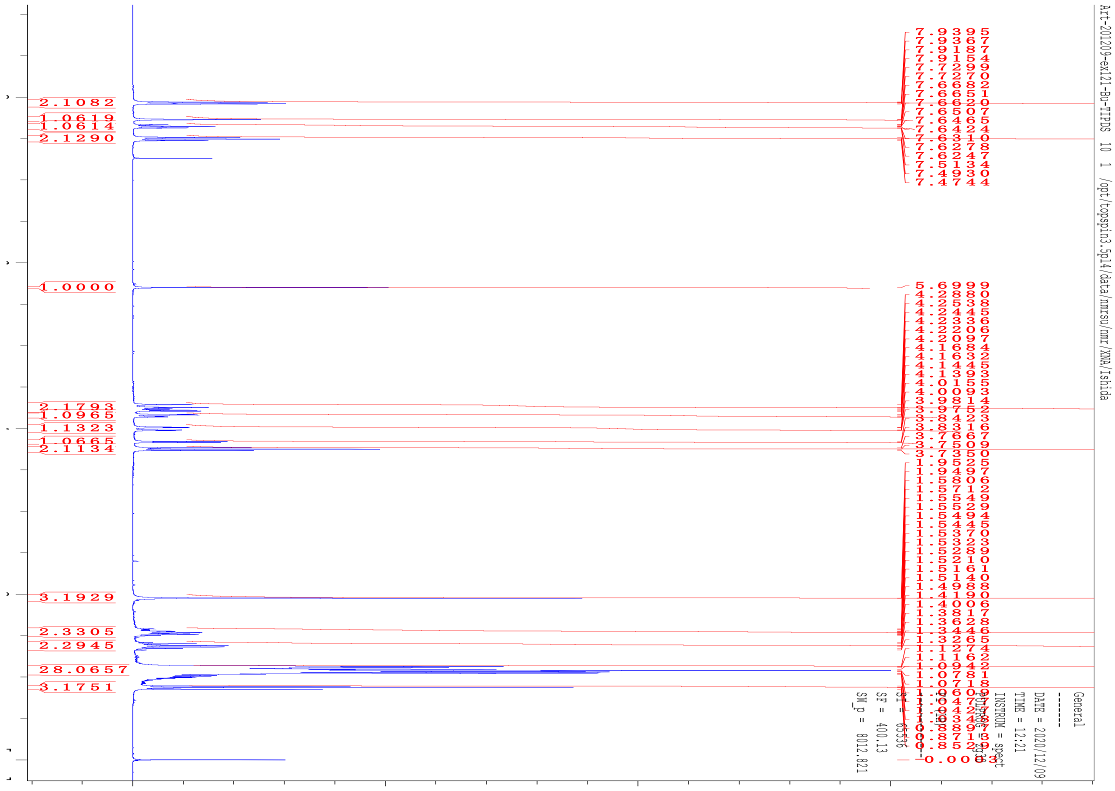

^{13}C NMR, $CDCl_3$, 100 MHz

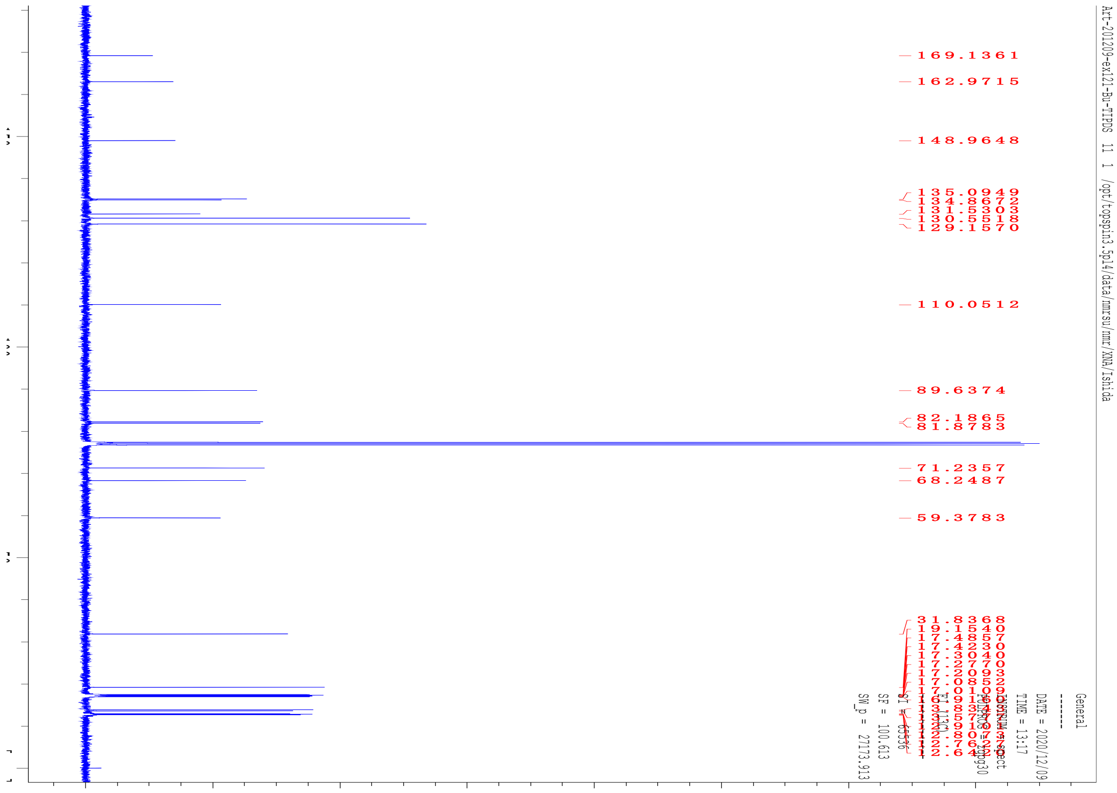

Figure S16. ^{1}H and ^{13}C NMR spectra of compound **2c**.

^{1}H NMR, $CDCl_3$, 400 MHz

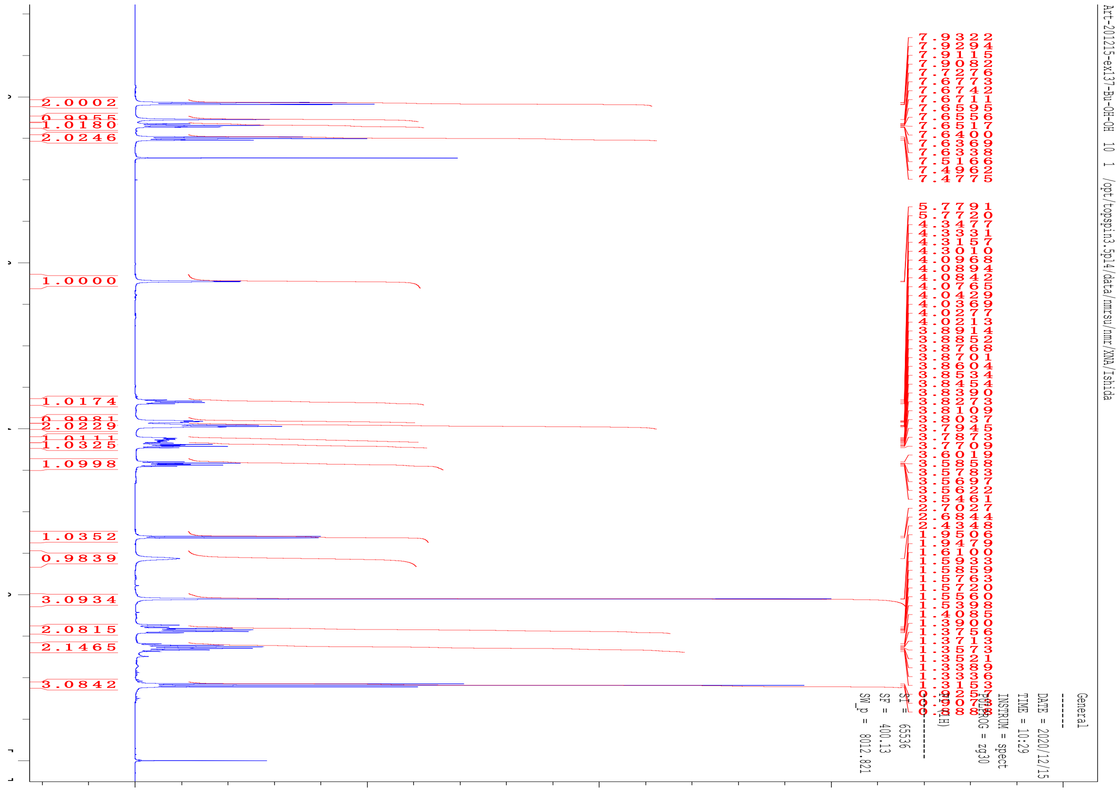

^{13}C NMR, $CDCl_3$, 100 MHz

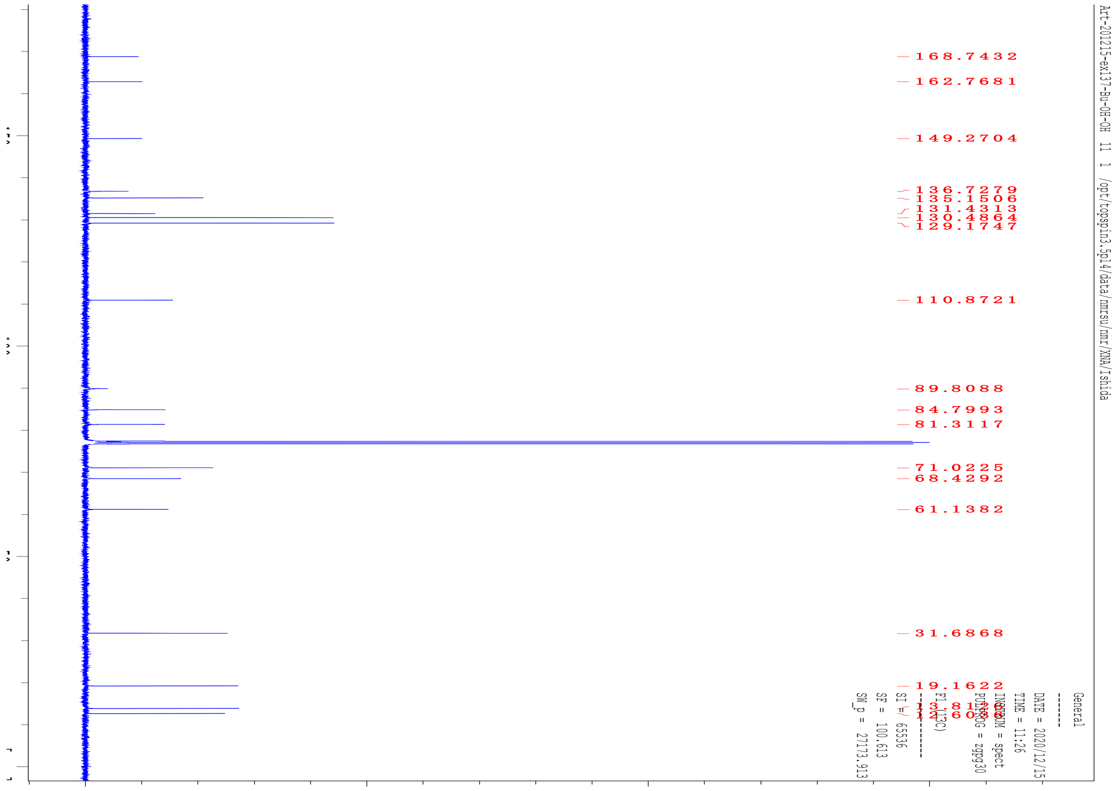

Figure S17. ^{1}H and ^{13}C NMR spectra of compound **3c**.

^{1}H NMR, $CDCl_3$, 400 MHz

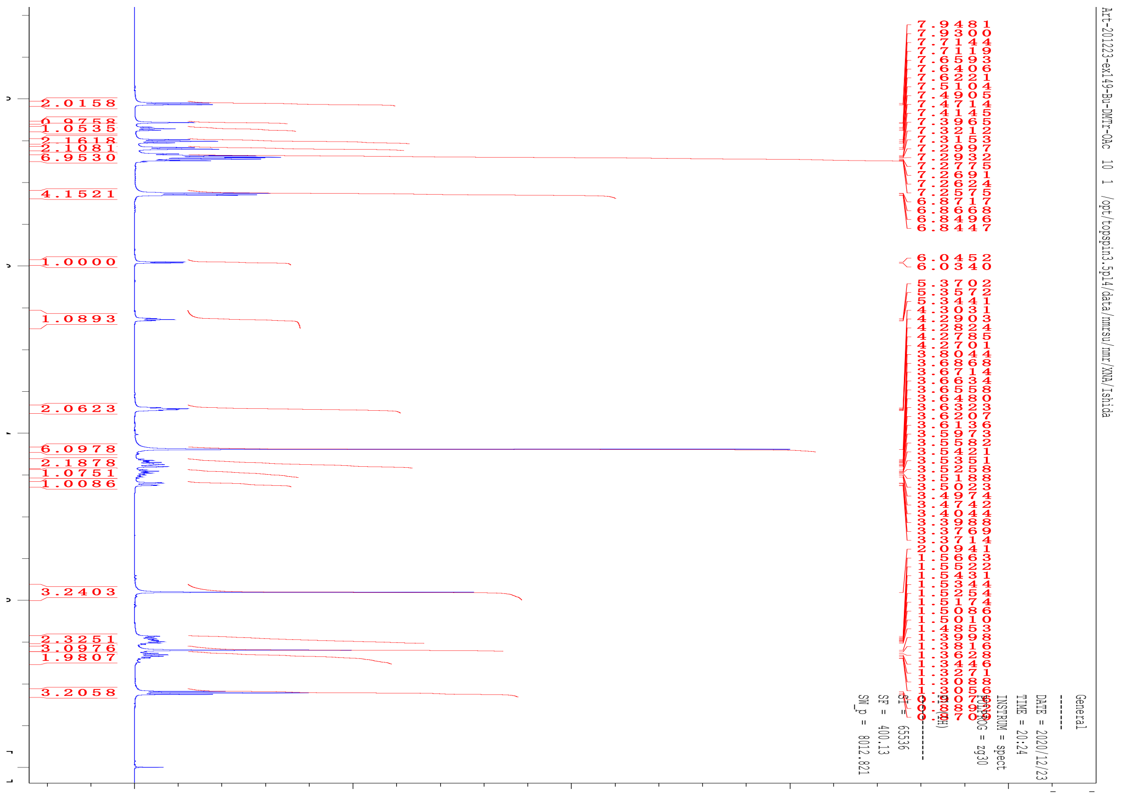

^{13}C NMR, $CDCl_3$, 100 MHz

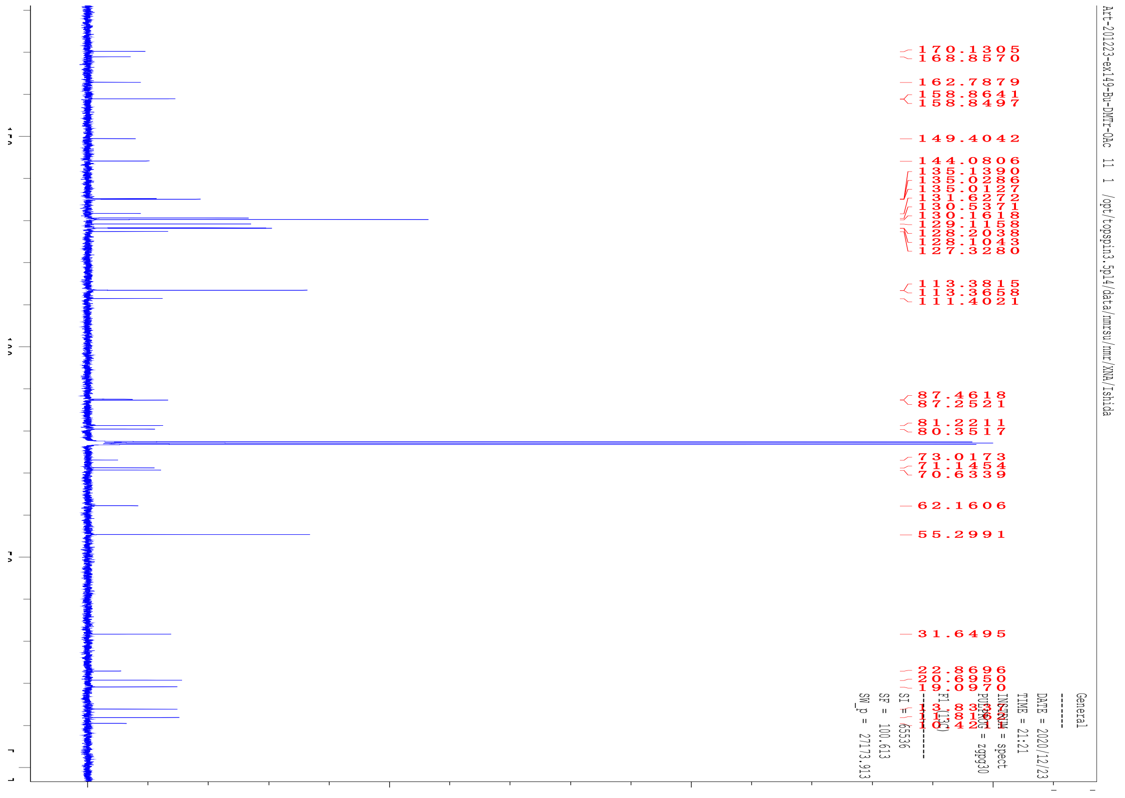

Figure S18. ^{1}H and ^{13}C NMR spectra of compound **4c**.

^{1}H NMR, $CDCl_3$, 400 MHz

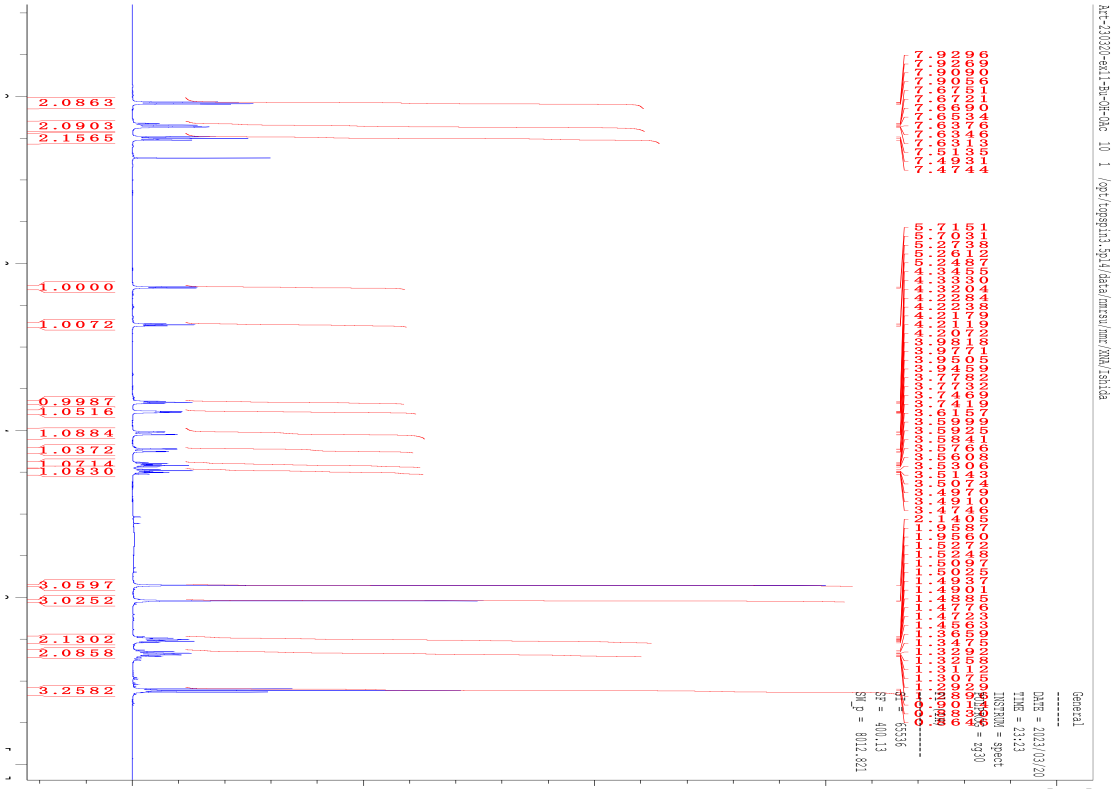

^{13}C NMR, $CDCl_3$, 100 MHz

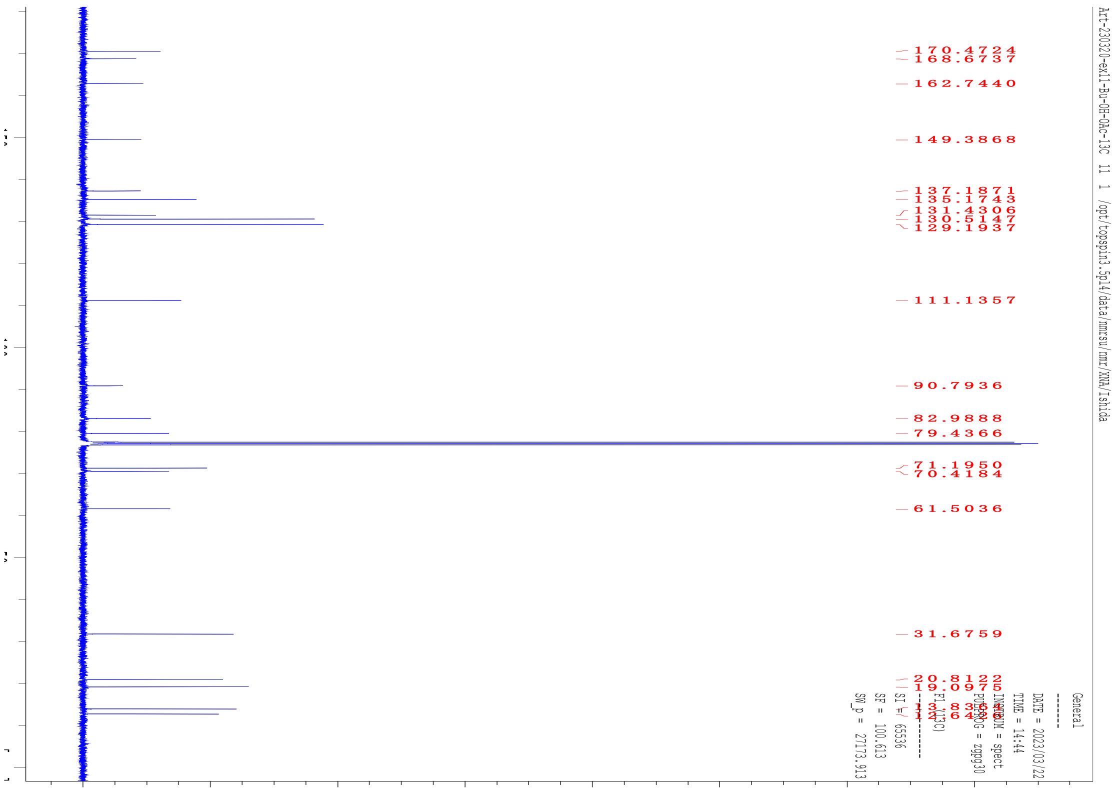

Figure S19. ^{1}H and ^{13}C NMR spectra of compound **5c**.

^{1}H NMR, D_2O, 400 MHz

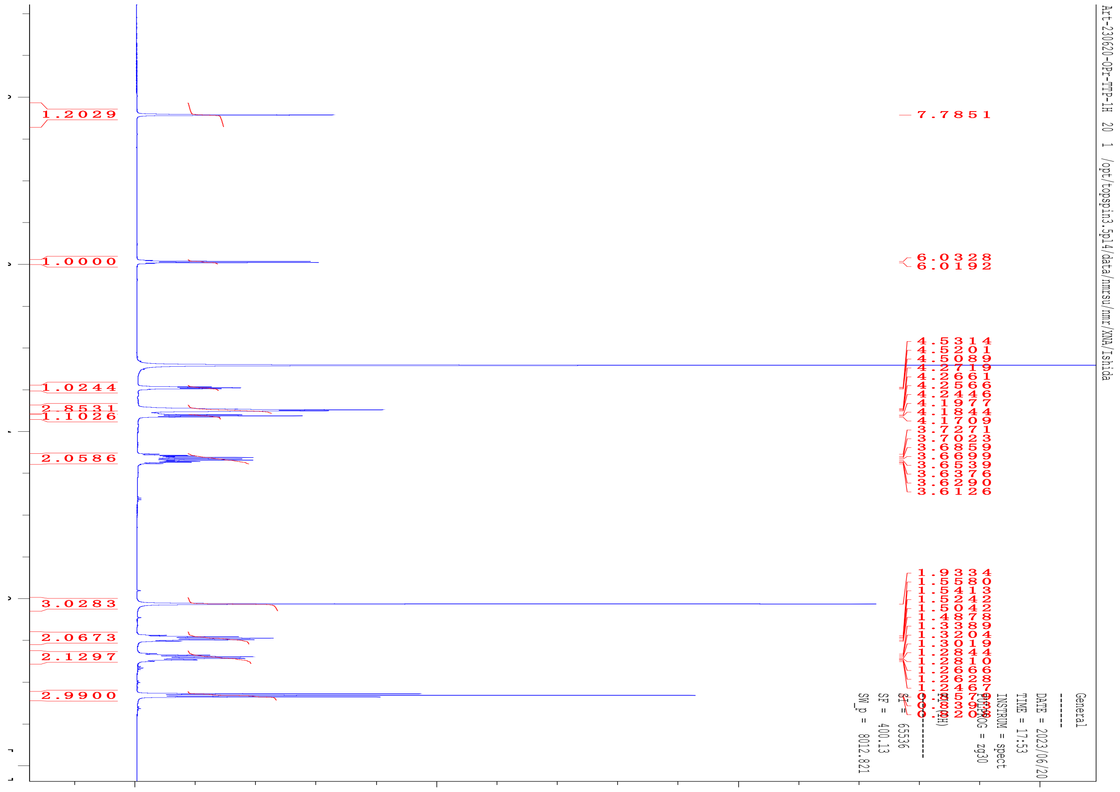

^{31}P NMR, D_2O, 120 MHz

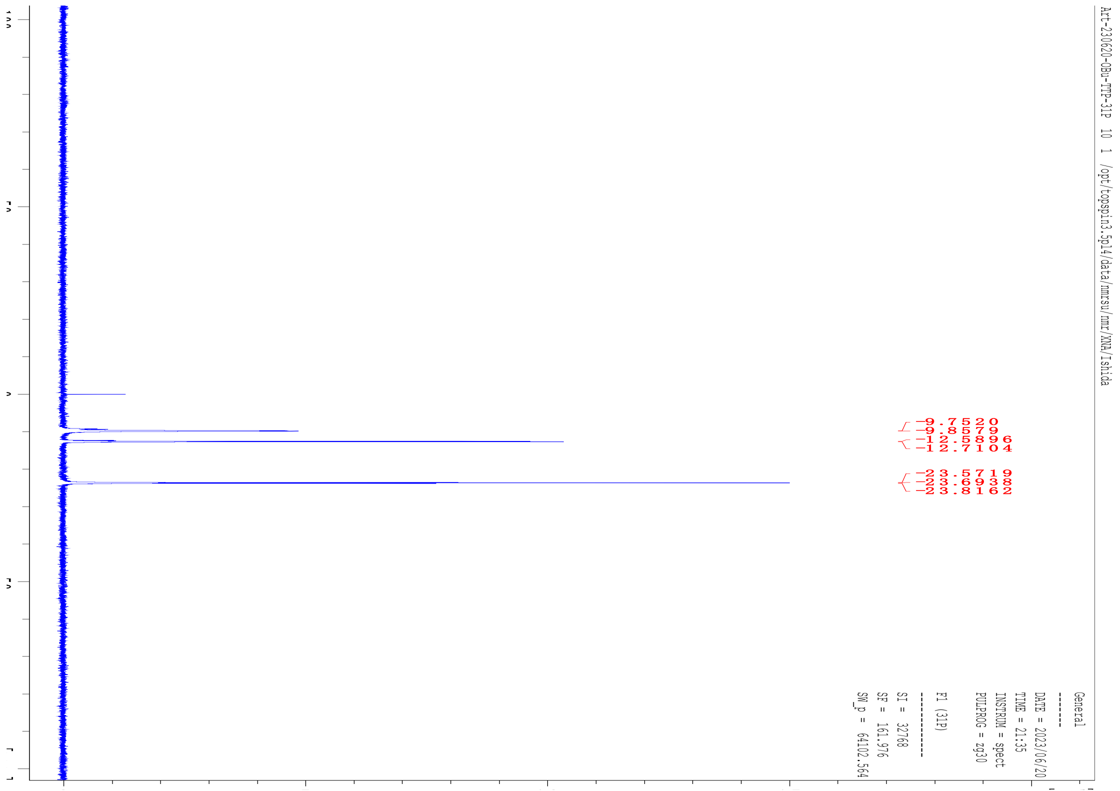

Figure S20. ^{1}H and ^{31}P NMR spectra of compound **6c**.

^{1}H NMR, $CDCl_3$, 400 MHz

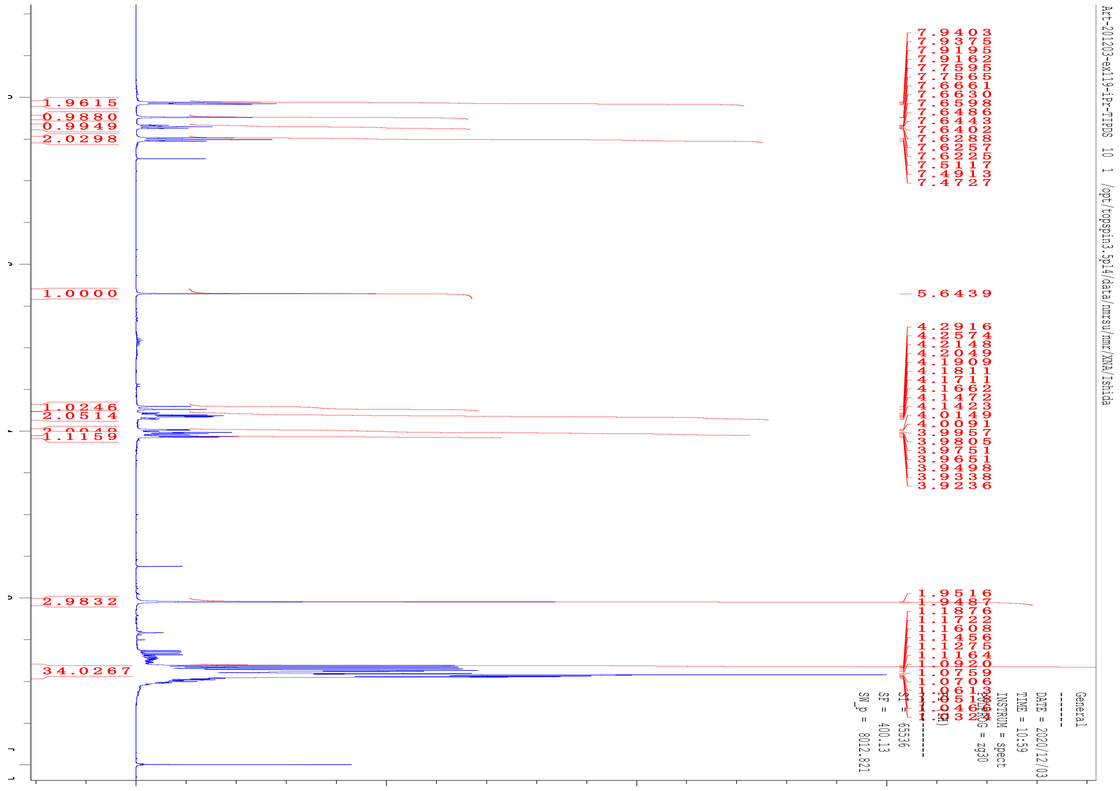

^{13}C NMR, $CDCl_3$, 100 MHz

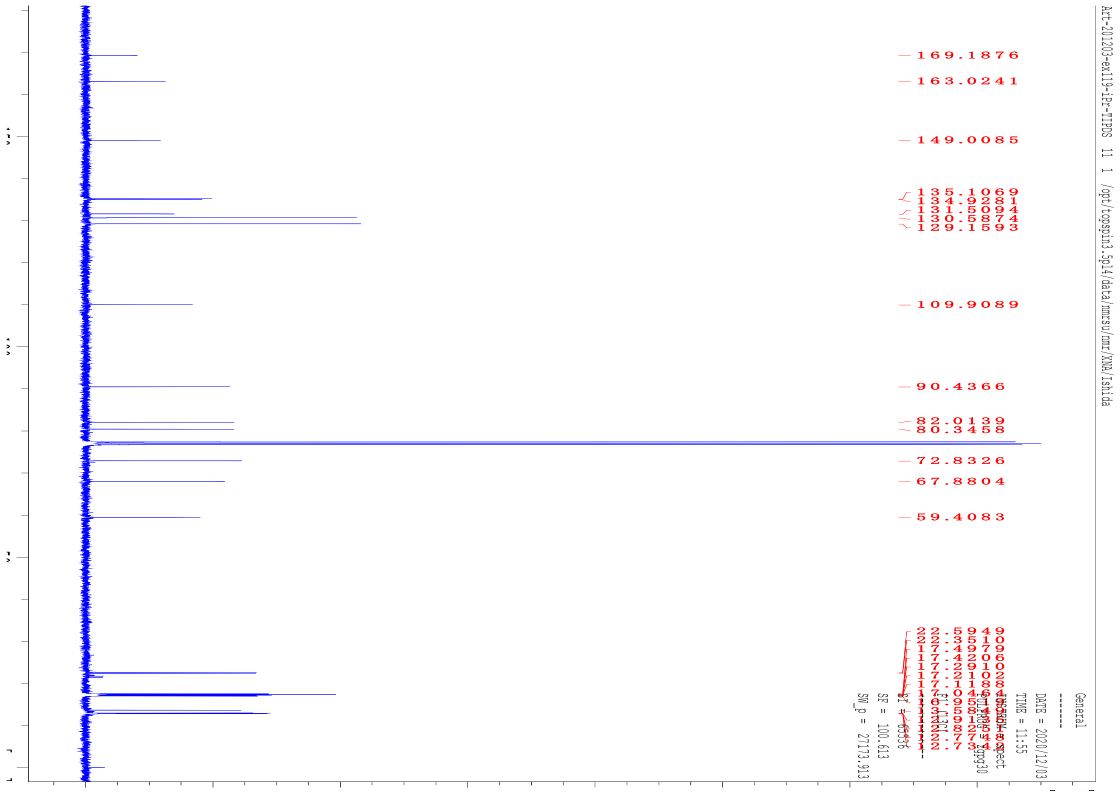

Figure S21. ^{1}H and ^{13}C NMR spectra of compound **2d**.

^{1}H NMR, $CDCl_3$, 400 MHz

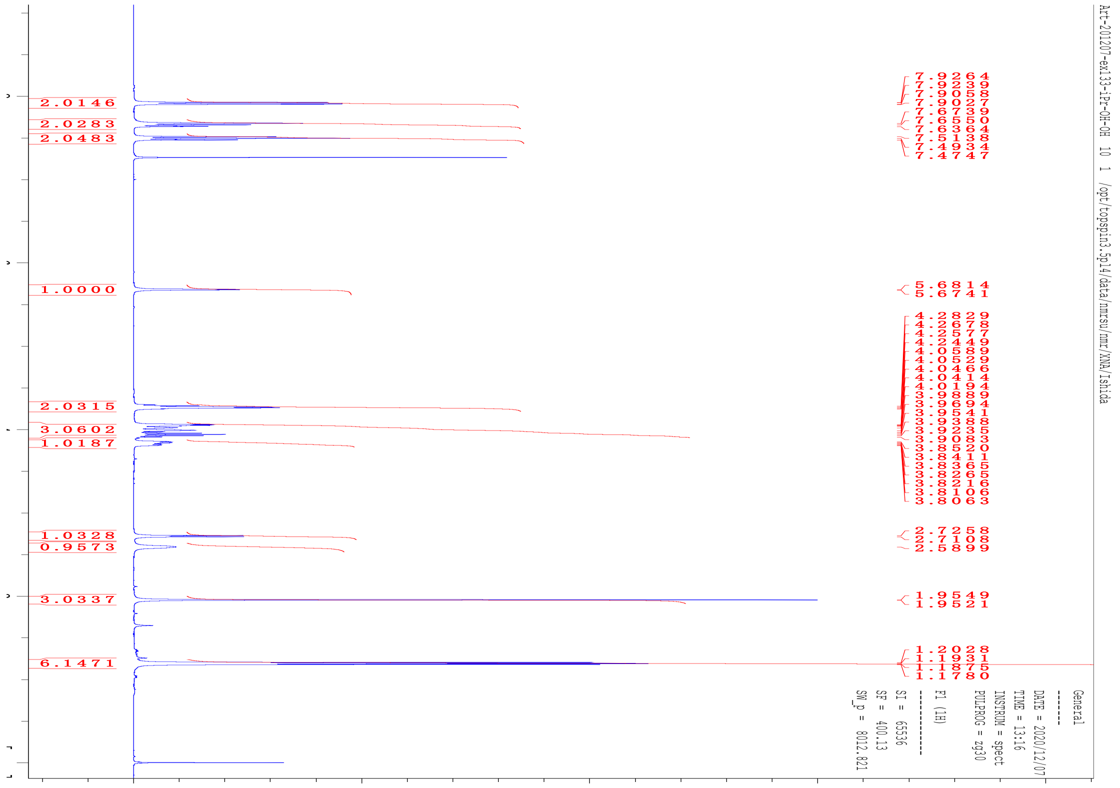

^{13}C NMR, $CDCl_3$, 100 MHz

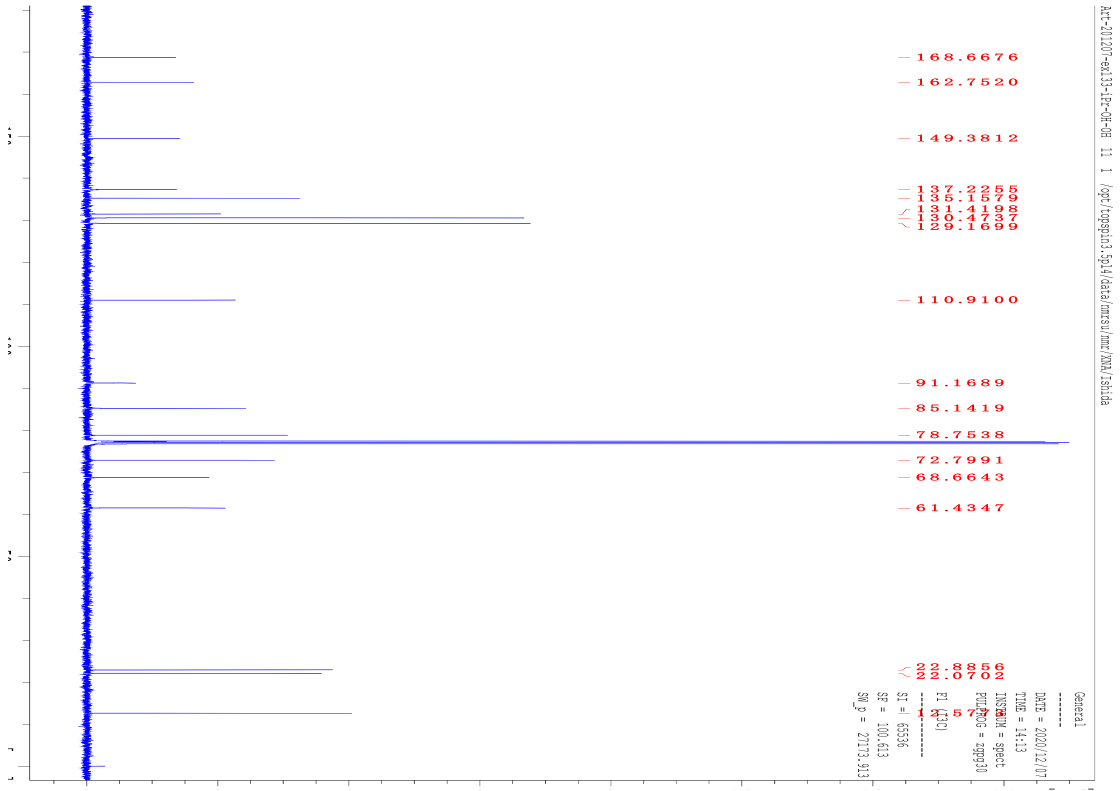

Figure S22. ^{1}H and ^{13}C NMR spectra of compound **3d**.

^{1}H NMR, $CDCl_3$, 400 MHz

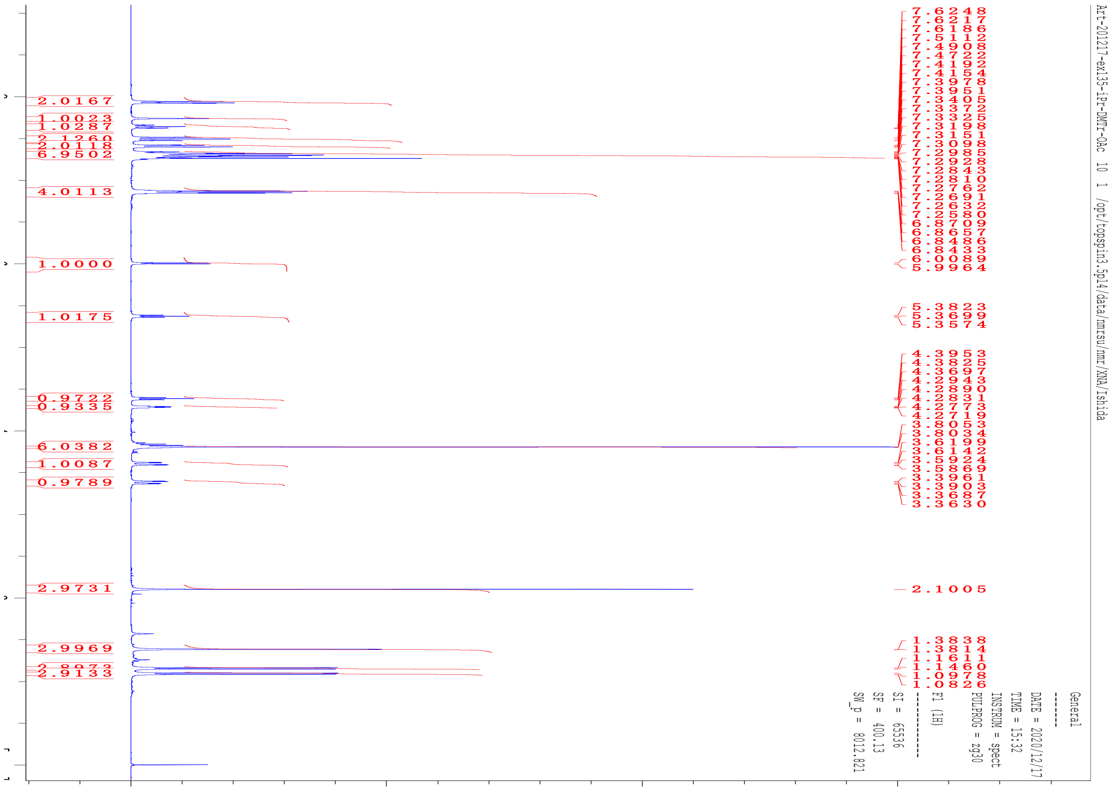

^{13}C NMR, $CDCl_3$, 100 MHz

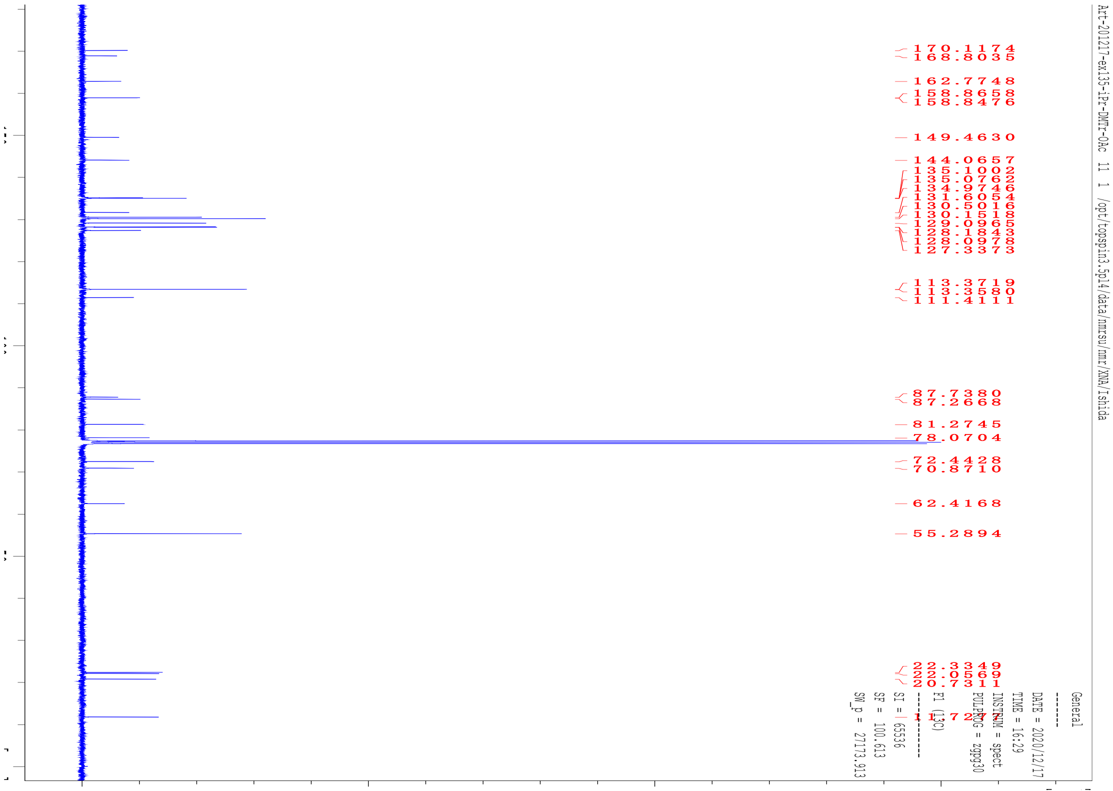

Figure S23. ^{1}H and ^{13}C NMR spectra of compound **4d**.

Compound **5d** (^{1}H NMR, $CDCl_3$, 400 MHz)

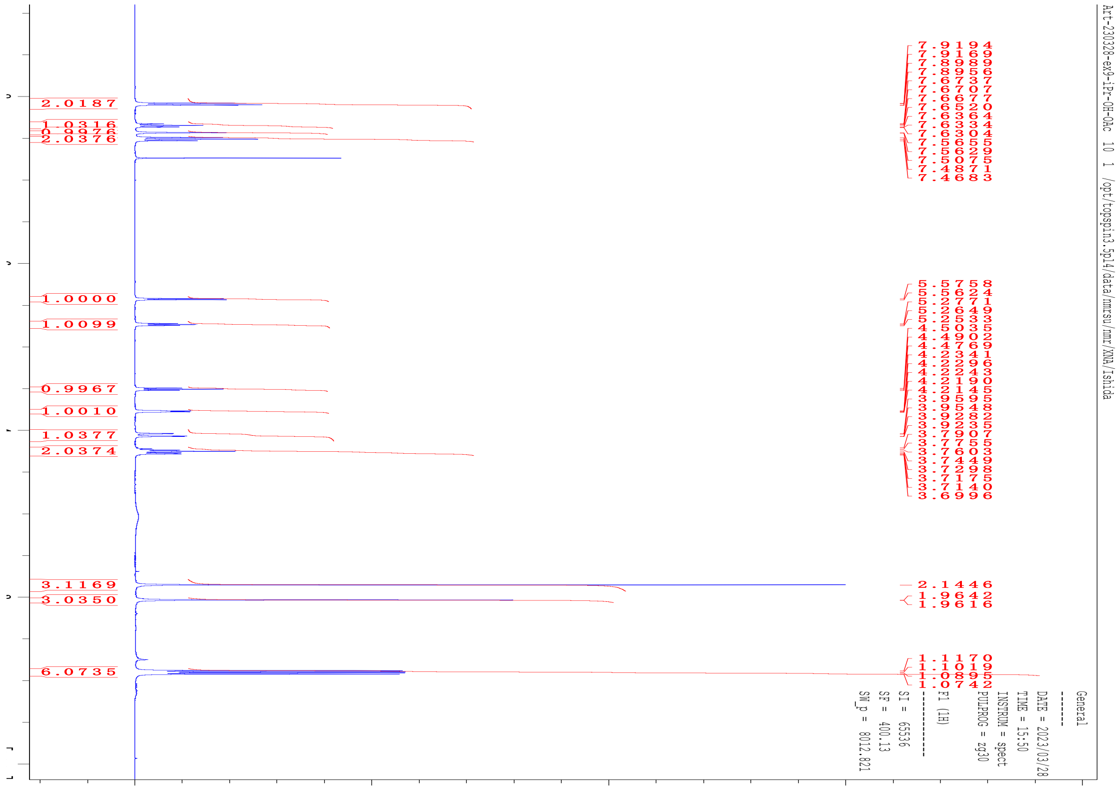

Compound **5d** (^{13}C NMR, $CDCl_3$, 100 MHz)

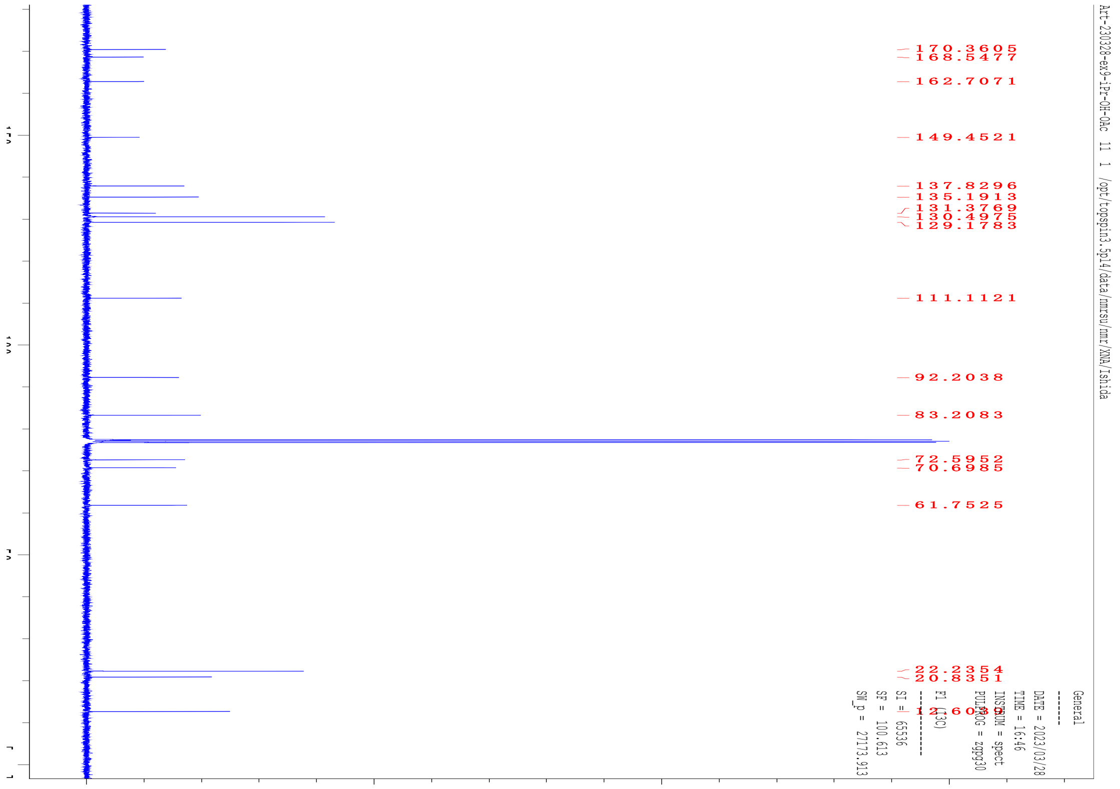

Figure S24. ^{1}H and ^{13}C NMR spectra of compound **5d**.

Compound **6d** (^{1}H NMR, D_2O, 400 MHz)

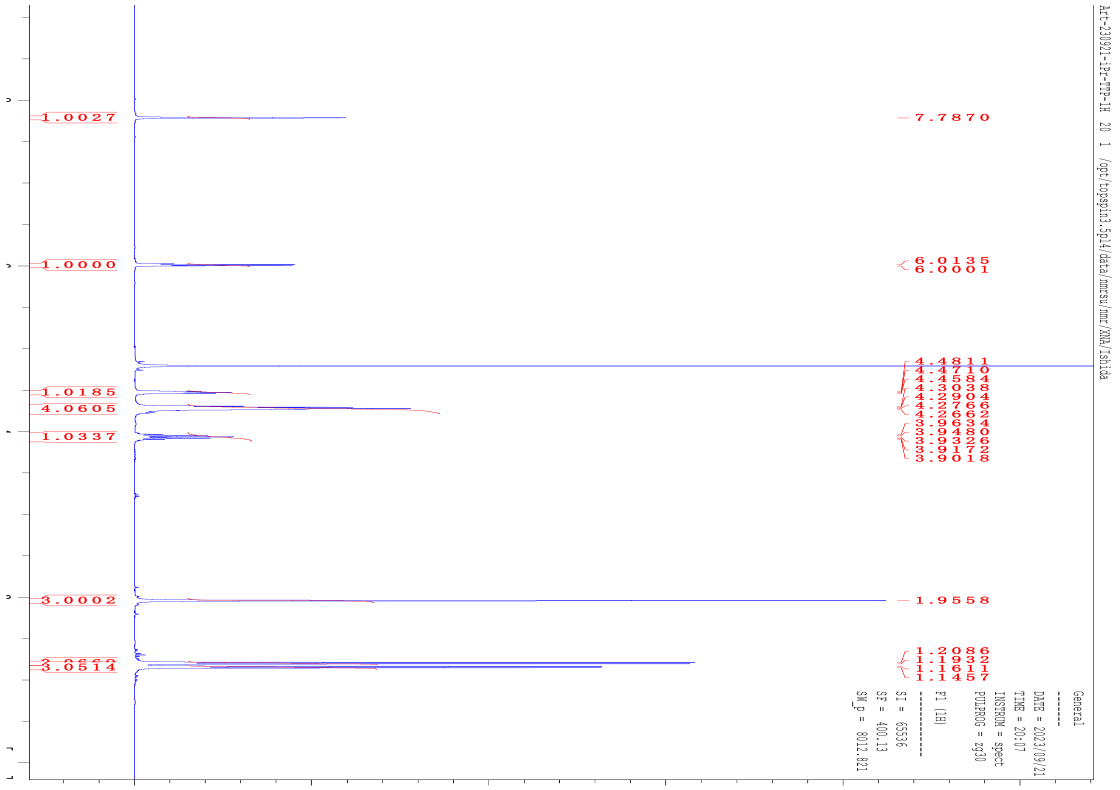

Compound **6d** (^{31}P NMR, D_2O, 120 MHz)

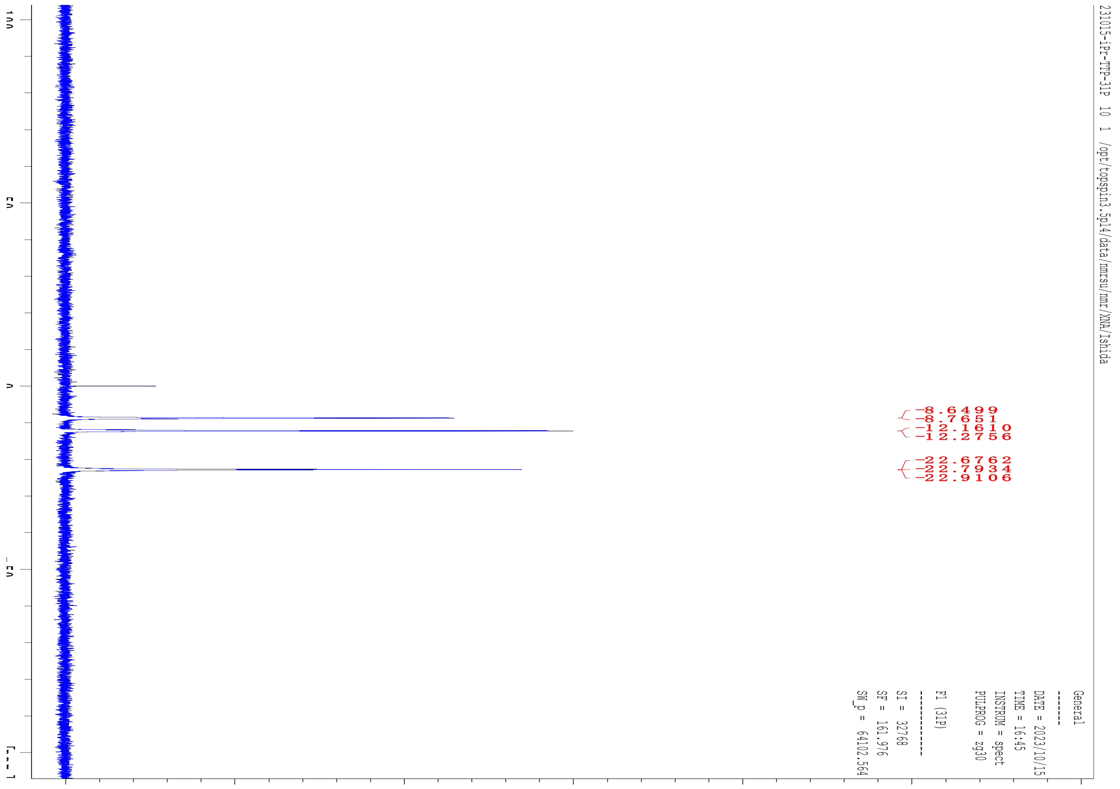

Figure S25. ^{1}H and ^{31}P NMR spectra of compound **6d**.

^{1}H NMR, DMSO-d_6, 400 MHz

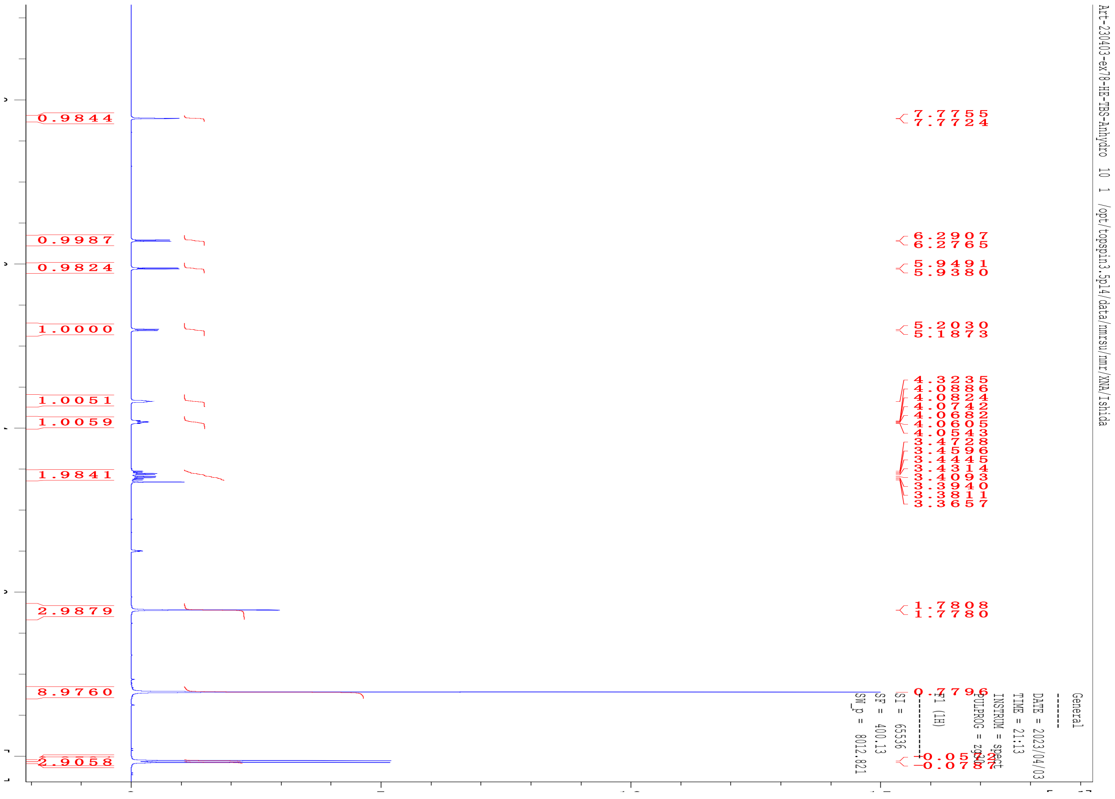

^{13}C NMR, DMSO-d_6, 100 MHz

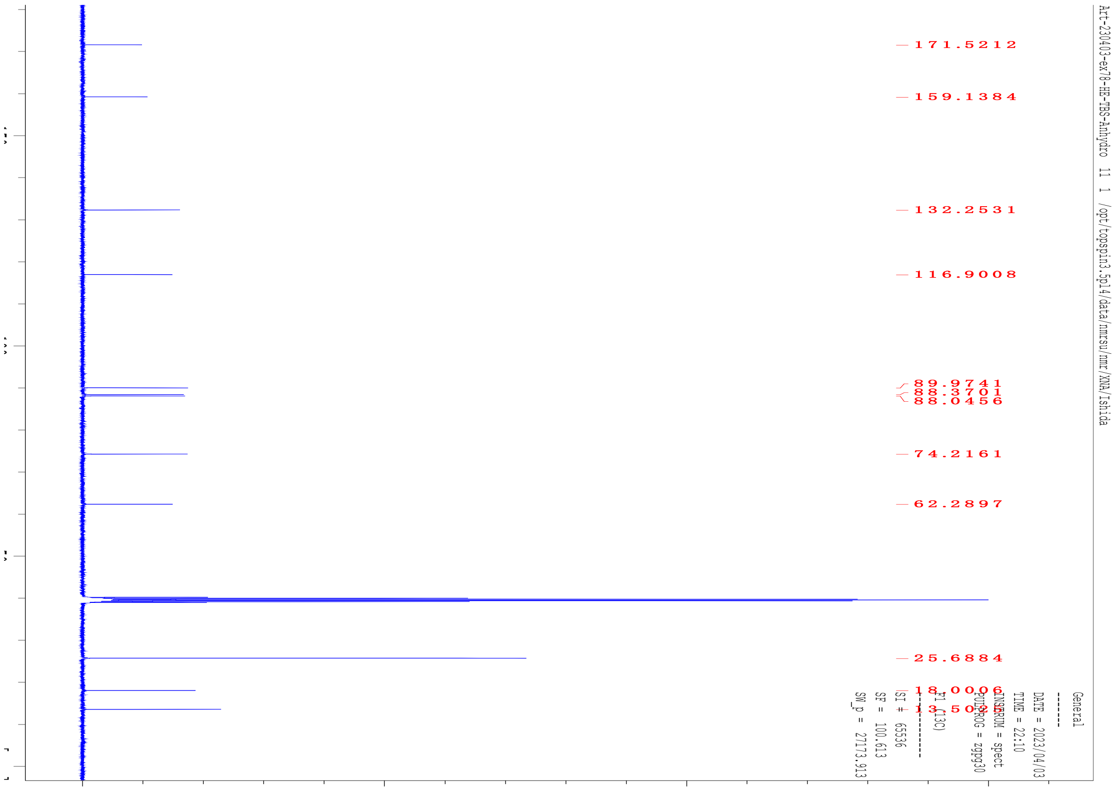

Figure S26. ^{1}H and ^{13}C NMR spectra of compound **8**.

^{1}H NMR, $CDCl_3$, 400 MHz

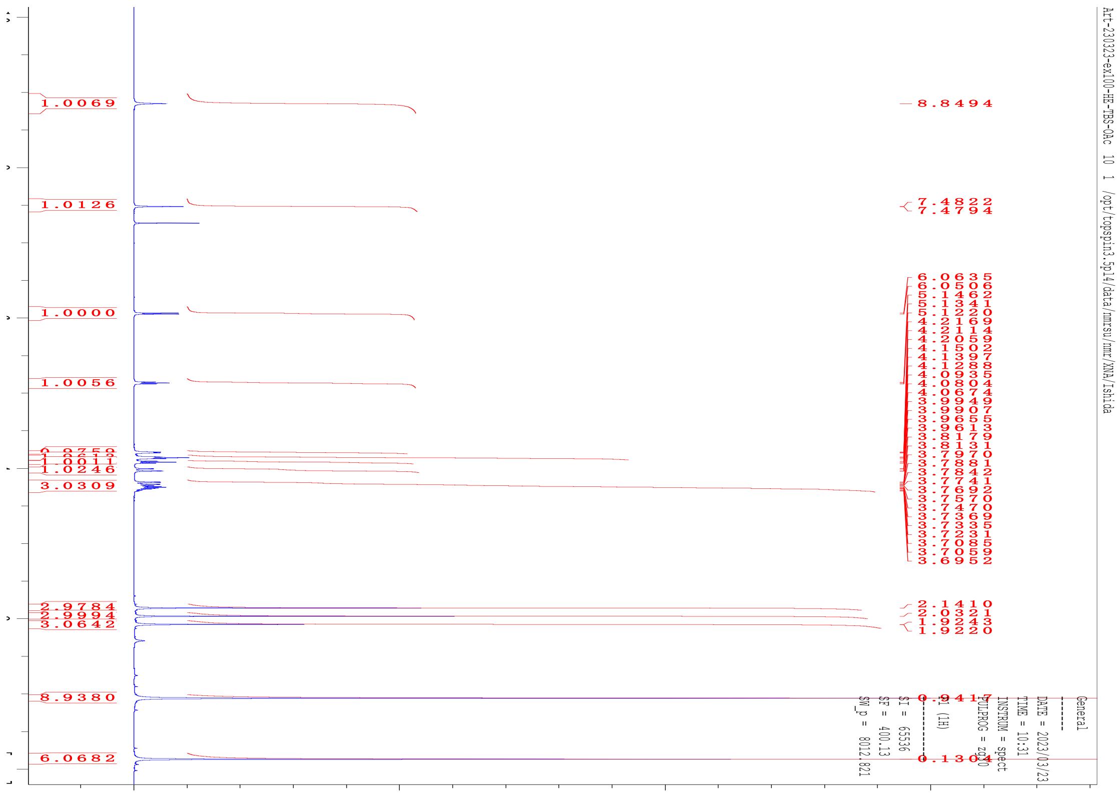

^{13}C NMR, $CDCl_3$, 100 MHz

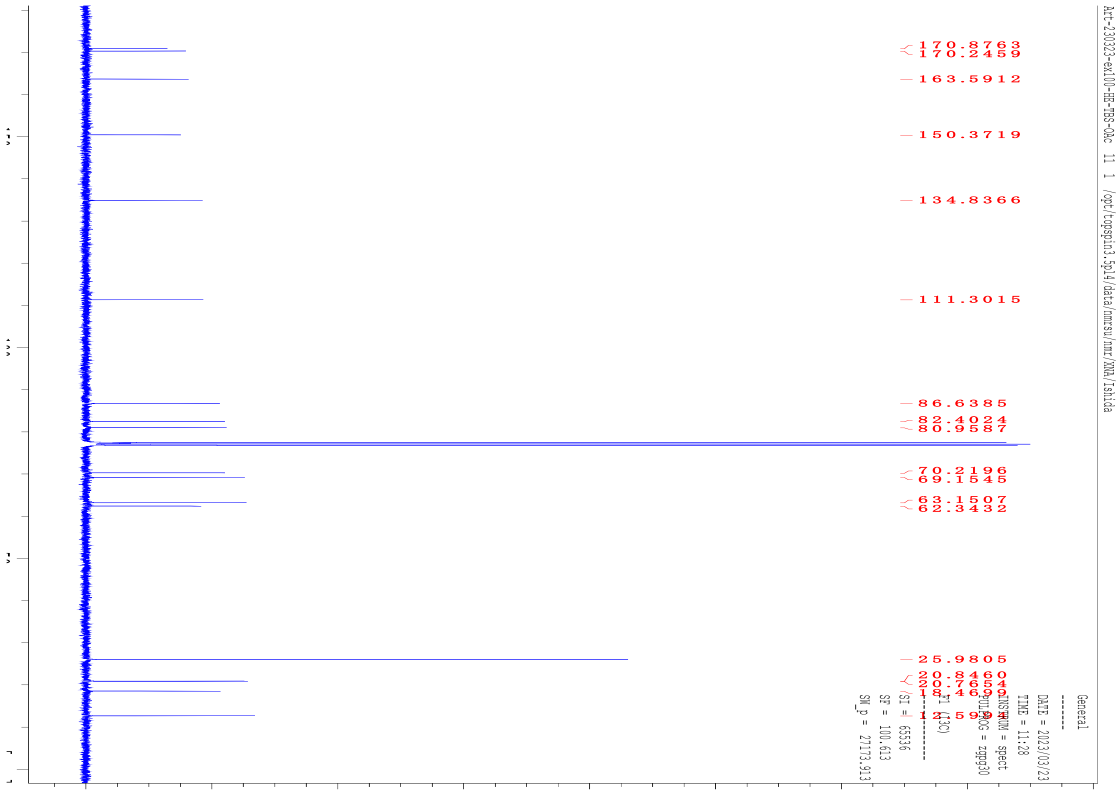

Figure S27. ^{1}H and ^{13}C NMR spectra of compound **9**.

^{1}H NMR, $CDCl_3$, 400 MHz

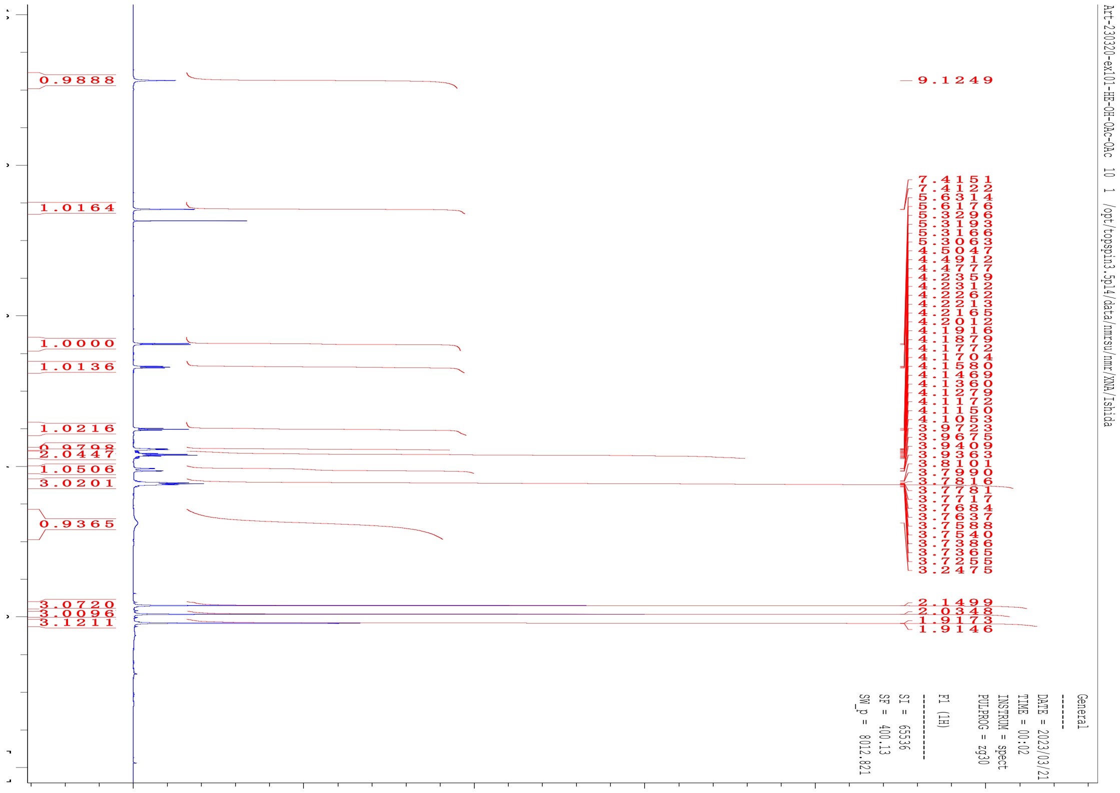

^{13}C NMR, $CDCl_3$, 100 MHz

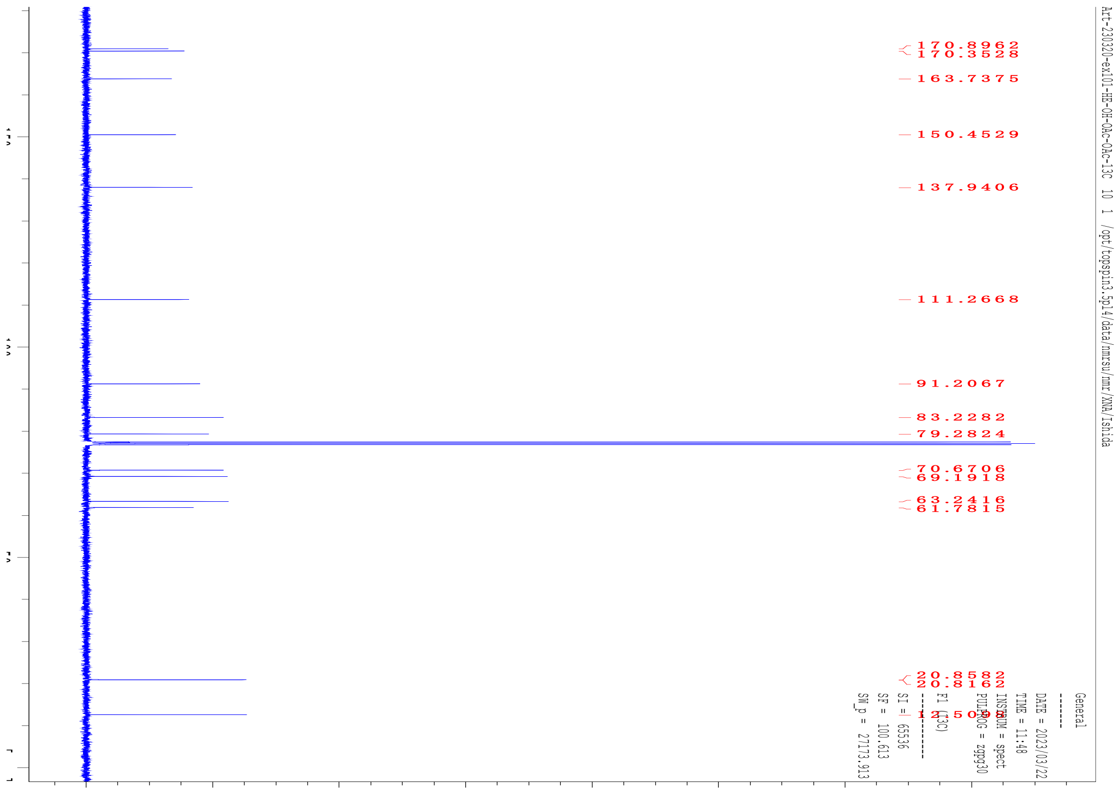

Figure S28. ^{1}H and ^{13}C NMR spectra of compound **10**.

Compound **11** (^1H NMR, D_2O, 400 MHz)

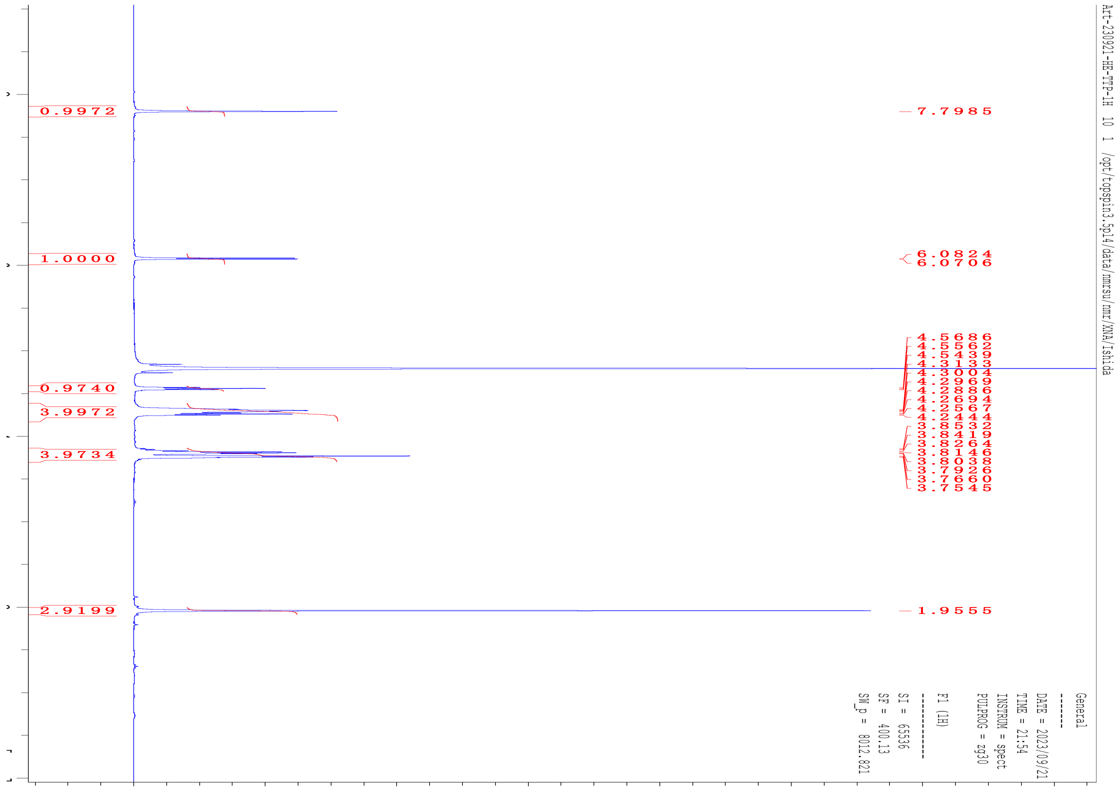

Compound **11** (^{31}P NMR, D_2O, 120 MHz)

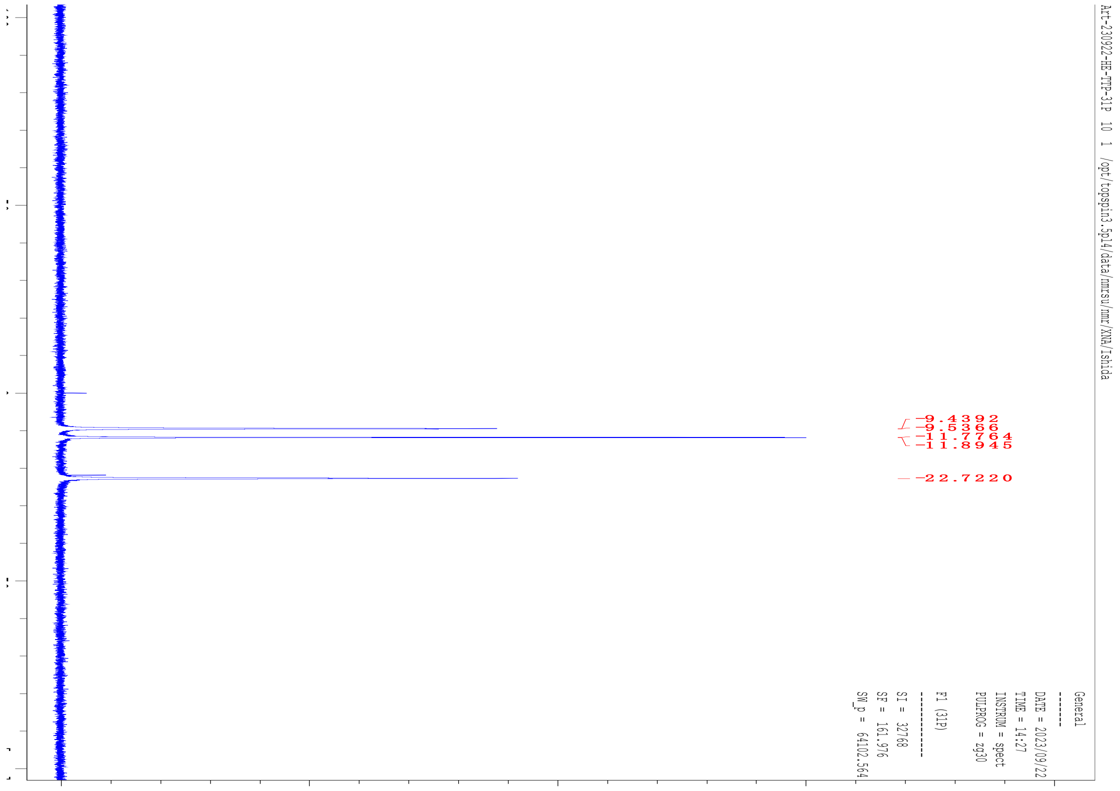

Figure S29. ^1H and ^{31}P NMR spectra of compound **11**.

Compound **13** (^{1}H NMR, D_2O, 400 MHz)

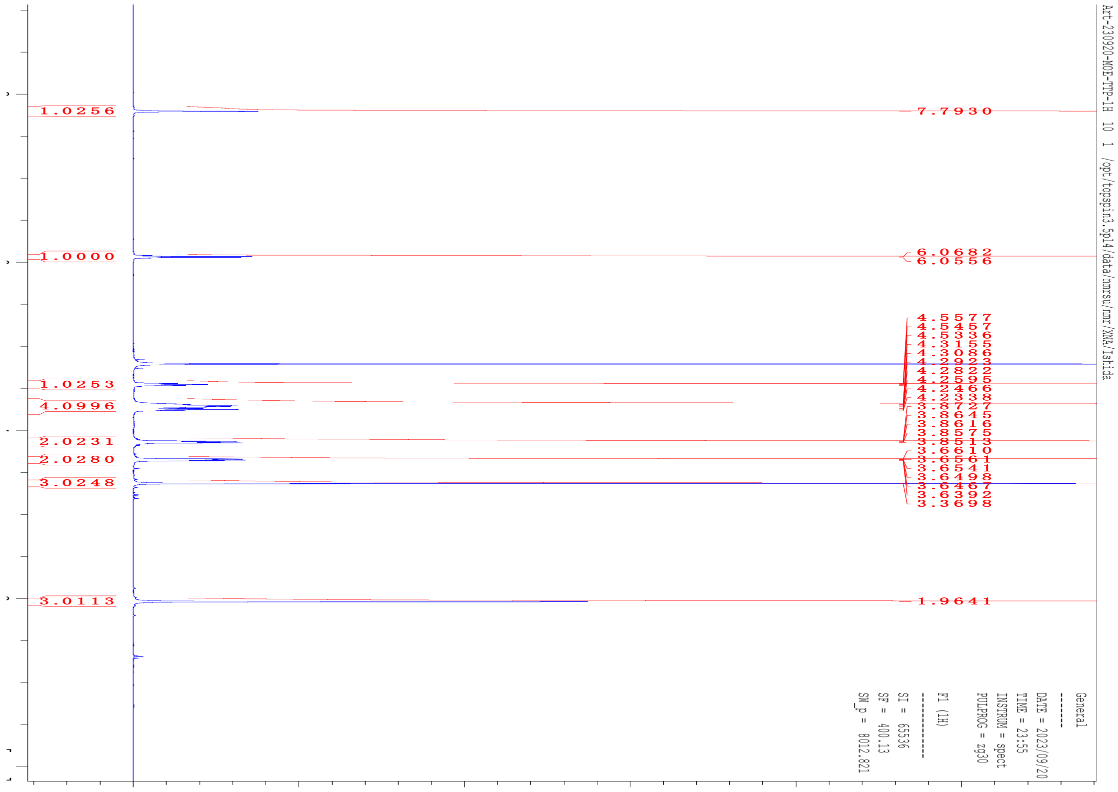

Compound **13** (^{31}P NMR, D_2O, 120 MHz)

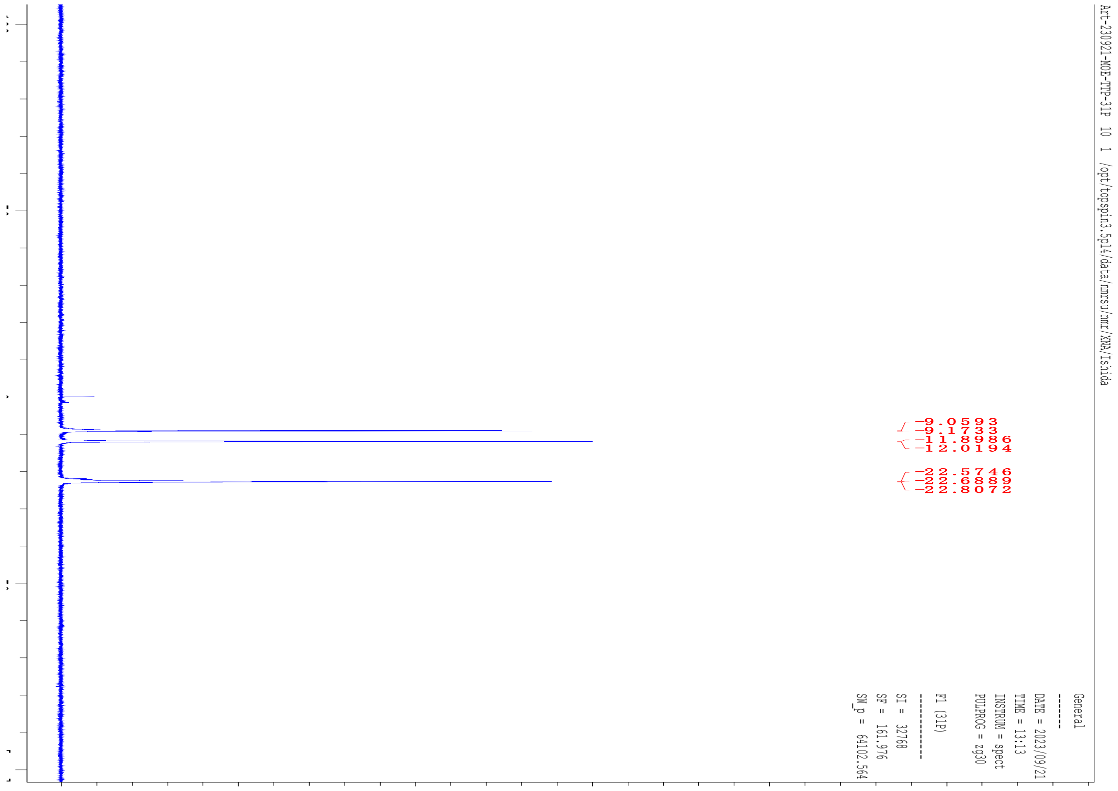

Figure S30. ^{1}H and ^{31}P NMR spectra of compound **13**.
